# Supplementary material for: Heparinase I treatment to overcome RNA quantification interference in heparinized liver donor samples: One size fits all?
Source: PLoS One. 2025 May 12;20(5):e0322899. doi: 10.1371/journal.pone.0322899 (PMC12068581; doi:10.1371/journal.pone.0322899)
Supplement: S1 Table — (DOCX) [file pone.0322899.s001.docx]

**S1 Table. miRNA Ct values in donor liver biopsies.**

(A) Global endogen miRNAs and Cel-miR-39 Ct means and standard deviations in eight liver donors. (B) Endogen miRNAs and Cel-miR-39 Ct means, and standard deviations divided by DBD (n=4) and DCD (n=4) donors. (C) Raw data Ct triplicates in liver tissue biopsies without heparinase (NoHep), with 6 IU (Hep6U) and 12 IU (Hep12U).

**A.**

| **miRNAs** | **n** | **Ct mean** | **Ct SD** |
| --- | --- | --- | --- |
| miR-122 | 8 | 21.72 | 0.29 |
| miR-148a | 8 | 25.26 | 0.58 |
| miR-103a | 8 | 26.48 | 0.43 |
| miR-191 | 8 | 28.13 | 0.43 |
| Cel-miR-39 | 8 | 29.54 | 0.30 |

**B.**

| **Donor group** | **miRNAs** | **n** | **Ct mean** | **Ct SD** |
| --- | --- | --- | --- | --- |
| DBD | miR-122 | 4 | 21.78 | 0.40 |
|  | miR-148a | 4 | 25.52 | 0.60 |
|  | miR-103a | 4 | 26.64 | 0.41 |
|  | miR-191 | 4 | 28.24 | 0.40 |
|  | Cel-miR-39 | 4 | 29.63 | 0.39 |
| DCD | miR-122 | 4 | 21.66 | 0.18 |
|  | miR-148a | 4 | 25.00 | 0.48 |
|  | miR-103a | 4 | 26.31 | 0.43 |
|  | miR-191 | 4 | 28.03 | 0.49 |
|  | Cel-miR-39 | 4 | 29.44 | 0.19 |

**C.**

| **miRNAs** | **Donor group** | **Treatment** | **Sample Name** | **Ct** |
| --- | --- | --- | --- | --- |
| miR122 | DCD | NoHep | DCD.T.NoHep_THEP8 | 21.57 |
| miR122 | DCD | NoHep | DCD.T.NoHep_THEP8 | 21.47 |
| miR122 | DCD | NoHep | DCD.T.NoHep_THEP8 | 21.56 |
| miR148 | DCD | NoHep | DCD.T.NoHep_THEP8 | 24.55 |
| miR148 | DCD | NoHep | DCD.T.NoHep_THEP8 | 24.56 |
| miR148 | DCD | NoHep | DCD.T.NoHep_THEP8 | 24.59 |
| miR103 | DCD | NoHep | DCD.T.NoHep_THEP8 | 25.72 |
| miR103 | DCD | NoHep | DCD.T.NoHep_THEP8 | 25.76 |
| miR103 | DCD | NoHep | DCD.T.NoHep_THEP8 | 25.8 |
| miR191 | DCD | NoHep | DCD.T.NoHep_THEP8 | 27.43 |
| miR191 | DCD | NoHep | DCD.T.NoHep_THEP8 | 27.51 |
| miR191 | DCD | NoHep | DCD.T.NoHep_THEP8 | 27.53 |
| miR39 | DCD | NoHep | DCD.T.NoHep_THEP8 | 28.98 |
| miR39 | DCD | NoHep | DCD.T.NoHep_THEP8 | 29.21 |
| miR39 | DCD | NoHep | DCD.T.NoHep_THEP8 | 29.3 |
| miR122 | DBD | NoHep | DBD.T.NoHep_THEP22 | 22.19 |
| miR122 | DBD | NoHep | DBD.T.NoHep_THEP22 | 22.11 |
| miR122 | DBD | NoHep | DBD.T.NoHep_THEP22 | 22.16 |
| miR148 | DBD | NoHep | DBD.T.NoHep_THEP22 | 26.06 |
| miR148 | DBD | NoHep | DBD.T.NoHep_THEP22 | 26 |
| miR148 | DBD | NoHep | DBD.T.NoHep_THEP22 | 26.14 |
| miR103 | DBD | NoHep | DBD.T.NoHep_THEP22 | 27.69 |
| miR103 | DBD | NoHep | DBD.T.NoHep_THEP22 | 27.08 |
| miR103 | DBD | NoHep | DBD.T.NoHep_THEP22 | 27.11 |
| miR191 | DBD | NoHep | DBD.T.NoHep_THEP22 | 28.85 |
| miR191 | DBD | NoHep | DBD.T.NoHep_THEP22 | 28.68 |
| miR191 | DBD | NoHep | DBD.T.NoHep_THEP22 | 28.73 |
| miR39 | DBD | NoHep | DBD.T.NoHep_THEP22 | 29.61 |
| miR39 | DBD | NoHep | DBD.T.NoHep_THEP22 | 30.14 |
| miR39 | DBD | NoHep | DBD.T.NoHep_THEP22 | 29.75 |
| miR122 | DBD | NoHep | DBD.T.NoHep_THEP26 | 21.34 |
| miR122 | DBD | NoHep | DBD.T.NoHep_THEP26 | 21.32 |
| miR122 | DBD | NoHep | DBD.T.NoHep_THEP26 | 21.7 |
| miR148 | DBD | NoHep | DBD.T.NoHep_THEP26 | 25.08 |
| miR148 | DBD | NoHep | DBD.T.NoHep_THEP26 | 24.91 |
| miR148 | DBD | NoHep | DBD.T.NoHep_THEP26 | 24.99 |
| miR103 | DBD | NoHep | DBD.T.NoHep_THEP26 | 26.18 |
| miR103 | DBD | NoHep | DBD.T.NoHep_THEP26 | 26.56 |
| miR103 | DBD | NoHep | DBD.T.NoHep_THEP26 | 26.57 |
| miR191 | DBD | NoHep | DBD.T.NoHep_THEP26 | 28.12 |
| miR191 | DBD | NoHep | DBD.T.NoHep_THEP26 | 27.79 |
| miR191 | DBD | NoHep | DBD.T.NoHep_THEP26 | 28.27 |
| miR39 | DBD | NoHep | DBD.T.NoHep_THEP26 | 30.04 |
| miR39 | DBD | NoHep | DBD.T.NoHep_THEP26 | 30.08 |
| miR39 | DBD | NoHep | DBD.T.NoHep_THEP26 | 29.88 |
| miR122 | DCD | NoHep | DCD.T.NoHep_THEP33 | 21.48 |
| miR122 | DCD | NoHep | DCD.T.NoHep_THEP33 | 21.59 |
| miR122 | DCD | NoHep | DCD.T.NoHep_THEP33 | 21.46 |
| miR148 | DCD | NoHep | DCD.T.NoHep_THEP33 | 24.98 |
| miR148 | DCD | NoHep | DCD.T.NoHep_THEP33 | 24.92 |
| miR148 | DCD | NoHep | DCD.T.NoHep_THEP33 | 24.9 |
| miR103 | DCD | NoHep | DCD.T.NoHep_THEP33 | 26.2 |
| miR103 | DCD | NoHep | DCD.T.NoHep_THEP33 | 26.07 |
| miR103 | DCD | NoHep | DCD.T.NoHep_THEP33 | 26.26 |
| miR191 | DCD | NoHep | DCD.T.NoHep_THEP33 | 27.53 |
| miR191 | DCD | NoHep | DCD.T.NoHep_THEP33 | 28.05 |
| miR191 | DCD | NoHep | DCD.T.NoHep_THEP33 | 27.8 |
| miR39 | DCD | NoHep | DCD.T.NoHep_THEP33 | 29.26 |
| miR39 | DCD | NoHep | DCD.T.NoHep_THEP33 | 29.34 |
| miR39 | DCD | NoHep | DCD.T.NoHep_THEP33 | 29.79 |
| miR122 | DCD | NoHep | DCD.T.NoHep_THEP34 | 21.77 |
| miR122 | DCD | NoHep | DCD.T.NoHep_THEP34 | 21.71 |
| miR122 | DCD | NoHep | DCD.T.NoHep_THEP34 | 21.67 |
| miR148 | DCD | NoHep | DCD.T.NoHep_THEP34 | 25.57 |
| miR148 | DCD | NoHep | DCD.T.NoHep_THEP34 | 25.76 |
| miR148 | DCD | NoHep | DCD.T.NoHep_THEP34 | 25.74 |
| miR103 | DCD | NoHep | DCD.T.NoHep_THEP34 | 26.61 |
| miR103 | DCD | NoHep | DCD.T.NoHep_THEP34 | 26.69 |
| miR103 | DCD | NoHep | DCD.T.NoHep_THEP34 | 26.73 |
| miR191 | DCD | NoHep | DCD.T.NoHep_THEP34 | 28.11 |
| miR191 | DCD | NoHep | DCD.T.NoHep_THEP34 | 28.13 |
| miR191 | DCD | NoHep | DCD.T.NoHep_THEP34 | 28.46 |
| miR39 | DCD | NoHep | DCD.T.NoHep_THEP34 | 29.35 |
| miR39 | DCD | NoHep | DCD.T.NoHep_THEP34 | 29.7 |
| miR39 | DCD | NoHep | DCD.T.NoHep_THEP34 | 29.62 |
| miR122 | DBD | NoHep | DBD.T.NoHep_THEP36 | 21.48 |
| miR122 | DBD | NoHep | DBD.T.NoHep_THEP36 | 21.25 |
| miR122 | DBD | NoHep | DBD.T.NoHep_THEP36 | 21.52 |
| miR148 | DBD | NoHep | DBD.T.NoHep_THEP36 | 24.99 |
| miR148 | DBD | NoHep | DBD.T.NoHep_THEP36 | 25.04 |
| miR148 | DBD | NoHep | DBD.T.NoHep_THEP36 | 25.02 |
| miR103 | DBD | NoHep | DBD.T.NoHep_THEP36 | 26.28 |
| miR103 | DBD | NoHep | DBD.T.NoHep_THEP36 | 26.16 |
| miR103 | DBD | NoHep | DBD.T.NoHep_THEP36 | 26.11 |
| miR191 | DBD | NoHep | DBD.T.NoHep_THEP36 | 27.74 |
| miR191 | DBD | NoHep | DBD.T.NoHep_THEP36 | 27.87 |
| miR191 | DBD | NoHep | DBD.T.NoHep_THEP36 | 27.85 |
| miR39 | DBD | NoHep | DBD.T.NoHep_THEP36 | 29.02 |
| miR39 | DBD | NoHep | DBD.T.NoHep_THEP36 | 29.25 |
| miR39 | DBD | NoHep | DBD.T.NoHep_THEP36 | 29.05 |
| miR122 | DCD | NoHep | DCD.T.NoHep_THEP11 | 21.83 |
| miR122 | DCD | NoHep | DCD.T.NoHep_THEP11 | 21.91 |
| miR122 | DCD | NoHep | DCD.T.NoHep_THEP11 | 21.97 |
| miR148 | DCD | NoHep | DCD.T.NoHep_THEP11 | 24.8 |
| miR148 | DCD | NoHep | DCD.T.NoHep_THEP11 | 24.83 |
| miR148 | DCD | NoHep | DCD.T.NoHep_THEP11 | 24.78 |
| miR103 | DCD | NoHep | DCD.T.NoHep_THEP11 | 26.54 |
| miR103 | DCD | NoHep | DCD.T.NoHep_THEP11 | 26.73 |
| miR103 | DCD | NoHep | DCD.T.NoHep_THEP11 | 26.62 |
| miR191 | DCD | NoHep | DCD.T.NoHep_THEP11 | 28.58 |
| miR191 | DCD | NoHep | DCD.T.NoHep_THEP11 | 28.56 |
| miR191 | DCD | NoHep | DCD.T.NoHep_THEP11 | 28.67 |
| miR39 | DCD | NoHep | DCD.T.NoHep_THEP11 | 29.62 |
| miR39 | DCD | NoHep | DCD.T.NoHep_THEP11 | 29.29 |
| miR39 | DCD | NoHep | DCD.T.NoHep_THEP11 | 29.86 |
| miR122 | DBD | NoHep | DBD.T.NoHep_THEP15 | 22.11 |
| miR122 | DBD | NoHep | DBD.T.NoHep_THEP15 | 22.05 |
| miR122 | DBD | NoHep | DBD.T.NoHep_THEP15 | 22.11 |
| miR148 | DBD | NoHep | DBD.T.NoHep_THEP15 | 25.99 |
| miR148 | DBD | NoHep | DBD.T.NoHep_THEP15 | 25.97 |
| miR148 | DBD | NoHep | DBD.T.NoHep_THEP15 | 26.1 |
| miR103 | DBD | NoHep | DBD.T.NoHep_THEP15 | 26.85 |
| miR103 | DBD | NoHep | DBD.T.NoHep_THEP15 | 26.86 |
| miR103 | DBD | NoHep | DBD.T.NoHep_THEP15 | 26.88 |
| miR191 | DBD | NoHep | DBD.T.NoHep_THEP15 | 28.43 |
| miR191 | DBD | NoHep | DBD.T.NoHep_THEP15 | 28.2 |
| miR191 | DBD | NoHep | DBD.T.NoHep_THEP15 | 28.32 |
| miR39 | DBD | NoHep | DBD.T.NoHep_THEP15 | 29.46 |
| miR39 | DBD | NoHep | DBD.T.NoHep_THEP15 | 29.74 |
| miR39 | DBD | NoHep | DBD.T.NoHep_THEP15 | 29.53 |
| miR122 | DBD | Hep6U | DBD.T.Hep6U_THEP22 | 23.63 |
| miR122 | DBD | Hep6U | DBD.T.Hep6U_THEP22 | 23.57 |
| miR122 | DBD | Hep6U | DBD.T.Hep6U_THEP22 | 23.67 |
| miR148 | DBD | Hep6U | DBD.T.Hep6U_THEP22 | 27.67 |
| miR148 | DBD | Hep6U | DBD.T.Hep6U_THEP22 | 27.57 |
| miR148 | DBD | Hep6U | DBD.T.Hep6U_THEP22 | 27.59 |
| miR103 | DBD | Hep6U | DBD.T.Hep6U_THEP22 | 28.58 |
| miR103 | DBD | Hep6U | DBD.T.Hep6U_THEP22 | 28.53 |
| miR103 | DBD | Hep6U | DBD.T.Hep6U_THEP22 | 28.55 |
| miR191 | DBD | Hep6U | DBD.T.Hep6U_THEP22 | 29.76 |
| miR191 | DBD | Hep6U | DBD.T.Hep6U_THEP22 | 29.8 |
| miR191 | DBD | Hep6U | DBD.T.Hep6U_THEP22 | 30 |
| miR39 | DBD | Hep6U | DBD.T.Hep6U_THEP22 | 29.78 |
| miR39 | DBD | Hep6U | DBD.T.Hep6U_THEP22 | 29.22 |
| miR39 | DBD | Hep6U | DBD.T.Hep6U_THEP22 | 29.46 |
| miR122 | DBD | Hep6U | DBD.T.Hep6U_THEP26 | 22.34 |
| miR122 | DBD | Hep6U | DBD.T.Hep6U_THEP26 | 22.5 |
| miR122 | DBD | Hep6U | DBD.T.Hep6U_THEP26 | 22.45 |
| miR148 | DBD | Hep6U | DBD.T.Hep6U_THEP26 | 26.49 |
| miR148 | DBD | Hep6U | DBD.T.Hep6U_THEP26 | 26.49 |
| miR148 | DBD | Hep6U | DBD.T.Hep6U_THEP26 | 26.6 |
| miR103 | DBD | Hep6U | DBD.T.Hep6U_THEP26 | 27.58 |
| miR103 | DBD | Hep6U | DBD.T.Hep6U_THEP26 | 27.51 |
| miR103 | DBD | Hep6U | DBD.T.Hep6U_THEP26 | 27.23 |
| miR191 | DBD | Hep6U | DBD.T.Hep6U_THEP26 | 28.63 |
| miR191 | DBD | Hep6U | DBD.T.Hep6U_THEP26 | 28.46 |
| miR191 | DBD | Hep6U | DBD.T.Hep6U_THEP26 | 28.49 |
| miR39 | DBD | Hep6U | DBD.T.Hep6U_THEP26 | 29.58 |
| miR39 | DBD | Hep6U | DBD.T.Hep6U_THEP26 | 29.35 |
| miR39 | DBD | Hep6U | DBD.T.Hep6U_THEP26 | 29.69 |
| miR122 | DCD | Hep6U | DCD.T.Hep6U_THEP33 | 22.54 |
| miR122 | DCD | Hep6U | DCD.T.Hep6U_THEP33 | 22.56 |
| miR122 | DCD | Hep6U | DCD.T.Hep6U_THEP33 | 22.57 |
| miR148 | DCD | Hep6U | DCD.T.Hep6U_THEP33 | 26.08 |
| miR148 | DCD | Hep6U | DCD.T.Hep6U_THEP33 | 26.15 |
| miR148 | DCD | Hep6U | DCD.T.Hep6U_THEP33 | 26.23 |
| miR103 | DCD | Hep6U | DCD.T.Hep6U_THEP33 | 27.06 |
| miR103 | DCD | Hep6U | DCD.T.Hep6U_THEP33 | 27.16 |
| miR103 | DCD | Hep6U | DCD.T.Hep6U_THEP33 | 27.22 |
| miR191 | DCD | Hep6U | DCD.T.Hep6U_THEP33 | 28.53 |
| miR191 | DCD | Hep6U | DCD.T.Hep6U_THEP33 | 28.45 |
| miR191 | DCD | Hep6U | DCD.T.Hep6U_THEP33 | 28.54 |
| miR39 | DCD | Hep6U | DCD.T.Hep6U_THEP33 | 29.22 |
| miR39 | DCD | Hep6U | DCD.T.Hep6U_THEP33 | 29.11 |
| miR39 | DCD | Hep6U | DCD.T.Hep6U_THEP33 | 29.04 |
| miR122 | DCD | Hep6U | DCD.T.Hep6U_THEP8 | 23.51 |
| miR122 | DCD | Hep6U | DCD.T.Hep6U_THEP8 | 23.58 |
| miR122 | DCD | Hep6U | DCD.T.Hep6U_THEP8 | 23.34 |
| miR148 | DCD | Hep6U | DCD.T.Hep6U_THEP8 | 26.91 |
| miR148 | DCD | Hep6U | DCD.T.Hep6U_THEP8 | 26.9 |
| miR148 | DCD | Hep6U | DCD.T.Hep6U_THEP8 | 26.84 |
| miR103 | DCD | Hep6U | DCD.T.Hep6U_THEP8 | 27.9 |
| miR103 | DCD | Hep6U | DCD.T.Hep6U_THEP8 | 27.95 |
| miR103 | DCD | Hep6U | DCD.T.Hep6U_THEP8 | 27.96 |
| miR191 | DCD | Hep6U | DCD.T.Hep6U_THEP8 | 28.77 |
| miR191 | DCD | Hep6U | DCD.T.Hep6U_THEP8 | 28.88 |
| miR191 | DCD | Hep6U | DCD.T.Hep6U_THEP8 | 28.88 |
| miR39 | DCD | Hep6U | DCD.T.Hep6U_THEP8 | 29.26 |
| miR39 | DCD | Hep6U | DCD.T.Hep6U_THEP8 | 29.36 |
| miR39 | DCD | Hep6U | DCD.T.Hep6U_THEP8 | 29.32 |
| miR122 | DCD | Hep6U | DCD.T.Hep6U_THEP34 | 22.31 |
| miR122 | DCD | Hep6U | DCD.T.Hep6U_THEP34 | 22.43 |
| miR122 | DCD | Hep6U | DCD.T.Hep6U_THEP34 | 22.33 |
| miR148 | DCD | Hep6U | DCD.T.Hep6U_THEP34 | 26.49 |
| miR148 | DCD | Hep6U | DCD.T.Hep6U_THEP34 | 26.6 |
| miR148 | DCD | Hep6U | DCD.T.Hep6U_THEP34 | 26.46 |
| miR103 | DCD | Hep6U | DCD.T.Hep6U_THEP34 | 27.17 |
| miR103 | DCD | Hep6U | DCD.T.Hep6U_THEP34 | 27.43 |
| miR103 | DCD | Hep6U | DCD.T.Hep6U_THEP34 | 27.29 |
| miR191 | DCD | Hep6U | DCD.T.Hep6U_THEP34 | 28.64 |
| miR191 | DCD | Hep6U | DCD.T.Hep6U_THEP34 | 28.32 |
| miR191 | DCD | Hep6U | DCD.T.Hep6U_THEP34 | 28.63 |
| miR39 | DCD | Hep6U | DCD.T.Hep6U_THEP34 | 29.55 |
| miR39 | DCD | Hep6U | DCD.T.Hep6U_THEP34 | 29.49 |
| miR39 | DCD | Hep6U | DCD.T.Hep6U_THEP34 | 29.51 |
| miR122 | DBD | Hep6U | DBD.T.Hep6U_THEP36 | 22.13 |
| miR122 | DBD | Hep6U | DBD.T.Hep6U_THEP36 | 22.3 |
| miR122 | DBD | Hep6U | DBD.T.Hep6U_THEP36 | 22.28 |
| miR148 | DBD | Hep6U | DBD.T.Hep6U_THEP36 | 26.07 |
| miR148 | DBD | Hep6U | DBD.T.Hep6U_THEP36 | 26.06 |
| miR148 | DBD | Hep6U | DBD.T.Hep6U_THEP36 | 26.05 |
| miR103 | DBD | Hep6U | DBD.T.Hep6U_THEP36 | 27.35 |
| miR103 | DBD | Hep6U | DBD.T.Hep6U_THEP36 | 27.14 |
| miR103 | DBD | Hep6U | DBD.T.Hep6U_THEP36 | 27.1 |
| miR191 | DBD | Hep6U | DBD.T.Hep6U_THEP36 | 28.43 |
| miR191 | DBD | Hep6U | DBD.T.Hep6U_THEP36 | 28.59 |
| miR191 | DBD | Hep6U | DBD.T.Hep6U_THEP36 | 28.59 |
| miR39 | DBD | Hep6U | DBD.T.Hep6U_THEP36 | 29.14 |
| miR39 | DBD | Hep6U | DBD.T.Hep6U_THEP36 | 29.13 |
| miR39 | DBD | Hep6U | DBD.T.Hep6U_THEP36 | 29.12 |
| miR122 | DCD | Hep6U | DCD.T.Hep6U_THEP11 |  |
| miR122 | DCD | Hep6U | DCD.T.Hep6U_THEP11 | 22.23 |
| miR122 | DCD | Hep6U | DCD.T.Hep6U_THEP11 |  |
| miR148 | DCD | Hep6U | DCD.T.Hep6U_THEP11 | 26.19 |
| miR148 | DCD | Hep6U | DCD.T.Hep6U_THEP11 | 26.28 |
| miR148 | DCD | Hep6U | DCD.T.Hep6U_THEP11 | 26.23 |
| miR103 | DCD | Hep6U | DCD.T.Hep6U_THEP11 | 27.87 |
| miR103 | DCD | Hep6U | DCD.T.Hep6U_THEP11 | 27.79 |
| miR103 | DCD | Hep6U | DCD.T.Hep6U_THEP11 | 27.82 |
| miR191 | DCD | Hep6U | DCD.T.Hep6U_THEP11 | 29.16 |
| miR191 | DCD | Hep6U | DCD.T.Hep6U_THEP11 | 29.15 |
| miR191 | DCD | Hep6U | DCD.T.Hep6U_THEP11 | 29.07 |
| miR39 | DCD | Hep6U | DCD.T.Hep6U_THEP11 | 29.35 |
| miR39 | DCD | Hep6U | DCD.T.Hep6U_THEP11 | 29.46 |
| miR39 | DCD | Hep6U | DCD.T.Hep6U_THEP11 | 29.53 |
| miR122 | DBD | Hep6U | DBD.T.Hep6U_THEP15 | 26.64 |
| miR122 | DBD | Hep6U | DBD.T.Hep6U_THEP15 | 26.79 |
| miR122 | DBD | Hep6U | DBD.T.Hep6U_THEP15 | 26.78 |
| miR148 | DBD | Hep6U | DBD.T.Hep6U_THEP15 | 31.84 |
| miR148 | DBD | Hep6U | DBD.T.Hep6U_THEP15 | 32.27 |
| miR148 | DBD | Hep6U | DBD.T.Hep6U_THEP15 | 32.32 |
| miR103 | DBD | Hep6U | DBD.T.Hep6U_THEP15 | 31.54 |
| miR103 | DBD | Hep6U | DBD.T.Hep6U_THEP15 | 31.71 |
| miR103 | DBD | Hep6U | DBD.T.Hep6U_THEP15 | 31.51 |
| miR191 | DBD | Hep6U | DBD.T.Hep6U_THEP15 | 32.31 |
| miR191 | DBD | Hep6U | DBD.T.Hep6U_THEP15 | 32 |
| miR191 | DBD | Hep6U | DBD.T.Hep6U_THEP15 | 31.98 |
| miR39 | DBD | Hep6U | DBD.T.Hep6U_THEP15 | 29.78 |
| miR39 | DBD | Hep6U | DBD.T.Hep6U_THEP15 | 29.67 |
| miR39 | DBD | Hep6U | DBD.T.Hep6U_THEP15 | 29.72 |
| miR122 | DBD | Hep12U | DBD.T.Hep12U_THEP22 | 24.01 |
| miR122 | DBD | Hep12U | DBD.T.Hep12U_THEP22 | 23.76 |
| miR122 | DBD | Hep12U | DBD.T.Hep12U_THEP22 | 23.68 |
| miR148 | DBD | Hep12U | DBD.T.Hep12U_THEP22 | 28.03 |
| miR148 | DBD | Hep12U | DBD.T.Hep12U_THEP22 | 27.95 |
| miR148 | DBD | Hep12U | DBD.T.Hep12U_THEP22 | 27.81 |
| miR103 | DBD | Hep12U | DBD.T.Hep12U_THEP22 | 29.19 |
| miR103 | DBD | Hep12U | DBD.T.Hep12U_THEP22 | 28.8 |
| miR103 | DBD | Hep12U | DBD.T.Hep12U_THEP22 | 28.93 |
| miR191 | DBD | Hep12U | DBD.T.Hep12U_THEP22 | 30.04 |
| miR191 | DBD | Hep12U | DBD.T.Hep12U_THEP22 | 30.63 |
| miR191 | DBD | Hep12U | DBD.T.Hep12U_THEP22 | 29.55 |
| miR39 | DBD | Hep12U | DBD.T.Hep12U_THEP22 | 30.32 |
| miR39 | DBD | Hep12U | DBD.T.Hep12U_THEP22 | 30.11 |
| miR39 | DBD | Hep12U | DBD.T.Hep12U_THEP22 | 29.74 |
| miR122 | DBD | Hep12U | DBD.T.Hep12U_THEP26 | 22.93 |
| miR122 | DBD | Hep12U | DBD.T.Hep12U_THEP26 | 22.95 |
| miR122 | DBD | Hep12U | DBD.T.Hep12U_THEP26 | 22.9 |
| miR148 | DBD | Hep12U | DBD.T.Hep12U_THEP26 | 26.98 |
| miR148 | DBD | Hep12U | DBD.T.Hep12U_THEP26 | 26.92 |
| miR148 | DBD | Hep12U | DBD.T.Hep12U_THEP26 | 26.85 |
| miR103 | DBD | Hep12U | DBD.T.Hep12U_THEP26 | 27.95 |
| miR103 | DBD | Hep12U | DBD.T.Hep12U_THEP26 | 27.86 |
| miR103 | DBD | Hep12U | DBD.T.Hep12U_THEP26 | 28.11 |
| miR191 | DBD | Hep12U | DBD.T.Hep12U_THEP26 | 29.02 |
| miR191 | DBD | Hep12U | DBD.T.Hep12U_THEP26 | 29.08 |
| miR191 | DBD | Hep12U | DBD.T.Hep12U_THEP26 | 29.11 |
| miR39 | DBD | Hep12U | DBD.T.Hep12U_THEP26 | 29.72 |
| miR39 | DBD | Hep12U | DBD.T.Hep12U_THEP26 | 29.56 |
| miR39 | DBD | Hep12U | DBD.T.Hep12U_THEP26 | 29.71 |
| miR122 | DCD | Hep12U | DCD.T.Hep12U_THEP33 | 22.99 |
| miR122 | DCD | Hep12U | DCD.T.Hep12U_THEP33 | 22.94 |
| miR122 | DCD | Hep12U | DCD.T.Hep12U_THEP33 | 23.02 |
| miR148 | DCD | Hep12U | DCD.T.Hep12U_THEP33 | 26.71 |
| miR148 | DCD | Hep12U | DCD.T.Hep12U_THEP33 | 26.74 |
| miR148 | DCD | Hep12U | DCD.T.Hep12U_THEP33 | 26.61 |
| miR103 | DCD | Hep12U | DCD.T.Hep12U_THEP33 | 27.8 |
| miR103 | DCD | Hep12U | DCD.T.Hep12U_THEP33 | 27.82 |
| miR103 | DCD | Hep12U | DCD.T.Hep12U_THEP33 | 27.79 |
| miR191 | DCD | Hep12U | DCD.T.Hep12U_THEP33 | 29.06 |
| miR191 | DCD | Hep12U | DCD.T.Hep12U_THEP33 | 28.91 |
| miR191 | DCD | Hep12U | DCD.T.Hep12U_THEP33 | 28.98 |
| miR39 | DCD | Hep12U | DCD.T.Hep12U_THEP33 | 29.81 |
| miR39 | DCD | Hep12U | DCD.T.Hep12U_THEP33 | 29.59 |
| miR39 | DCD | Hep12U | DCD.T.Hep12U_THEP33 | 29.69 |
| miR122 | DCD | Hep12U | DCD.T.Hep12U_THEP34 | 22.31 |
| miR122 | DCD | Hep12U | DCD.T.Hep12U_THEP34 | 22.54 |
| miR122 | DCD | Hep12U | DCD.T.Hep12U_THEP34 | 22.48 |
| miR148 | DCD | Hep12U | DCD.T.Hep12U_THEP34 | 26.64 |
| miR148 | DCD | Hep12U | DCD.T.Hep12U_THEP34 | 26.72 |
| miR148 | DCD | Hep12U | DCD.T.Hep12U_THEP34 | 26.77 |
| miR103 | DCD | Hep12U | DCD.T.Hep12U_THEP34 | 27.6 |
| miR103 | DCD | Hep12U | DCD.T.Hep12U_THEP34 | 27.6 |
| miR103 | DCD | Hep12U | DCD.T.Hep12U_THEP34 | 27.55 |
| miR191 | DCD | Hep12U | DCD.T.Hep12U_THEP34 | 28.55 |
| miR191 | DCD | Hep12U | DCD.T.Hep12U_THEP34 | 28.97 |
| miR191 | DCD | Hep12U | DCD.T.Hep12U_THEP34 | 28.71 |
| miR39 | DCD | Hep12U | DCD.T.Hep12U_THEP34 | 29.62 |
| miR39 | DCD | Hep12U | DCD.T.Hep12U_THEP34 | 29.27 |
| miR39 | DCD | Hep12U | DCD.T.Hep12U_THEP34 | 29.48 |
| miR122 | DBD | Hep12U | DBD.T.Hep12U_THEP36 | 22.78 |
| miR122 | DBD | Hep12U | DBD.T.Hep12U_THEP36 | 22.68 |
| miR122 | DBD | Hep12U | DBD.T.Hep12U_THEP36 | 22.73 |
| miR148 | DBD | Hep12U | DBD.T.Hep12U_THEP36 | 26.3 |
| miR148 | DBD | Hep12U | DBD.T.Hep12U_THEP36 | 26.43 |
| miR148 | DBD | Hep12U | DBD.T.Hep12U_THEP36 | 26.33 |
| miR103 | DBD | Hep12U | DBD.T.Hep12U_THEP36 | 27.45 |
| miR103 | DBD | Hep12U | DBD.T.Hep12U_THEP36 | 27.52 |
| miR103 | DBD | Hep12U | DBD.T.Hep12U_THEP36 | 27.53 |
| miR191 | DBD | Hep12U | DBD.T.Hep12U_THEP36 | 28.84 |
| miR191 | DBD | Hep12U | DBD.T.Hep12U_THEP36 | 28.84 |
| miR191 | DBD | Hep12U | DBD.T.Hep12U_THEP36 | 28.89 |
| miR39 | DBD | Hep12U | DBD.T.Hep12U_THEP36 | 29.35 |
| miR39 | DBD | Hep12U | DBD.T.Hep12U_THEP36 | 29.55 |
| miR39 | DBD | Hep12U | DBD.T.Hep12U_THEP36 | 29.65 |
| miR122 | DCD | Hep12U | DCD.T.Hep12U_THEP8 | 23.14 |
| miR122 | DCD | Hep12U | DCD.T.Hep12U_THEP8 | 23.45 |
| miR122 | DCD | Hep12U | DCD.T.Hep12U_THEP8 | 23.47 |
| miR148 | DCD | Hep12U | DCD.T.Hep12U_THEP8 | 26.86 |
| miR148 | DCD | Hep12U | DCD.T.Hep12U_THEP8 | 26.96 |
| miR148 | DCD | Hep12U | DCD.T.Hep12U_THEP8 | 26.93 |
| miR103 | DCD | Hep12U | DCD.T.Hep12U_THEP8 | 27.66 |
| miR103 | DCD | Hep12U | DCD.T.Hep12U_THEP8 | 27.61 |
| miR103 | DCD | Hep12U | DCD.T.Hep12U_THEP8 | 27.51 |
| miR191 | DCD | Hep12U | DCD.T.Hep12U_THEP8 | 28.58 |
| miR191 | DCD | Hep12U | DCD.T.Hep12U_THEP8 | 28.59 |
| miR191 | DCD | Hep12U | DCD.T.Hep12U_THEP8 | 28.56 |
| miR39 | DCD | Hep12U | DCD.T.Hep12U_THEP8 | 29.25 |
| miR39 | DCD | Hep12U | DCD.T.Hep12U_THEP8 | 29.21 |
| miR39 | DCD | Hep12U | DCD.T.Hep12U_THEP8 | 29.12 |
| miR122 | DCD | Hep12U | DCD.T.Hep12U_THEP11 | 22.54 |
| miR122 | DCD | Hep12U | DCD.T.Hep12U_THEP11 | 22.54 |
| miR122 | DCD | Hep12U | DCD.T.Hep12U_THEP11 | 22.54 |
| miR148 | DCD | Hep12U | DCD.T.Hep12U_THEP11 | 25.9 |
| miR148 | DCD | Hep12U | DCD.T.Hep12U_THEP11 | 26.02 |
| miR148 | DCD | Hep12U | DCD.T.Hep12U_THEP11 | 25.94 |
| miR103 | DCD | Hep12U | DCD.T.Hep12U_THEP11 | 27.56 |
| miR103 | DCD | Hep12U | DCD.T.Hep12U_THEP11 | 27.51 |
| miR103 | DCD | Hep12U | DCD.T.Hep12U_THEP11 | 27.58 |
| miR191 | DCD | Hep12U | DCD.T.Hep12U_THEP11 | 28.82 |
| miR191 | DCD | Hep12U | DCD.T.Hep12U_THEP11 | 28.74 |
| miR191 | DCD | Hep12U | DCD.T.Hep12U_THEP11 | 28.98 |
| miR39 | DCD | Hep12U | DCD.T.Hep12U_THEP11 | 29.56 |
| miR39 | DCD | Hep12U | DCD.T.Hep12U_THEP11 | 29.59 |
| miR39 | DCD | Hep12U | DCD.T.Hep12U_THEP11 | 29.51 |
| miR122 | DBD | Hep12U | DBD.T.Hep12U_THEP15 | 29.46 |
| miR122 | DBD | Hep12U | DBD.T.Hep12U_THEP15 | 29.54 |
| miR122 | DBD | Hep12U | DBD.T.Hep12U_THEP15 | 29.53 |
| miR148 | DBD | Hep12U | DBD.T.Hep12U_THEP15 | 34.33 |
| miR148 | DBD | Hep12U | DBD.T.Hep12U_THEP15 | 34.63 |
| miR148 | DBD | Hep12U | DBD.T.Hep12U_THEP15 | 33.85 |
| miR103 | DBD | Hep12U | DBD.T.Hep12U_THEP15 | 35.63 |
| miR103 | DBD | Hep12U | DBD.T.Hep12U_THEP15 | 34.73 |
| miR103 | DBD | Hep12U | DBD.T.Hep12U_THEP15 | 34.66 |
| miR191 | DBD | Hep12U | DBD.T.Hep12U_THEP15 | 34.15 |
| miR191 | DBD | Hep12U | DBD.T.Hep12U_THEP15 | 33.98 |
| miR191 | DBD | Hep12U | DBD.T.Hep12U_THEP15 | 36.02 |
| miR39 | DBD | Hep12U | DBD.T.Hep12U_THEP15 | 29.79 |
| miR39 | DBD | Hep12U | DBD.T.Hep12U_THEP15 | 30.07 |
| miR39 | DBD | Hep12U | DBD.T.Hep12U_THEP15 | 30.02 |
