# Supplementary material for: Heparinase I treatment to overcome RNA quantification interference in heparinized liver donor samples: One size fits all?
Source: PLoS One. 2025 May 12;20(5):e0322899. doi: 10.1371/journal.pone.0322899 (PMC12068581; doi:10.1371/journal.pone.0322899)
Supplement: S2 Table — (DOCX) [file pone.0322899.s002.docx]

**S2 Table.** **miRNA Ct values in donor perfusion fluid**. (A) Global endogen miRNAs and Cel-miR-39 Ct means and standard deviations in eight liver donors. (B) Endogen miRNAs and Cel-miR-39 Ct means, and standard deviations divided by DBD (n=4) and DCD (n=4) donors. (C) Ct raw data triplicates in perfusion fluid without heparinase (NoHep), with 6 IU (Hep6U) and 12 IU (Hep12U).

**A.**

| **miRNAs** | **n** | **Ct mean** | **Ct SD** |
| --- | --- | --- | --- |
| miR-122 | 8 | 25.08 | 1.35 |
| miR-148a | 8 | 27.86 | 1.38 |
| miR-103a | 8 | 27.73 | 2.07 |
| miR-191 | 8 | 29.86 | 1.90 |
| Cel-miR-39 | 8 | 24.77 | 0.27 |

**B.**

| **Donor group** | **miRNAs** | **n** | **Ct mean** | **Ct SD** |
| --- | --- | --- | --- | --- |
| DBD | miR-122 | 4 | 24.78 | 1.44 |
|  | miR-148a | 4 | 27.64 | 1.44 |
|  | miR-103a | 4 | 27.92 | 1.77 |
|  | miR-191 | 4 | 29.86 | 1.67 |
|  | Cel-miR-39 | 4 | 24.69 | 0.13 |
| DCD | miR-122 | 4 | 25.38 | 1.40 |
|  | miR-148a | 4 | 28.08 | 1.50 |
|  | miR-103a | 4 | 27.53 | 2.60 |
|  | miR-191 | 4 | 29.85 | 2.36 |
|  | Cel-miR-39 | 4 | 24.86 | 0.36 |

**C.**

| **miRNA** | **Donor**  **group** | **Treatment** | **Sample Name** | **Ct** |
| --- | --- | --- | --- | --- |
| miR122 | DCD | NoHep | DCD.F.NoHep_THEP11 | 26.25 |
| miR122 | DCD | NoHep | DCD.F.NoHep_THEP11 | 26.25 |
| miR122 | DCD | NoHep | DCD.F.NoHep_THEP11 | 25.69 |
| miR148 | DCD | NoHep | DCD.F.NoHep_THEP11 | 28.72 |
| miR148 | DCD | NoHep | DCD.F.NoHep_THEP11 | 28.26 |
| miR148 | DCD | NoHep | DCD.F.NoHep_THEP11 | 28.61 |
| miR103 | DCD | NoHep | DCD.F.NoHep_THEP11 | 29.63 |
| miR103 | DCD | NoHep | DCD.F.NoHep_THEP11 | 29.39 |
| miR103 | DCD | NoHep | DCD.F.NoHep_THEP11 | 29.54 |
| miR191 | DCD | NoHep | DCD.F.NoHep_THEP11 | 31.26 |
| miR191 | DCD | NoHep | DCD.F.NoHep_THEP11 | 31.6 |
| miR191 | DCD | NoHep | DCD.F.NoHep_THEP11 | 32.76 |
| UNISP4 | DCD | NoHep | DCD.F.NoHep_THEP11 | 30.84 |
| UNISP4 | DCD | NoHep | DCD.F.NoHep_THEP11 | 31.82 |
| UNISP4 | DCD | NoHep | DCD.F.NoHep_THEP11 | 30.8 |
| miR39 | DCD | NoHep | DCD.F.NoHep_THEP11 | 24.82 |
| miR39 | DCD | NoHep | DCD.F.NoHep_THEP11 | 24.92 |
| miR39 | DCD | NoHep | DCD.F.NoHep_THEP11 | 24.95 |
| miR122 | DCD | NoHep | DCD.F.NoHep_THEP33 | 25.37 |
| miR122 | DCD | NoHep | DCD.F.NoHep_THEP33 | 25.46 |
| miR122 | DCD | NoHep | DCD.F.NoHep_THEP33 | 25.4 |
| miR148 | DCD | NoHep | DCD.F.NoHep_THEP33 | 27.96 |
| miR148 | DCD | NoHep | DCD.F.NoHep_THEP33 | 28.46 |
| miR148 | DCD | NoHep | DCD.F.NoHep_THEP33 | 28.58 |
| miR103 | DCD | NoHep | DCD.F.NoHep_THEP33 | 28.33 |
| miR103 | DCD | NoHep | DCD.F.NoHep_THEP33 | 27.58 |
| miR103 | DCD | NoHep | DCD.F.NoHep_THEP33 | 27.56 |
| miR191 | DCD | NoHep | DCD.F.NoHep_THEP33 | 30.52 |
| miR191 | DCD | NoHep | DCD.F.NoHep_THEP33 | 28.41 |
| miR191 | DCD | NoHep | DCD.F.NoHep_THEP33 | 30.53 |
| UNISP4 | DCD | NoHep | DCD.F.NoHep_THEP33 | 29.7 |
| UNISP4 | DCD | NoHep | DCD.F.NoHep_THEP33 | 29.86 |
| UNISP4 | DCD | NoHep | DCD.F.NoHep_THEP33 | 30.43 |
| miR39 | DCD | NoHep | DCD.F.NoHep_THEP33 | 24.76 |
| miR39 | DCD | NoHep | DCD.F.NoHep_THEP33 | 10.31 |
| miR39 | DCD | NoHep | DCD.F.NoHep_THEP33 | 24.74 |
| miR122 | DCD | NoHep | DCD.F.NoHep_THEP34 | 23.37 |
| miR122 | DCD | NoHep | DCD.F.NoHep_THEP34 | 23.46 |
| miR122 | DCD | NoHep | DCD.F.NoHep_THEP34 | 23.35 |
| miR148 | DCD | NoHep | DCD.F.NoHep_THEP34 | 25.87 |
| miR148 | DCD | NoHep | DCD.F.NoHep_THEP34 | 25.87 |
| miR148 | DCD | NoHep | DCD.F.NoHep_THEP34 | 25.99 |
| miR103 | DCD | NoHep | DCD.F.NoHep_THEP34 | 23.89 |
| miR103 | DCD | NoHep | DCD.F.NoHep_THEP34 | 23.82 |
| miR103 | DCD | NoHep | DCD.F.NoHep_THEP34 | 23.81 |
| miR191 | DCD | NoHep | DCD.F.NoHep_THEP34 | 26.37 |
| miR191 | DCD | NoHep | DCD.F.NoHep_THEP34 | 26.4 |
| miR191 | DCD | NoHep | DCD.F.NoHep_THEP34 | 26.32 |
| UNISP4 | DCD | NoHep | DCD.F.NoHep_THEP34 | 31.71 |
| UNISP4 | DCD | NoHep | DCD.F.NoHep_THEP34 | 31.39 |
| UNISP4 | DCD | NoHep | DCD.F.NoHep_THEP34 | 30.83 |
| miR39 | DCD | NoHep | DCD.F.NoHep_THEP34 | 24.53 |
| miR39 | DCD | NoHep | DCD.F.NoHep_THEP34 | 24.43 |
| miR39 | DCD | NoHep | DCD.F.NoHep_THEP34 | 24.4 |
| miR122 | DCD | NoHep | DCD.F.NoHep_THEP8 | 26.49 |
| miR122 | DCD | NoHep | DCD.F.NoHep_THEP8 | 26.54 |
| miR122 | DCD | NoHep | DCD.F.NoHep_THEP8 | 26.33 |
| miR148 | DCD | NoHep | DCD.F.NoHep_THEP8 | 29.46 |
| miR148 | DCD | NoHep | DCD.F.NoHep_THEP8 | 29.26 |
| miR148 | DCD | NoHep | DCD.F.NoHep_THEP8 | 28.8 |
| miR103 | DCD | NoHep | DCD.F.NoHep_THEP8 | 29.39 |
| miR103 | DCD | NoHep | DCD.F.NoHep_THEP8 | 28.86 |
| miR103 | DCD | NoHep | DCD.F.NoHep_THEP8 | 29.32 |
| miR191 | DCD | NoHep | DCD.F.NoHep_THEP8 | 30.97 |
| miR191 | DCD | NoHep | DCD.F.NoHep_THEP8 | 31.77 |
| miR191 | DCD | NoHep | DCD.F.NoHep_THEP8 | 31.24 |
| UNISP4 | DCD | NoHep | DCD.F.NoHep_THEP8 | 30.61 |
| UNISP4 | DCD | NoHep | DCD.F.NoHep_THEP8 | 30.94 |
| UNISP4 | DCD | NoHep | DCD.F.NoHep_THEP8 | 30.76 |
| miR39 | DCD | NoHep | DCD.F.NoHep_THEP8 | 25.33 |
| miR39 | DCD | NoHep | DCD.F.NoHep_THEP8 | 25.31 |
| miR39 | DCD | NoHep | DCD.F.NoHep_THEP8 | 25.34 |
| miR122 | DBD | NoHep | DBD.F.NoHep_THEP15 | 26.88 |
| miR122 | DBD | NoHep | DBD.F.NoHep_THEP15 | 26.88 |
| miR122 | DBD | NoHep | DBD.F.NoHep_THEP15 | 26.91 |
| miR148 | DBD | NoHep | DBD.F.NoHep_THEP15 | 28.79 |
| miR148 | DBD | NoHep | DBD.F.NoHep_THEP15 | 29.52 |
| miR148 | DBD | NoHep | DBD.F.NoHep_THEP15 | 29.99 |
| miR103 | DBD | NoHep | DBD.F.NoHep_THEP15 | 29.46 |
| miR103 | DBD | NoHep | DBD.F.NoHep_THEP15 | 29.48 |
| miR103 | DBD | NoHep | DBD.F.NoHep_THEP15 | 30.71 |
| miR191 | DBD | NoHep | DBD.F.NoHep_THEP15 | 34.78 |
| miR191 | DBD | NoHep | DBD.F.NoHep_THEP15 | 31.77 |
| miR191 | DBD | NoHep | DBD.F.NoHep_THEP15 | 31.73 |
| UNISP4 | DBD | NoHep | DBD.F.NoHep_THEP15 | 30.72 |
| UNISP4 | DBD | NoHep | DBD.F.NoHep_THEP15 | 30.37 |
| UNISP4 | DBD | NoHep | DBD.F.NoHep_THEP15 | 30.52 |
| miR39 | DBD | NoHep | DBD.F.NoHep_THEP15 | 24.91 |
| miR39 | DBD | NoHep | DBD.F.NoHep_THEP15 | 24.84 |
| miR39 | DBD | NoHep | DBD.F.NoHep_THEP15 | 24.85 |
| miR122 | DBD | NoHep | DBD.F.NoHep_THEP22 | 23.74 |
| miR122 | DBD | NoHep | DBD.F.NoHep_THEP22 | 23.72 |
| miR122 | DBD | NoHep | DBD.F.NoHep_THEP22 | 23.89 |
| miR148 | DBD | NoHep | DBD.F.NoHep_THEP22 | 26.65 |
| miR148 | DBD | NoHep | DBD.F.NoHep_THEP22 | 26.55 |
| miR148 | DBD | NoHep | DBD.F.NoHep_THEP22 | 26.45 |
| miR103 | DBD | NoHep | DBD.F.NoHep_THEP22 | 25.62 |
| miR103 | DBD | NoHep | DBD.F.NoHep_THEP22 | 25.57 |
| miR103 | DBD | NoHep | DBD.F.NoHep_THEP22 | 25.6 |
| miR191 | DBD | NoHep | DBD.F.NoHep_THEP22 | 26.69 |
| miR191 | DBD | NoHep | DBD.F.NoHep_THEP22 | 27.71 |
| miR191 | DBD | NoHep | DBD.F.NoHep_THEP22 | 27.69 |
| UNISP4 | DBD | NoHep | DBD.F.NoHep_THEP22 | 30.92 |
| UNISP4 | DBD | NoHep | DBD.F.NoHep_THEP22 | 30.7 |
| UNISP4 | DBD | NoHep | DBD.F.NoHep_THEP22 | 30.65 |
| miR39 | DBD | NoHep | DBD.F.NoHep_THEP22 | 24.85 |
| miR39 | DBD | NoHep | DBD.F.NoHep_THEP22 | 24.72 |
| miR39 | DBD | NoHep | DBD.F.NoHep_THEP22 | 24.48 |
| miR122 | DBD | NoHep | DBD.F.NoHep_THEP26 | 24.51 |
| miR122 | DBD | NoHep | DBD.F.NoHep_THEP26 | 24.35 |
| miR122 | DBD | NoHep | DBD.F.NoHep_THEP26 | 24.66 |
| miR148 | DBD | NoHep | DBD.F.NoHep_THEP26 | 27.46 |
| miR148 | DBD | NoHep | DBD.F.NoHep_THEP26 | 27.37 |
| miR148 | DBD | NoHep | DBD.F.NoHep_THEP26 | 27.18 |
| miR103 | DBD | NoHep | DBD.F.NoHep_THEP26 | 28.84 |
| miR103 | DBD | NoHep | DBD.F.NoHep_THEP26 | 29.22 |
| miR103 | DBD | NoHep | DBD.F.NoHep_THEP26 | 29.28 |
| miR191 | DBD | NoHep | DBD.F.NoHep_THEP26 | 29.85 |
| miR191 | DBD | NoHep | DBD.F.NoHep_THEP26 | 30.28 |
| miR191 | DBD | NoHep | DBD.F.NoHep_THEP26 | 30.42 |
| UNISP4 | DBD | NoHep | DBD.F.NoHep_THEP26 | 31.29 |
| UNISP4 | DBD | NoHep | DBD.F.NoHep_THEP26 | 30.75 |
| UNISP4 | DBD | NoHep | DBD.F.NoHep_THEP26 | 32.92 |
| miR39 | DBD | NoHep | DBD.F.NoHep_THEP26 | 24.57 |
| miR39 | DBD | NoHep | DBD.F.NoHep_THEP26 | 24.52 |
| miR39 | DBD | NoHep | DBD.F.NoHep_THEP26 | 24.64 |
| miR122 | DBD | NoHep | DBD.F.NoHep_THEP36 | 23.92 |
| miR122 | DBD | NoHep | DBD.F.NoHep_THEP36 | 23.97 |
| miR122 | DBD | NoHep | DBD.F.NoHep_THEP36 | 23.96 |
| miR148 | DBD | NoHep | DBD.F.NoHep_THEP36 | 27.14 |
| miR148 | DBD | NoHep | DBD.F.NoHep_THEP36 | 26.83 |
| miR148 | DBD | NoHep | DBD.F.NoHep_THEP36 | 26.83 |
| miR103 | DBD | NoHep | DBD.F.NoHep_THEP36 | 27.46 |
| miR103 | DBD | NoHep | DBD.F.NoHep_THEP36 | 27.43 |
| miR103 | DBD | NoHep | DBD.F.NoHep_THEP36 | 27.64 |
| miR191 | DBD | NoHep | DBD.F.NoHep_THEP36 | 29.93 |
| miR191 | DBD | NoHep | DBD.F.NoHep_THEP36 | 29.61 |
| miR191 | DBD | NoHep | DBD.F.NoHep_THEP36 | 29.94 |
| UNISP4 | DBD | NoHep | DBD.F.NoHep_THEP36 | 29.53 |
| UNISP4 | DBD | NoHep | DBD.F.NoHep_THEP36 | 29.48 |
| UNISP4 | DBD | NoHep | DBD.F.NoHep_THEP36 | 29.72 |
| miR39 | DBD | NoHep | DBD.F.NoHep_THEP36 | 24.62 |
| miR39 | DBD | NoHep | DBD.F.NoHep_THEP36 | 24.7 |
| miR39 | DBD | NoHep | DBD.F.NoHep_THEP36 | 24.56 |
| miR122 | DCD | Hep6U | DCD.F.Hep6U_THEP11 | 27.38 |
| miR122 | DCD | Hep6U | DCD.F.Hep6U_THEP11 | 27.34 |
| miR122 | DCD | Hep6U | DCD.F.Hep6U_THEP11 | 27.56 |
| miR148 | DCD | Hep6U | DCD.F.Hep6U_THEP11 | 30.37 |
| miR148 | DCD | Hep6U | DCD.F.Hep6U_THEP11 | 30.51 |
| miR148 | DCD | Hep6U | DCD.F.Hep6U_THEP11 | 29.59 |
| miR103 | DCD | Hep6U | DCD.F.Hep6U_THEP11 | 30.96 |
| miR103 | DCD | Hep6U | DCD.F.Hep6U_THEP11 | 30.77 |
| miR103 | DCD | Hep6U | DCD.F.Hep6U_THEP11 | 30.77 |
| miR191 | DCD | Hep6U | DCD.F.Hep6U_THEP11 |  |
| miR191 | DCD | Hep6U | DCD.F.Hep6U_THEP11 | 33.92 |
| miR191 | DCD | Hep6U | DCD.F.Hep6U_THEP11 | 34.15 |
| UNISP4 | DCD | Hep6U | DCD.F.Hep6U_THEP11 | 31.88 |
| UNISP4 | DCD | Hep6U | DCD.F.Hep6U_THEP11 | 32.41 |
| UNISP4 | DCD | Hep6U | DCD.F.Hep6U_THEP11 | 30.95 |
| miR39 | DCD | Hep6U | DCD.F.Hep6U_THEP11 | 24.81 |
| miR39 | DCD | Hep6U | DCD.F.Hep6U_THEP11 | 24.98 |
| miR39 | DCD | Hep6U | DCD.F.Hep6U_THEP11 | 24.81 |
| miR122 | DCD | Hep6U | DCD.F.Hep6U_THEP33 | 26.38 |
| miR122 | DCD | Hep6U | DCD.F.Hep6U_THEP33 | 26.57 |
| miR122 | DCD | Hep6U | DCD.F.Hep6U_THEP33 | 26.59 |
| miR148 | DCD | Hep6U | DCD.F.Hep6U_THEP33 | 29.65 |
| miR148 | DCD | Hep6U | DCD.F.Hep6U_THEP33 | 29.82 |
| miR148 | DCD | Hep6U | DCD.F.Hep6U_THEP33 | 28.81 |
| miR103 | DCD | Hep6U | DCD.F.Hep6U_THEP33 | 29.85 |
| miR103 | DCD | Hep6U | DCD.F.Hep6U_THEP33 | 28.9 |
| miR103 | DCD | Hep6U | DCD.F.Hep6U_THEP33 | 29.45 |
| miR191 | DCD | Hep6U | DCD.F.Hep6U_THEP33 | 30.69 |
| miR191 | DCD | Hep6U | DCD.F.Hep6U_THEP33 | 31.37 |
| miR191 | DCD | Hep6U | DCD.F.Hep6U_THEP33 | 31.31 |
| UNISP4 | DCD | Hep6U | DCD.F.Hep6U_THEP33 | 31.79 |
| UNISP4 | DCD | Hep6U | DCD.F.Hep6U_THEP33 | 32.44 |
| UNISP4 | DCD | Hep6U | DCD.F.Hep6U_THEP33 | 31.68 |
| miR39 | DCD | Hep6U | DCD.F.Hep6U_THEP33 | 24.84 |
| miR39 | DCD | Hep6U | DCD.F.Hep6U_THEP33 | 24.76 |
| miR39 | DCD | Hep6U | DCD.F.Hep6U_THEP33 | 24.52 |
| miR122 | DCD | Hep6U | DCD.F.Hep6U_THEP34 | 23.77 |
| miR122 | DCD | Hep6U | DCD.F.Hep6U_THEP34 | 23.87 |
| miR122 | DCD | Hep6U | DCD.F.Hep6U_THEP34 | 23.82 |
| miR148 | DCD | Hep6U | DCD.F.Hep6U_THEP34 | 26.48 |
| miR148 | DCD | Hep6U | DCD.F.Hep6U_THEP34 | 26.72 |
| miR148 | DCD | Hep6U | DCD.F.Hep6U_THEP34 | 26.83 |
| miR103 | DCD | Hep6U | DCD.F.Hep6U_THEP34 | 24.58 |
| miR103 | DCD | Hep6U | DCD.F.Hep6U_THEP34 | 24.59 |
| miR103 | DCD | Hep6U | DCD.F.Hep6U_THEP34 | 24.7 |
| miR191 | DCD | Hep6U | DCD.F.Hep6U_THEP34 | 26.84 |
| miR191 | DCD | Hep6U | DCD.F.Hep6U_THEP34 | 26.89 |
| miR191 | DCD | Hep6U | DCD.F.Hep6U_THEP34 | 27.23 |
| UNISP4 | DCD | Hep6U | DCD.F.Hep6U_THEP34 | 30.86 |
| UNISP4 | DCD | Hep6U | DCD.F.Hep6U_THEP34 | 31.82 |
| UNISP4 | DCD | Hep6U | DCD.F.Hep6U_THEP34 | 30.91 |
| miR39 | DCD | Hep6U | DCD.F.Hep6U_THEP34 | 24.8 |
| miR39 | DCD | Hep6U | DCD.F.Hep6U_THEP34 | 24.79 |
| miR39 | DCD | Hep6U | DCD.F.Hep6U_THEP34 | 24.62 |
| miR122 | DCD | Hep6U | DCD.F.Hep6U_THEP8 | 26.87 |
| miR122 | DCD | Hep6U | DCD.F.Hep6U_THEP8 | 26.67 |
| miR122 | DCD | Hep6U | DCD.F.Hep6U_THEP8 | 26.87 |
| miR148 | DCD | Hep6U | DCD.F.Hep6U_THEP8 | 29.59 |
| miR148 | DCD | Hep6U | DCD.F.Hep6U_THEP8 | 29.59 |
| miR148 | DCD | Hep6U | DCD.F.Hep6U_THEP8 | 29.54 |
| miR103 | DCD | Hep6U | DCD.F.Hep6U_THEP8 | 29.38 |
| miR103 | DCD | Hep6U | DCD.F.Hep6U_THEP8 | 29.41 |
| miR103 | DCD | Hep6U | DCD.F.Hep6U_THEP8 | 29.56 |
| miR191 | DCD | Hep6U | DCD.F.Hep6U_THEP8 | 31.22 |
| miR191 | DCD | Hep6U | DCD.F.Hep6U_THEP8 | 30.82 |
| miR191 | DCD | Hep6U | DCD.F.Hep6U_THEP8 | 31.57 |
| UNISP4 | DCD | Hep6U | DCD.F.Hep6U_THEP8 | 32.66 |
| UNISP4 | DCD | Hep6U | DCD.F.Hep6U_THEP8 | 32.37 |
| UNISP4 | DCD | Hep6U | DCD.F.Hep6U_THEP8 | 31.96 |
| miR39 | DCD | Hep6U | DCD.F.Hep6U_THEP8 | 25.23 |
| miR39 | DCD | Hep6U | DCD.F.Hep6U_THEP8 | 25.32 |
| miR39 | DCD | Hep6U | DCD.F.Hep6U_THEP8 | 25.38 |
| miR122 | DBD | Hep6U | DBD.F.Hep6U_THEP15 | 28.23 |
| miR122 | DBD | Hep6U | DBD.F.Hep6U_THEP15 | 28.33 |
| miR122 | DBD | Hep6U | DBD.F.Hep6U_THEP15 | 27.92 |
| miR148 | DBD | Hep6U | DBD.F.Hep6U_THEP15 | 31.94 |
| miR148 | DBD | Hep6U | DBD.F.Hep6U_THEP15 | 33.63 |
| miR148 | DBD | Hep6U | DBD.F.Hep6U_THEP15 | 30.99 |
| miR103 | DBD | Hep6U | DBD.F.Hep6U_THEP15 | 32.36 |
| miR103 | DBD | Hep6U | DBD.F.Hep6U_THEP15 | 30.58 |
| miR103 | DBD | Hep6U | DBD.F.Hep6U_THEP15 | 31.31 |
| miR191 | DBD | Hep6U | DBD.F.Hep6U_THEP15 | 35.57 |
| miR191 | DBD | Hep6U | DBD.F.Hep6U_THEP15 | 32.52 |
| miR191 | DBD | Hep6U | DBD.F.Hep6U_THEP15 |  |
| UNISP4 | DBD | Hep6U | DBD.F.Hep6U_THEP15 | 31.44 |
| UNISP4 | DBD | Hep6U | DBD.F.Hep6U_THEP15 | 31.5 |
| UNISP4 | DBD | Hep6U | DBD.F.Hep6U_THEP15 | 30.99 |
| miR39 | DBD | Hep6U | DBD.F.Hep6U_THEP15 | 24.87 |
| miR39 | DBD | Hep6U | DBD.F.Hep6U_THEP15 | 24.95 |
| miR39 | DBD | Hep6U | DBD.F.Hep6U_THEP15 | 24.87 |
| miR122 | DBD | Hep6U | DBD.F.Hep6U_THEP22 | 24.23 |
| miR122 | DBD | Hep6U | DBD.F.Hep6U_THEP22 | 24.31 |
| miR122 | DBD | Hep6U | DBD.F.Hep6U_THEP22 | 24.37 |
| miR148 | DBD | Hep6U | DBD.F.Hep6U_THEP22 | 27.43 |
| miR148 | DBD | Hep6U | DBD.F.Hep6U_THEP22 | 27.79 |
| miR148 | DBD | Hep6U | DBD.F.Hep6U_THEP22 | 27.55 |
| miR103 | DBD | Hep6U | DBD.F.Hep6U_THEP22 | 25.93 |
| miR103 | DBD | Hep6U | DBD.F.Hep6U_THEP22 | 26.32 |
| miR103 | DBD | Hep6U | DBD.F.Hep6U_THEP22 | 26.26 |
| miR191 | DBD | Hep6U | DBD.F.Hep6U_THEP22 | 27.45 |
| miR191 | DBD | Hep6U | DBD.F.Hep6U_THEP22 | 27.45 |
| miR191 | DBD | Hep6U | DBD.F.Hep6U_THEP22 | 27.19 |
| UNISP4 | DBD | Hep6U | DBD.F.Hep6U_THEP22 | 31.53 |
| UNISP4 | DBD | Hep6U | DBD.F.Hep6U_THEP22 | 30.62 |
| UNISP4 | DBD | Hep6U | DBD.F.Hep6U_THEP22 | 30.63 |
| miR39 | DBD | Hep6U | DBD.F.Hep6U_THEP22 | 24.81 |
| miR39 | DBD | Hep6U | DBD.F.Hep6U_THEP22 | 24.68 |
| miR39 | DBD | Hep6U | DBD.F.Hep6U_THEP22 | 24.79 |
| miR122 | DBD | Hep6U | DBD.F.Hep6U_THEP26 | 24.86 |
| miR122 | DBD | Hep6U | DBD.F.Hep6U_THEP26 | 24.87 |
| miR122 | DBD | Hep6U | DBD.F.Hep6U_THEP26 | 24.93 |
| miR148 | DBD | Hep6U | DBD.F.Hep6U_THEP26 | 27.88 |
| miR148 | DBD | Hep6U | DBD.F.Hep6U_THEP26 | 27.94 |
| miR148 | DBD | Hep6U | DBD.F.Hep6U_THEP26 | 28.87 |
| miR103 | DBD | Hep6U | DBD.F.Hep6U_THEP26 | 28.95 |
| miR103 | DBD | Hep6U | DBD.F.Hep6U_THEP26 | 29.43 |
| miR103 | DBD | Hep6U | DBD.F.Hep6U_THEP26 | 29.29 |
| miR191 | DBD | Hep6U | DBD.F.Hep6U_THEP26 | 32.77 |
| miR191 | DBD | Hep6U | DBD.F.Hep6U_THEP26 | 30.57 |
| miR191 | DBD | Hep6U | DBD.F.Hep6U_THEP26 | 29.98 |
| UNISP4 | DBD | Hep6U | DBD.F.Hep6U_THEP26 | 31.39 |
| UNISP4 | DBD | Hep6U | DBD.F.Hep6U_THEP26 | 30.59 |
| UNISP4 | DBD | Hep6U | DBD.F.Hep6U_THEP26 | 30.43 |
| miR39 | DBD | Hep6U | DBD.F.Hep6U_THEP26 | 24.72 |
| miR39 | DBD | Hep6U | DBD.F.Hep6U_THEP26 | 24.72 |
| miR39 | DBD | Hep6U | DBD.F.Hep6U_THEP26 | 24.69 |
| miR122 | DBD | Hep6U | DBD.F.Hep6U_THEP36 | 24.22 |
| miR122 | DBD | Hep6U | DBD.F.Hep6U_THEP36 | 24.3 |
| miR122 | DBD | Hep6U | DBD.F.Hep6U_THEP36 | 24.3 |
| miR148 | DBD | Hep6U | DBD.F.Hep6U_THEP36 | 26.9 |
| miR148 | DBD | Hep6U | DBD.F.Hep6U_THEP36 | 27 |
| miR148 | DBD | Hep6U | DBD.F.Hep6U_THEP36 | 27.37 |
| miR103 | DBD | Hep6U | DBD.F.Hep6U_THEP36 | 27.35 |
| miR103 | DBD | Hep6U | DBD.F.Hep6U_THEP36 | 27.49 |
| miR103 | DBD | Hep6U | DBD.F.Hep6U_THEP36 | 27.47 |
| miR191 | DBD | Hep6U | DBD.F.Hep6U_THEP36 | 29.23 |
| miR191 | DBD | Hep6U | DBD.F.Hep6U_THEP36 | 30.21 |
| miR191 | DBD | Hep6U | DBD.F.Hep6U_THEP36 | 29.45 |
| UNISP4 | DBD | Hep6U | DBD.F.Hep6U_THEP36 | 29.72 |
| UNISP4 | DBD | Hep6U | DBD.F.Hep6U_THEP36 | 29.77 |
| UNISP4 | DBD | Hep6U | DBD.F.Hep6U_THEP36 | 29.76 |
| miR39 | DBD | Hep6U | DBD.F.Hep6U_THEP36 | 24.48 |
| miR39 | DBD | Hep6U | DBD.F.Hep6U_THEP36 | 24.58 |
| miR39 | DBD | Hep6U | DBD.F.Hep6U_THEP36 | 24.49 |
| miR122 | DCD | Hep12U | DCD.F.Hep12U_THEP11 | 26.68 |
| miR122 | DCD | Hep12U | DCD.F.Hep12U_THEP11 | 26.82 |
| miR122 | DCD | Hep12U | DCD.F.Hep12U_THEP11 | 26.79 |
| miR148 | DCD | Hep12U | DCD.F.Hep12U_THEP11 | 29.73 |
| miR148 | DCD | Hep12U | DCD.F.Hep12U_THEP11 | 29.23 |
| miR148 | DCD | Hep12U | DCD.F.Hep12U_THEP11 | 29.25 |
| miR103 | DCD | Hep12U | DCD.F.Hep12U_THEP11 | 31.76 |
| miR103 | DCD | Hep12U | DCD.F.Hep12U_THEP11 | 31.51 |
| miR103 | DCD | Hep12U | DCD.F.Hep12U_THEP11 | 30.6 |
| miR191 | DCD | Hep12U | DCD.F.Hep12U_THEP11 |  |
| miR191 | DCD | Hep12U | DCD.F.Hep12U_THEP11 | 32.35 |
| miR191 | DCD | Hep12U | DCD.F.Hep12U_THEP11 | 32.99 |
| UNISP4 | DCD | Hep12U | DCD.F.Hep12U_THEP11 | 30.98 |
| UNISP4 | DCD | Hep12U | DCD.F.Hep12U_THEP11 | 32.47 |
| UNISP4 | DCD | Hep12U | DCD.F.Hep12U_THEP11 | 31.47 |
| miR39 | DCD | Hep12U | DCD.F.Hep12U_THEP11 | 24.81 |
| miR39 | DCD | Hep12U | DCD.F.Hep12U_THEP11 | 24.91 |
| miR39 | DCD | Hep12U | DCD.F.Hep12U_THEP11 | 24.93 |
| miR122 | DCD | Hep12U | DCD.F.Hep12U_THEP33 | 26.77 |
| miR122 | DCD | Hep12U | DCD.F.Hep12U_THEP33 | 26.73 |
| miR122 | DCD | Hep12U | DCD.F.Hep12U_THEP33 | 26.72 |
| miR148 | DCD | Hep12U | DCD.F.Hep12U_THEP33 | 29.56 |
| miR148 | DCD | Hep12U | DCD.F.Hep12U_THEP33 | 29.5 |
| miR148 | DCD | Hep12U | DCD.F.Hep12U_THEP33 | 30.59 |
| miR103 | DCD | Hep12U | DCD.F.Hep12U_THEP33 | 29.37 |
| miR103 | DCD | Hep12U | DCD.F.Hep12U_THEP33 | 29.64 |
| miR103 | DCD | Hep12U | DCD.F.Hep12U_THEP33 | 30.72 |
| miR191 | DCD | Hep12U | DCD.F.Hep12U_THEP33 | 32.39 |
| miR191 | DCD | Hep12U | DCD.F.Hep12U_THEP33 | 31.57 |
| miR191 | DCD | Hep12U | DCD.F.Hep12U_THEP33 | 33.77 |
| UNISP4 | DCD | Hep12U | DCD.F.Hep12U_THEP33 | 31.6 |
| UNISP4 | DCD | Hep12U | DCD.F.Hep12U_THEP33 | 31.63 |
| UNISP4 | DCD | Hep12U | DCD.F.Hep12U_THEP33 | 32.88 |
| miR39 | DCD | Hep12U | DCD.F.Hep12U_THEP33 | 24.45 |
| miR39 | DCD | Hep12U | DCD.F.Hep12U_THEP33 | 24.69 |
| miR39 | DCD | Hep12U | DCD.F.Hep12U_THEP33 | 24.58 |
| miR122 | DCD | Hep12U | DCD.F.Hep12U_THEP34 | 24.25 |
| miR122 | DCD | Hep12U | DCD.F.Hep12U_THEP34 | 23.71 |
| miR122 | DCD | Hep12U | DCD.F.Hep12U_THEP34 | 23.71 |
| miR148 | DCD | Hep12U | DCD.F.Hep12U_THEP34 | 26.77 |
| miR148 | DCD | Hep12U | DCD.F.Hep12U_THEP34 | 26.87 |
| miR148 | DCD | Hep12U | DCD.F.Hep12U_THEP34 | 27.33 |
| miR103 | DCD | Hep12U | DCD.F.Hep12U_THEP34 | 24.62 |
| miR103 | DCD | Hep12U | DCD.F.Hep12U_THEP34 | 24.51 |
| miR103 | DCD | Hep12U | DCD.F.Hep12U_THEP34 | 24.63 |
| miR191 | DCD | Hep12U | DCD.F.Hep12U_THEP34 | 24.91 |
| miR191 | DCD | Hep12U | DCD.F.Hep12U_THEP34 | 26.82 |
| miR191 | DCD | Hep12U | DCD.F.Hep12U_THEP34 | 26.71 |
| UNISP4 | DCD | Hep12U | DCD.F.Hep12U_THEP34 | 32.86 |
| UNISP4 | DCD | Hep12U | DCD.F.Hep12U_THEP34 | 31.69 |
| UNISP4 | DCD | Hep12U | DCD.F.Hep12U_THEP34 | 30.98 |
| miR39 | DCD | Hep12U | DCD.F.Hep12U_THEP34 | 24.88 |
| miR39 | DCD | Hep12U | DCD.F.Hep12U_THEP34 | 24.63 |
| miR39 | DCD | Hep12U | DCD.F.Hep12U_THEP34 | 24.61 |
| miR122 | DCD | Hep12U | DCD.F.Hep12U_THEP8 | 28.77 |
| miR122 | DCD | Hep12U | DCD.F.Hep12U_THEP8 | 28.56 |
| miR122 | DCD | Hep12U | DCD.F.Hep12U_THEP8 | 28.7 |
| miR148 | DCD | Hep12U | DCD.F.Hep12U_THEP8 | 32.61 |
| miR148 | DCD | Hep12U | DCD.F.Hep12U_THEP8 | 32.89 |
| miR148 | DCD | Hep12U | DCD.F.Hep12U_THEP8 | 31.42 |
| miR103 | DCD | Hep12U | DCD.F.Hep12U_THEP8 | 30.76 |
| miR103 | DCD | Hep12U | DCD.F.Hep12U_THEP8 | 30.72 |
| miR103 | DCD | Hep12U | DCD.F.Hep12U_THEP8 | 31.86 |
| miR191 | DCD | Hep12U | DCD.F.Hep12U_THEP8 | 32.34 |
| miR191 | DCD | Hep12U | DCD.F.Hep12U_THEP8 | 32.44 |
| miR191 | DCD | Hep12U | DCD.F.Hep12U_THEP8 | 33.47 |
| UNISP4 | DCD | Hep12U | DCD.F.Hep12U_THEP8 |  |
| UNISP4 | DCD | Hep12U | DCD.F.Hep12U_THEP8 |  |
| UNISP4 | DCD | Hep12U | DCD.F.Hep12U_THEP8 | 34.25 |
| miR39 | DCD | Hep12U | DCD.F.Hep12U_THEP8 | 24.88 |
| miR39 | DCD | Hep12U | DCD.F.Hep12U_THEP8 | 24.86 |
| miR39 | DCD | Hep12U | DCD.F.Hep12U_THEP8 | 25.33 |
| miR122 | DBD | Hep12U | DBD.F.Hep12U_THEP15 | 29.6 |
| miR122 | DBD | Hep12U | DBD.F.Hep12U_THEP15 | 29.27 |
| miR122 | DBD | Hep12U | DBD.F.Hep12U_THEP15 | 28.83 |
| miR148 | DBD | Hep12U | DBD.F.Hep12U_THEP15 |  |
| miR148 | DBD | Hep12U | DBD.F.Hep12U_THEP15 | 34.39 |
| miR148 | DBD | Hep12U | DBD.F.Hep12U_THEP15 |  |
| miR103 | DBD | Hep12U | DBD.F.Hep12U_THEP15 | 33.41 |
| miR103 | DBD | Hep12U | DBD.F.Hep12U_THEP15 |  |
| miR103 | DBD | Hep12U | DBD.F.Hep12U_THEP15 |  |
| miR191 | DBD | Hep12U | DBD.F.Hep12U_THEP15 |  |
| miR191 | DBD | Hep12U | DBD.F.Hep12U_THEP15 |  |
| miR191 | DBD | Hep12U | DBD.F.Hep12U_THEP15 |  |
| UNISP4 | DBD | Hep12U | DBD.F.Hep12U_THEP15 |  |
| UNISP4 | DBD | Hep12U | DBD.F.Hep12U_THEP15 |  |
| UNISP4 | DBD | Hep12U | DBD.F.Hep12U_THEP15 |  |
| miR39 | DBD | Hep12U | DBD.F.Hep12U_THEP15 | 24.99 |
| miR39 | DBD | Hep12U | DBD.F.Hep12U_THEP15 | 24.92 |
| miR39 | DBD | Hep12U | DBD.F.Hep12U_THEP15 | 24.78 |
| miR122 | DBD | Hep12U | DBD.F.Hep12U_THEP22 | 25.49 |
| miR122 | DBD | Hep12U | DBD.F.Hep12U_THEP22 | 25.27 |
| miR122 | DBD | Hep12U | DBD.F.Hep12U_THEP22 | 25.36 |
| miR148 | DBD | Hep12U | DBD.F.Hep12U_THEP22 | 29.33 |
| miR148 | DBD | Hep12U | DBD.F.Hep12U_THEP22 | 29 |
| miR148 | DBD | Hep12U | DBD.F.Hep12U_THEP22 | 28.46 |
| miR103 | DBD | Hep12U | DBD.F.Hep12U_THEP22 | 26.73 |
| miR103 | DBD | Hep12U | DBD.F.Hep12U_THEP22 | 27.32 |
| miR103 | DBD | Hep12U | DBD.F.Hep12U_THEP22 | 26.9 |
| miR191 | DBD | Hep12U | DBD.F.Hep12U_THEP22 | 27.98 |
| miR191 | DBD | Hep12U | DBD.F.Hep12U_THEP22 | 28.33 |
| miR191 | DBD | Hep12U | DBD.F.Hep12U_THEP22 | 28.58 |
| UNISP4 | DBD | Hep12U | DBD.F.Hep12U_THEP22 | 31.47 |
| UNISP4 | DBD | Hep12U | DBD.F.Hep12U_THEP22 | 32 |
| UNISP4 | DBD | Hep12U | DBD.F.Hep12U_THEP22 | 32.72 |
| miR39 | DBD | Hep12U | DBD.F.Hep12U_THEP22 | 24.84 |
| miR39 | DBD | Hep12U | DBD.F.Hep12U_THEP22 | 24.8 |
| miR39 | DBD | Hep12U | DBD.F.Hep12U_THEP22 | 24.78 |
| miR122 | DBD | Hep12U | DBD.F.Hep12U_THEP26 | 26.49 |
| miR122 | DBD | Hep12U | DBD.F.Hep12U_THEP26 | 26.54 |
| miR122 | DBD | Hep12U | DBD.F.Hep12U_THEP26 | 26.71 |
| miR148 | DBD | Hep12U | DBD.F.Hep12U_THEP26 | 30.86 |
| miR148 | DBD | Hep12U | DBD.F.Hep12U_THEP26 | 30.32 |
| miR148 | DBD | Hep12U | DBD.F.Hep12U_THEP26 | 30.52 |
| miR103 | DBD | Hep12U | DBD.F.Hep12U_THEP26 | 30.97 |
| miR103 | DBD | Hep12U | DBD.F.Hep12U_THEP26 | 31.78 |
| miR103 | DBD | Hep12U | DBD.F.Hep12U_THEP26 | 31.31 |
| miR191 | DBD | Hep12U | DBD.F.Hep12U_THEP26 | 31.47 |
| miR191 | DBD | Hep12U | DBD.F.Hep12U_THEP26 | 33.73 |
| miR191 | DBD | Hep12U | DBD.F.Hep12U_THEP26 | 31.75 |
| UNISP4 | DBD | Hep12U | DBD.F.Hep12U_THEP26 | 32.64 |
| UNISP4 | DBD | Hep12U | DBD.F.Hep12U_THEP26 |  |
| UNISP4 | DBD | Hep12U | DBD.F.Hep12U_THEP26 | 32.61 |
| miR39 | DBD | Hep12U | DBD.F.Hep12U_THEP26 | 24.9 |
| miR39 | DBD | Hep12U | DBD.F.Hep12U_THEP26 | 24.85 |
| miR39 | DBD | Hep12U | DBD.F.Hep12U_THEP26 | 24.85 |
| miR122 | DBD | Hep12U | DBD.F.Hep12U_THEP36 | 25.52 |
| miR122 | DBD | Hep12U | DBD.F.Hep12U_THEP36 | 25.62 |
| miR122 | DBD | Hep12U | DBD.F.Hep12U_THEP36 | 25.58 |
| miR148 | DBD | Hep12U | DBD.F.Hep12U_THEP36 | 28.56 |
| miR148 | DBD | Hep12U | DBD.F.Hep12U_THEP36 | 28.62 |
| miR148 | DBD | Hep12U | DBD.F.Hep12U_THEP36 | 28.61 |
| miR103 | DBD | Hep12U | DBD.F.Hep12U_THEP36 | 28.62 |
| miR103 | DBD | Hep12U | DBD.F.Hep12U_THEP36 | 29.26 |
| miR103 | DBD | Hep12U | DBD.F.Hep12U_THEP36 | 28.74 |
| miR191 | DBD | Hep12U | DBD.F.Hep12U_THEP36 | 31 |
| miR191 | DBD | Hep12U | DBD.F.Hep12U_THEP36 | 30.77 |
| miR191 | DBD | Hep12U | DBD.F.Hep12U_THEP36 | 31.31 |
| UNISP4 | DBD | Hep12U | DBD.F.Hep12U_THEP36 | 31.94 |
| UNISP4 | DBD | Hep12U | DBD.F.Hep12U_THEP36 | 32.56 |
| UNISP4 | DBD | Hep12U | DBD.F.Hep12U_THEP36 | 30.84 |
| miR39 | DBD | Hep12U | DBD.F.Hep12U_THEP36 | 24.66 |
| miR39 | DBD | Hep12U | DBD.F.Hep12U_THEP36 | 24.67 |
| miR39 | DBD | Hep12U | DBD.F.Hep12U_THEP36 | 24.81 |
