# Supplementary material for: Heparinase I treatment to overcome RNA quantification interference in heparinized liver donor samples: One size fits all?
Source: PLoS One. 2025 May 12;20(5):e0322899. doi: 10.1371/journal.pone.0322899 (PMC12068581; doi:10.1371/journal.pone.0322899)
Supplement: S3 Table — (DOCX) [file pone.0322899.s003.docx]

**S3 Table.** **miRNA Ct values in donor serum**.

(A) Global endogen miRNAs and Cel-miR-39 Ct means and standard deviations in eight liver donors. (B) Endogen miRNAs and Cel-miR-39 Ct means, and standard deviations divided in DBD (n=4) and paired samples of DCD-T0 and DCD-T1 donors (n=4). (C) Ct raw data triplicates in DBD and DCD_T0 serum without heparinase (NoHep), with 6 IU (Hep6U) and 12 IU (Hep12U). (D). Ct raw data triplicates in DBD, DCD_T0 and DCD_T1.

**A.**

| **miRNAs** | **n** | **Ct mean** | **Ct SD** |
| --- | --- | --- | --- |
| miR-122 | 12 | 31.47 | 2.15 |
| miR-148 | 12 | 31.91 | 1.70 |
| miR-103 | 12 | 32.42 | 1.64 |
| miR-191 | 12 | 30.62 | 1.83 |
| Cel-miR-39 | 12 | 28.03 | 0.30 |

**B.**

| **Donor group** | **miRNAs** | **n** | **Ct mean** | **Ct SD** |
| --- | --- | --- | --- | --- |
| DBD | miR-122 | 4 | 31.94 | 2.68 |
|  | miR-148a | 4 | 32.61 | 1.62 |
|  | miR-103a | 4 | 32.73 | 1.64 |
|  | miR-191 | 4 | 31.88 | 1.03 |
|  | Cel-miR-39 | 4 | 27.84 | 0.13 |
| DCD-T0 | miR-122 | 4 | 31.21 | 2.61 |
|  | miR-148a | 4 | 32.43 | 2.19 |
|  | miR-103a | 4 | 33.01 | 1.31 |
|  | miR-191 | 4 | 31.03 | 1.98 |
|  | Cel-miR-39 | 4 | 28.13 | 0.50 |
| DCD-T1 | miR-122 | 4 | 31.26 | 1.57 |
|  | miR-148a | 4 | 30.67 | 0.48 |
|  | miR-103a | 4 | 31.50 | 1.93 |
|  | miR-191 | 4 | 28.95 | 1.14 |
|  | Cel-miR-39 | 4 | 28.11 | 0.07 |

**C.**

| **miRNAs** | **Donor**  **Group** | **Treatment** | **Sample Name** | **Ct** |
| --- | --- | --- | --- | --- |
| mir39 | DCD_T0 | NoHep | DCD_T0.S.NoHep_THEP8 | 24.76 |
| mir103 | DCD_T0 | NoHep | DCD_T0.S.NoHep_THEP8 | 26.8 |
| mir148 | DCD_T0 | NoHep | DCD_T0.S.NoHep_THEP8 | 24.91 |
| mir191 | DCD_T0 | NoHep | DCD_T0.S.NoHep_THEP8 | 30.18 |
| mir103 | DCD_T0 | NoHep | DCD_T0.S.NoHep_THEP8 | 27.35 |
| mir148 | DCD_T0 | NoHep | DCD_T0.S.NoHep_THEP8 | 25.51 |
| mir103 | DCD_T0 | NoHep | DCD_T0.S.NoHep_THEP8 | 27.36 |
| UniSP4 | DCD_T0 | NoHep | DCD_T0.S.NoHep_THEP8 | 26.46 |
| mir39 | DCD_T0 | NoHep | DCD_T0.S.NoHep_THEP8 | 24.92 |
| mir148 | DCD_T0 | NoHep | DCD_T0.S.NoHep_THEP8 | 25.53 |
| UniSP4 | DCD_T0 | NoHep | DCD_T0.S.NoHep_THEP8 | 26.42 |
| mir39 | DCD_T0 | NoHep | DCD_T0.S.NoHep_THEP8 | 24.65 |
| mir122 | DCD_T0 | NoHep | DCD_T0.S.NoHep_THEP8 | 24.61 |
| mir191 | DCD_T0 | NoHep | DCD_T0.S.NoHep_THEP8 | 29.72 |
| UniSP4 | DCD_T0 | NoHep | DCD_T0.S.NoHep_THEP8 | 26.56 |
| mir122 | DCD_T0 | NoHep | DCD_T0.S.NoHep_THEP8 | 24.76 |
| mir191 | DCD_T0 | NoHep | DCD_T0.S.NoHep_THEP8 | 29.56 |
| mir122 | DCD_T0 | NoHep | DCD_T0.S.NoHep_THEP8 | 24.59 |
| mir148 | DCD_T0 | Hep6U | DCD_T0.S.Hep6U_THEP8 |  |
| mir39 | DCD_T0 | Hep6U | DCD_T0.S.Hep6U_THEP8 | 24.9 |
| mir148 | DCD_T0 | Hep6U | DCD_T0.S.Hep6U_THEP8 |  |
| mir39 | DCD_T0 | Hep6U | DCD_T0.S.Hep6U_THEP8 | 24.51 |
| mir39 | DCD_T0 | Hep6U | DCD_T0.S.Hep6U_THEP8 | 24.87 |
| mir103 | DCD_T0 | Hep6U | DCD_T0.S.Hep6U_THEP8 |  |
| mir122 | DCD_T0 | Hep6U | DCD_T0.S.Hep6U_THEP8 | 32.37 |
| mir191 | DCD_T0 | Hep6U | DCD_T0.S.Hep6U_THEP8 |  |
| UniSP4 | DCD_T0 | Hep6U | DCD_T0.S.Hep6U_THEP8 |  |
| mir103 | DCD_T0 | Hep6U | DCD_T0.S.Hep6U_THEP8 |  |
| mir122 | DCD_T0 | Hep6U | DCD_T0.S.Hep6U_THEP8 | 33.28 |
| mir103 | DCD_T0 | Hep6U | DCD_T0.S.Hep6U_THEP8 |  |
| mir191 | DCD_T0 | Hep6U | DCD_T0.S.Hep6U_THEP8 | 35.25 |
| mir122 | DCD_T0 | Hep6U | DCD_T0.S.Hep6U_THEP8 | 32.45 |
| UniSP4 | DCD_T0 | Hep6U | DCD_T0.S.Hep6U_THEP8 |  |
| mir191 | DCD_T0 | Hep6U | DCD_T0.S.Hep6U_THEP8 |  |
| mir148 | DCD_T0 | Hep6U | DCD_T0.S.Hep6U_THEP8 |  |
| UniSP4 | DCD_T0 | Hep6U | DCD_T0.S.Hep6U_THEP8 | 36.94 |
| mir191 | DCD_T0 | Hep12U | DCD_T0.S.Hep12U_THEP8 |  |
| mir148 | DCD_T0 | Hep12U | DCD_T0.S.Hep12U_THEP8 |  |
| mir122 | DCD_T0 | Hep12U | DCD_T0.S.Hep12U_THEP8 | 32,00 |
| UniSP4 | DCD_T0 | Hep12U | DCD_T0.S.Hep12U_THEP8 |  |
| mir122 | DCD_T0 | Hep12U | DCD_T0.S.Hep12U_THEP8 | 30.86 |
| UniSP4 | DCD_T0 | Hep12U | DCD_T0.S.Hep12U_THEP8 |  |
| mir103 | DCD_T0 | Hep12U | DCD_T0.S.Hep12U_THEP8 |  |
| mir122 | DCD_T0 | Hep12U | DCD_T0.S.Hep12U_THEP8 | 32.38 |
| mir148 | DCD_T0 | Hep12U | DCD_T0.S.Hep12U_THEP8 |  |
| mir148 | DCD_T0 | Hep12U | DCD_T0.S.Hep12U_THEP8 |  |
| UniSP4 | DCD_T0 | Hep12U | DCD_T0.S.Hep12U_THEP8 |  |
| mir103 | DCD_T0 | Hep12U | DCD_T0.S.Hep12U_THEP8 |  |
| mir39 | DCD_T0 | Hep12U | DCD_T0.S.Hep12U_THEP8 | 24.76 |
| mir39 | DCD_T0 | Hep12U | DCD_T0.S.Hep12U_THEP8 | 24.76 |
| mir39 | DCD_T0 | Hep12U | DCD_T0.S.Hep12U_THEP8 | 24.78 |
| mir103 | DCD_T0 | Hep12U | DCD_T0.S.Hep12U_THEP8 | 37.41 |
| mir191 | DCD_T0 | Hep12U | DCD_T0.S.Hep12U_THEP8 |  |
| mir191 | DCD_T0 | Hep12U | DCD_T0.S.Hep12U_THEP8 |  |
| mir39 | DCD_T0 | NoHep | DCD_T0.S.NoHep_THEP11 | 25.84 |
| UniSP4 | DCD_T0 | NoHep | DCD_T0.S.NoHep_THEP11 | 29.39 |
| mir103 | DCD_T0 | NoHep | DCD_T0.S.NoHep_THEP11 | 26.61 |
| mir148 | DCD_T0 | NoHep | DCD_T0.S.NoHep_THEP11 | 25.79 |
| mir122 | DCD_T0 | NoHep | DCD_T0.S.NoHep_THEP11 | 29.72 |
| mir122 | DCD_T0 | NoHep | DCD_T0.S.NoHep_THEP11 | 30.25 |
| mir39 | DCD_T0 | NoHep | DCD_T0.S.NoHep_THEP11 | 25.88 |
| mir39 | DCD_T0 | NoHep | DCD_T0.S.NoHep_THEP11 | 25.98 |
| mir148 | DCD_T0 | NoHep | DCD_T0.S.NoHep_THEP11 | 25.71 |
| mir191 | DCD_T0 | NoHep | DCD_T0.S.NoHep_THEP11 | 26.18 |
| UniSP4 | DCD_T0 | NoHep | DCD_T0.S.NoHep_THEP11 | 29.39 |
| mir191 | DCD_T0 | NoHep | DCD_T0.S.NoHep_THEP11 | 25.95 |
| mir191 | DCD_T0 | NoHep | DCD_T0.S.NoHep_THEP11 | 26.17 |
| mir103 | DCD_T0 | NoHep | DCD_T0.S.NoHep_THEP11 | 26.93 |
| mir148 | DCD_T0 | NoHep | DCD_T0.S.NoHep_THEP11 | 25.78 |
| UniSP4 | DCD_T0 | NoHep | DCD_T0.S.NoHep_THEP11 | 29,00 |
| mir122 | DCD_T0 | NoHep | DCD_T0.S.NoHep_THEP11 | 29.62 |
| mir103 | DCD_T0 | NoHep | DCD_T0.S.NoHep_THEP11 | 26.82 |
| mir191 | DCD_T0 | Hep6U | DCD_T0.S.Hep6U_THEP11 | 30.93 |
| mir103 | DCD_T0 | Hep6U | DCD_T0.S.Hep6U_THEP11 | 30.6 |
| mir148 | DCD_T0 | Hep6U | DCD_T0.S.Hep6U_THEP11 | 31.74 |
| mir148 | DCD_T0 | Hep6U | DCD_T0.S.Hep6U_THEP11 | 33.33 |
| mir39 | DCD_T0 | Hep6U | DCD_T0.S.Hep6U_THEP11 | 24.58 |
| mir148 | DCD_T0 | Hep6U | DCD_T0.S.Hep6U_THEP11 | 32.63 |
| mir39 | DCD_T0 | Hep6U | DCD_T0.S.Hep6U_THEP11 | 24.65 |
| mir103 | DCD_T0 | Hep6U | DCD_T0.S.Hep6U_THEP11 | 30.32 |
| UniSP4 | DCD_T0 | Hep6U | DCD_T0.S.Hep6U_THEP11 | 34.64 |
| UniSP4 | DCD_T0 | Hep6U | DCD_T0.S.Hep6U_THEP11 | 34.9 |
| mir122 | DCD_T0 | Hep6U | DCD_T0.S.Hep6U_THEP11 | 33.56 |
| UniSP4 | DCD_T0 | Hep6U | DCD_T0.S.Hep6U_THEP11 | 33.9 |
| mir191 | DCD_T0 | Hep6U | DCD_T0.S.Hep6U_THEP11 | 30.86 |
| mir122 | DCD_T0 | Hep6U | DCD_T0.S.Hep6U_THEP11 | 32.39 |
| mir39 | DCD_T0 | Hep6U | DCD_T0.S.Hep6U_THEP11 | 24.49 |
| mir122 | DCD_T0 | Hep6U | DCD_T0.S.Hep6U_THEP11 |  |
| mir191 | DCD_T0 | Hep6U | DCD_T0.S.Hep6U_THEP11 | 30.91 |
| mir103 | DCD_T0 | Hep6U | DCD_T0.S.Hep6U_THEP11 | 29.73 |
| mir103 | DCD_T0 | Hep12U | DCD_T0.S.Hep12U_THEP11 |  |
| mir39 | DCD_T0 | Hep12U | DCD_T0.S.Hep12U_THEP11 | 24.84 |
| UniSP4 | DCD_T0 | Hep12U | DCD_T0.S.Hep12U_THEP11 |  |
| mir39 | DCD_T0 | Hep12U | DCD_T0.S.Hep12U_THEP11 | 24.64 |
| mir148 | DCD_T0 | Hep12U | DCD_T0.S.Hep12U_THEP11 |  |
| UniSP4 | DCD_T0 | Hep12U | DCD_T0.S.Hep12U_THEP11 |  |
| mir122 | DCD_T0 | Hep12U | DCD_T0.S.Hep12U_THEP11 |  |
| mir148 | DCD_T0 | Hep12U | DCD_T0.S.Hep12U_THEP11 |  |
| mir39 | DCD_T0 | Hep12U | DCD_T0.S.Hep12U_THEP11 | 24.56 |
| mir191 | DCD_T0 | Hep12U | DCD_T0.S.Hep12U_THEP11 | 34.85 |
| mir122 | DCD_T0 | Hep12U | DCD_T0.S.Hep12U_THEP11 |  |
| mir148 | DCD_T0 | Hep12U | DCD_T0.S.Hep12U_THEP11 |  |
| mir191 | DCD_T0 | Hep12U | DCD_T0.S.Hep12U_THEP11 |  |
| mir191 | DCD_T0 | Hep12U | DCD_T0.S.Hep12U_THEP11 | 35.72 |
| mir103 | DCD_T0 | Hep12U | DCD_T0.S.Hep12U_THEP11 |  |
| mir103 | DCD_T0 | Hep12U | DCD_T0.S.Hep12U_THEP11 |  |
| UniSP4 | DCD_T0 | Hep12U | DCD_T0.S.Hep12U_THEP11 |  |
| mir122 | DCD_T0 | Hep12U | DCD_T0.S.Hep12U_THEP11 |  |
| mir39 | DCD_T0 | NoHep | DCD_T0.S.NoHep_THEP33 | 24.73 |
| mir103 | DCD_T0 | NoHep | DCD_T0.S.NoHep_THEP33 | 26.73 |
| mir39 | DCD_T0 | NoHep | DCD_T0.S.NoHep_THEP33 | 25.75 |
| mir191 | DCD_T0 | NoHep | DCD_T0.S.NoHep_THEP33 | 28.78 |
| mir122 | DCD_T0 | NoHep | DCD_T0.S.NoHep_THEP33 | 27.97 |
| UniSP4 | DCD_T0 | NoHep | DCD_T0.S.NoHep_THEP33 | 26.5 |
| UniSP4 | DCD_T0 | NoHep | DCD_T0.S.NoHep_THEP33 | 26.57 |
| mir103 | DCD_T0 | NoHep | DCD_T0.S.NoHep_THEP33 | 27.44 |
| mir191 | DCD_T0 | NoHep | DCD_T0.S.NoHep_THEP33 | 29.39 |
| mir122 | DCD_T0 | NoHep | DCD_T0.S.NoHep_THEP33 | 28.65 |
| UniSP4 | DCD_T0 | NoHep | DCD_T0.S.NoHep_THEP33 | 26.65 |
| mir103 | DCD_T0 | NoHep | DCD_T0.S.NoHep_THEP33 | 27.48 |
| mir191 | DCD_T0 | NoHep | DCD_T0.S.NoHep_THEP33 | 29.41 |
| mir39 | DCD_T0 | NoHep | DCD_T0.S.NoHep_THEP33 | 24.74 |
| mir122 | DCD_T0 | NoHep | DCD_T0.S.NoHep_THEP33 | 28.34 |
| mir148 | DCD_T0 | NoHep | DCD_T0.S.NoHep_THEP33 | 26.74 |
| mir148 | DCD_T0 | NoHep | DCD_T0.S.NoHep_THEP33 | 27.6 |
| mir148 | DCD_T0 | NoHep | DCD_T0.S.NoHep_THEP33 | 26.66 |
| mir191 | DCD_T0 | Hep6U | DCD_T0.S.Hep6U_THEP33 |  |
| mir103 | DCD_T0 | Hep6U | DCD_T0.S.Hep6U_THEP33 | 33.32 |
| mir122 | DCD_T0 | Hep6U | DCD_T0.S.Hep6U_THEP33 |  |
| mir103 | DCD_T0 | Hep6U | DCD_T0.S.Hep6U_THEP33 | 32.82 |
| mir103 | DCD_T0 | Hep6U | DCD_T0.S.Hep6U_THEP33 |  |
| mir148 | DCD_T0 | Hep6U | DCD_T0.S.Hep6U_THEP33 |  |
| mir148 | DCD_T0 | Hep6U | DCD_T0.S.Hep6U_THEP33 |  |
| UniSP4 | DCD_T0 | Hep6U | DCD_T0.S.Hep6U_THEP33 | 36.48 |
| mir191 | DCD_T0 | Hep6U | DCD_T0.S.Hep6U_THEP33 |  |
| UniSP4 | DCD_T0 | Hep6U | DCD_T0.S.Hep6U_THEP33 | 34.84 |
| mir39 | DCD_T0 | Hep6U | DCD_T0.S.Hep6U_THEP33 | 24.48 |
| UniSP4 | DCD_T0 | Hep6U | DCD_T0.S.Hep6U_THEP33 |  |
| mir122 | DCD_T0 | Hep6U | DCD_T0.S.Hep6U_THEP33 |  |
| mir39 | DCD_T0 | Hep6U | DCD_T0.S.Hep6U_THEP33 | 24.56 |
| mir39 | DCD_T0 | Hep6U | DCD_T0.S.Hep6U_THEP33 | 24.68 |
| mir148 | DCD_T0 | Hep6U | DCD_T0.S.Hep6U_THEP33 |  |
| mir191 | DCD_T0 | Hep6U | DCD_T0.S.Hep6U_THEP33 |  |
| mir122 | DCD_T0 | Hep6U | DCD_T0.S.Hep6U_THEP33 |  |
| UniSP4 | DCD_T0 | Hep12U | DCD_T0.S.Hep12U_THEP33 |  |
| mir103 | DCD_T0 | Hep12U | DCD_T0.S.Hep12U_THEP33 |  |
| mir122 | DCD_T0 | Hep12U | DCD_T0.S.Hep12U_THEP33 |  |
| UniSP4 | DCD_T0 | Hep12U | DCD_T0.S.Hep12U_THEP33 |  |
| mir191 | DCD_T0 | Hep12U | DCD_T0.S.Hep12U_THEP33 |  |
| UniSP4 | DCD_T0 | Hep12U | DCD_T0.S.Hep12U_THEP33 |  |
| mir122 | DCD_T0 | Hep12U | DCD_T0.S.Hep12U_THEP33 |  |
| mir39 | DCD_T0 | Hep12U | DCD_T0.S.Hep12U_THEP33 | 24.49 |
| mir39 | DCD_T0 | Hep12U | DCD_T0.S.Hep12U_THEP33 | 24.57 |
| mir39 | DCD_T0 | Hep12U | DCD_T0.S.Hep12U_THEP33 | 24.59 |
| mir148 | DCD_T0 | Hep12U | DCD_T0.S.Hep12U_THEP33 |  |
| mir148 | DCD_T0 | Hep12U | DCD_T0.S.Hep12U_THEP33 |  |
| mir122 | DCD_T0 | Hep12U | DCD_T0.S.Hep12U_THEP33 |  |
| mir103 | DCD_T0 | Hep12U | DCD_T0.S.Hep12U_THEP33 |  |
| mir191 | DCD_T0 | Hep12U | DCD_T0.S.Hep12U_THEP33 |  |
| mir148 | DCD_T0 | Hep12U | DCD_T0.S.Hep12U_THEP33 |  |
| mir191 | DCD_T0 | Hep12U | DCD_T0.S.Hep12U_THEP33 | 34.93 |
| mir103 | DCD_T0 | Hep12U | DCD_T0.S.Hep12U_THEP33 |  |
| mir39 | DCD_T0 | NoHep | DCD_T0.S.NoHep_THEP34 | 24.57 |
| mir103 | DCD_T0 | NoHep | DCD_T0.S.NoHep_THEP34 | 28.53 |
| mir191 | DCD_T0 | NoHep | DCD_T0.S.NoHep_THEP34 | 30.58 |
| mir122 | DCD_T0 | NoHep | DCD_T0.S.NoHep_THEP34 | 27.88 |
| mir39 | DCD_T0 | NoHep | DCD_T0.S.NoHep_THEP34 | 24.59 |
| mir191 | DCD_T0 | NoHep | DCD_T0.S.NoHep_THEP34 | 30.28 |
| mir148 | DCD_T0 | NoHep | DCD_T0.S.NoHep_THEP34 | 28.62 |
| mir39 | DCD_T0 | NoHep | DCD_T0.S.NoHep_THEP34 | 23.69 |
| mir148 | DCD_T0 | NoHep | DCD_T0.S.NoHep_THEP34 | 29.47 |
| mir103 | DCD_T0 | NoHep | DCD_T0.S.NoHep_THEP34 | 29.44 |
| mir148 | DCD_T0 | NoHep | DCD_T0.S.NoHep_THEP34 | 30.52 |
| UniSP4 | DCD_T0 | NoHep | DCD_T0.S.NoHep_THEP34 | 27.39 |
| mir122 | DCD_T0 | NoHep | DCD_T0.S.NoHep_THEP34 | 28.56 |
| mir103 | DCD_T0 | NoHep | DCD_T0.S.NoHep_THEP34 | 29.35 |
| mir122 | DCD_T0 | NoHep | DCD_T0.S.NoHep_THEP34 | 28.52 |
| mir191 | DCD_T0 | NoHep | DCD_T0.S.NoHep_THEP34 | 29.99 |
| UniSP4 | DCD_T0 | NoHep | DCD_T0.S.NoHep_THEP34 | 27.36 |
| UniSP4 | DCD_T0 | NoHep | DCD_T0.S.NoHep_THEP34 | 27.42 |
| mir39 | DCD_T0 | Hep6U | DCD_T0.S.Hep6U_THEP34 | 24.68 |
| mir103 | DCD_T0 | Hep6U | DCD_T0.S.Hep6U_THEP34 |  |
| mir191 | DCD_T0 | Hep6U | DCD_T0.S.Hep6U_THEP34 |  |
| mir122 | DCD_T0 | Hep6U | DCD_T0.S.Hep6U_THEP34 |  |
| UniSP4 | DCD_T0 | Hep6U | DCD_T0.S.Hep6U_THEP34 | 36.28 |
| UniSP4 | DCD_T0 | Hep6U | DCD_T0.S.Hep6U_THEP34 |  |
| mir148 | DCD_T0 | Hep6U | DCD_T0.S.Hep6U_THEP34 |  |
| mir191 | DCD_T0 | Hep6U | DCD_T0.S.Hep6U_THEP34 |  |
| UniSP4 | DCD_T0 | Hep6U | DCD_T0.S.Hep6U_THEP34 |  |
| mir148 | DCD_T0 | Hep6U | DCD_T0.S.Hep6U_THEP34 |  |
| mir103 | DCD_T0 | Hep6U | DCD_T0.S.Hep6U_THEP34 |  |
| mir191 | DCD_T0 | Hep6U | DCD_T0.S.Hep6U_THEP34 |  |
| mir122 | DCD_T0 | Hep6U | DCD_T0.S.Hep6U_THEP34 |  |
| mir39 | DCD_T0 | Hep6U | DCD_T0.S.Hep6U_THEP34 | 24.68 |
| mir122 | DCD_T0 | Hep6U | DCD_T0.S.Hep6U_THEP34 |  |
| mir103 | DCD_T0 | Hep6U | DCD_T0.S.Hep6U_THEP34 |  |
| mir148 | DCD_T0 | Hep6U | DCD_T0.S.Hep6U_THEP34 |  |
| mir39 | DCD_T0 | Hep6U | DCD_T0.S.Hep6U_THEP34 | 24.77 |
| UniSP4 | DCD_T0 | Hep12U | DCD_T0.S.Hep12U_THEP34 |  |
| mir103 | DCD_T0 | Hep12U | DCD_T0.S.Hep12U_THEP34 |  |
| mir191 | DCD_T0 | Hep12U | DCD_T0.S.Hep12U_THEP34 |  |
| mir191 | DCD_T0 | Hep12U | DCD_T0.S.Hep12U_THEP34 |  |
| mir39 | DCD_T0 | Hep12U | DCD_T0.S.Hep12U_THEP34 | 24.7 |
| mir148 | DCD_T0 | Hep12U | DCD_T0.S.Hep12U_THEP34 | 37.52 |
| mir103 | DCD_T0 | Hep12U | DCD_T0.S.Hep12U_THEP34 |  |
| mir103 | DCD_T0 | Hep12U | DCD_T0.S.Hep12U_THEP34 |  |
| mir122 | DCD_T0 | Hep12U | DCD_T0.S.Hep12U_THEP34 |  |
| mir39 | DCD_T0 | Hep12U | DCD_T0.S.Hep12U_THEP34 | 24.84 |
| UniSP4 | DCD_T0 | Hep12U | DCD_T0.S.Hep12U_THEP34 |  |
| mir148 | DCD_T0 | Hep12U | DCD_T0.S.Hep12U_THEP34 |  |
| mir148 | DCD_T0 | Hep12U | DCD_T0.S.Hep12U_THEP34 |  |
| mir39 | DCD_T0 | Hep12U | DCD_T0.S.Hep12U_THEP34 | 24.72 |
| UniSP4 | DCD_T0 | Hep12U | DCD_T0.S.Hep12U_THEP34 | 41.45 |
| mir122 | DCD_T0 | Hep12U | DCD_T0.S.Hep12U_THEP34 |  |
| mir122 | DCD_T0 | Hep12U | DCD_T0.S.Hep12U_THEP34 | 33.39 |
| mir191 | DCD_T0 | Hep12U | DCD_T0.S.Hep12U_THEP34 |  |
| mir39 | DBD_T0 | NoHep | DBD_T0.S.NoHep_THEP15 | 24.67 |
| mir191 | DBD_T0 | NoHep | DBD_T0.S.NoHep_THEP15 | 29.92 |
| mir148 | DBD_T0 | NoHep | DBD_T0.S.NoHep_THEP15 | 28.51 |
| mir39 | DBD_T0 | NoHep | DBD_T0.S.NoHep_THEP15 | 24.75 |
| UniSP4 | DBD_T0 | NoHep | DBD_T0.S.NoHep_THEP15 | 25.51 |
| mir103 | DBD_T0 | NoHep | DBD_T0.S.NoHep_THEP15 | 26.59 |
| mir148 | DBD_T0 | NoHep | DBD_T0.S.NoHep_THEP15 | 27.99 |
| mir122 | DBD_T0 | NoHep | DBD_T0.S.NoHep_THEP15 | 29.43 |
| UniSP4 | DBD_T0 | NoHep | DBD_T0.S.NoHep_THEP15 | 25.43 |
| UniSP4 | DBD_T0 | NoHep | DBD_T0.S.NoHep_THEP15 | 25.48 |
| mir191 | DBD_T0 | NoHep | DBD_T0.S.NoHep_THEP15 | 29.49 |
| mir122 | DBD_T0 | NoHep | DBD_T0.S.NoHep_THEP15 | 28.98 |
| mir39 | DBD_T0 | NoHep | DBD_T0.S.NoHep_THEP15 | 24.65 |
| mir148 | DBD_T0 | NoHep | DBD_T0.S.NoHep_THEP15 | 27.81 |
| mir103 | DBD_T0 | NoHep | DBD_T0.S.NoHep_THEP15 | 26.83 |
| mir122 | DBD_T0 | NoHep | DBD_T0.S.NoHep_THEP15 | 29.48 |
| mir103 | DBD_T0 | NoHep | DBD_T0.S.NoHep_THEP15 | 26.73 |
| mir191 | DBD_T0 | NoHep | DBD_T0.S.NoHep_THEP15 | 29.88 |
| mir103 | DBD_T0 | Hep6U | DBD_T0.S.Hep6U_THEP15 |  |
| UniSP4 | DBD_T0 | Hep6U | DBD_T0.S.Hep6U_THEP15 |  |
| mir39 | DBD_T0 | Hep6U | DBD_T0.S.Hep6U_THEP15 | 24.62 |
| mir122 | DBD_T0 | Hep6U | DBD_T0.S.Hep6U_THEP15 |  |
| mir39 | DBD_T0 | Hep6U | DBD_T0.S.Hep6U_THEP15 | 24.56 |
| mir39 | DBD_T0 | Hep6U | DBD_T0.S.Hep6U_THEP15 | 24.58 |
| mir191 | DBD_T0 | Hep6U | DBD_T0.S.Hep6U_THEP15 |  |
| mir148 | DBD_T0 | Hep6U | DBD_T0.S.Hep6U_THEP15 |  |
| mir148 | DBD_T0 | Hep6U | DBD_T0.S.Hep6U_THEP15 | 34.8 |
| mir122 | DBD_T0 | Hep6U | DBD_T0.S.Hep6U_THEP15 |  |
| mir191 | DBD_T0 | Hep6U | DBD_T0.S.Hep6U_THEP15 | 34.84 |
| mir122 | DBD_T0 | Hep6U | DBD_T0.S.Hep6U_THEP15 |  |
| mir103 | DBD_T0 | Hep6U | DBD_T0.S.Hep6U_THEP15 |  |
| mir191 | DBD_T0 | Hep6U | DBD_T0.S.Hep6U_THEP15 |  |
| UniSP4 | DBD_T0 | Hep6U | DBD_T0.S.Hep6U_THEP15 | 34.72 |
| mir103 | DBD_T0 | Hep6U | DBD_T0.S.Hep6U_THEP15 |  |
| UniSP4 | DBD_T0 | Hep6U | DBD_T0.S.Hep6U_THEP15 |  |
| mir148 | DBD_T0 | Hep6U | DBD_T0.S.Hep6U_THEP15 | 40.7 |
| mir122 | DBD_T0 | Hep12U | DBD_T0.S.Hep12_THEP15 |  |
| mir39 | DBD_T0 | Hep12U | DBD_T0.S.Hep12_THEP15 | 24.66 |
| mir39 | DBD_T0 | Hep12U | DBD_T0.S.Hep12_THEP15 | 24.61 |
| UniSP4 | DBD_T0 | Hep12U | DBD_T0.S.Hep12_THEP15 |  |
| mir148 | DBD_T0 | Hep12U | DBD_T0.S.Hep12_THEP15 |  |
| mir191 | DBD_T0 | Hep12U | DBD_T0.S.Hep12_THEP15 | 40.2 |
| mir191 | DBD_T0 | Hep12U | DBD_T0.S.Hep12_THEP15 | 35,00 |
| mir122 | DBD_T0 | Hep12U | DBD_T0.S.Hep12_THEP15 |  |
| mir148 | DBD_T0 | Hep12U | DBD_T0.S.Hep12_THEP15 |  |
| mir103 | DBD_T0 | Hep12U | DBD_T0.S.Hep12_THEP15 |  |
| UniSP4 | DBD_T0 | Hep12U | DBD_T0.S.Hep12_THEP15 | 34.91 |
| mir148 | DBD_T0 | Hep12U | DBD_T0.S.Hep12_THEP15 | 32.97 |
| mir103 | DBD_T0 | Hep12U | DBD_T0.S.Hep12_THEP15 |  |
| mir103 | DBD_T0 | Hep12U | DBD_T0.S.Hep12_THEP15 |  |
| mir39 | DBD_T0 | Hep12U | DBD_T0.S.Hep12_THEP15 | 24.6 |
| mir122 | DBD_T0 | Hep12U | DBD_T0.S.Hep12_THEP15 | 33.47 |
| UniSP4 | DBD_T0 | Hep12U | DBD_T0.S.Hep12_THEP15 |  |
| mir191 | DBD_T0 | Hep12U | DBD_T0.S.Hep12_THEP15 |  |
| mir148 | DBD_T0 | NoHep | DBD_T0.S.NoHep_THEP22 | 25.7 |
| mir191 | DBD_T0 | NoHep | DBD_T0.S.NoHep_THEP22 | 28.21 |
| mir39 | DBD_T0 | NoHep | DBD_T0.S.NoHep_THEP22 | 24.62 |
| mir122 | DBD_T0 | NoHep | DBD_T0.S.NoHep_THEP22 | 27.79 |
| UniSP4 | DBD_T0 | NoHep | DBD_T0.S.NoHep_THEP22 | 24.42 |
| mir103 | DBD_T0 | NoHep | DBD_T0.S.NoHep_THEP22 | 24.76 |
| mir39 | DBD_T0 | NoHep | DBD_T0.S.NoHep_THEP22 | 24.67 |
| mir122 | DBD_T0 | NoHep | DBD_T0.S.NoHep_THEP22 | 27.64 |
| mir103 | DBD_T0 | NoHep | DBD_T0.S.NoHep_THEP22 | 24.97 |
| mir103 | DBD_T0 | NoHep | DBD_T0.S.NoHep_THEP22 | 24.98 |
| mir39 | DBD_T0 | NoHep | DBD_T0.S.NoHep_THEP22 | 24.68 |
| mir191 | DBD_T0 | NoHep | DBD_T0.S.NoHep_THEP22 | 28.46 |
| UniSP4 | DBD_T0 | NoHep | DBD_T0.S.NoHep_THEP22 | 24.53 |
| mir148 | DBD_T0 | NoHep | DBD_T0.S.NoHep_THEP22 | 25.67 |
| mir148 | DBD_T0 | NoHep | DBD_T0.S.NoHep_THEP22 | 25.4 |
| mir122 | DBD_T0 | NoHep | DBD_T0.S.NoHep_THEP22 | 28.38 |
| UniSP4 | DBD_T0 | NoHep | DBD_T0.S.NoHep_THEP22 | 24.5 |
| mir191 | DBD_T0 | NoHep | DBD_T0.S.NoHep_THEP22 | 28.3 |
| mir39 | DBD_T0 | Hep6U | DBD_T0.S.Hep6U_THEP22 | 24.56 |
| mir103 | DBD_T0 | Hep6U | DBD_T0.S.Hep6U_THEP22 |  |
| mir191 | DBD_T0 | Hep6U | DBD_T0.S.Hep6U_THEP22 | 31.61 |
| mir122 | DBD_T0 | Hep6U | DBD_T0.S.Hep6U_THEP22 |  |
| mir122 | DBD_T0 | Hep6U | DBD_T0.S.Hep6U_THEP22 | 32.5 |
| mir39 | DBD_T0 | Hep6U | DBD_T0.S.Hep6U_THEP22 | 24.58 |
| mir191 | DBD_T0 | Hep6U | DBD_T0.S.Hep6U_THEP22 | 31.31 |
| UniSP4 | DBD_T0 | Hep6U | DBD_T0.S.Hep6U_THEP22 | 30.43 |
| mir148 | DBD_T0 | Hep6U | DBD_T0.S.Hep6U_THEP22 | 32.83 |
| UniSP4 | DBD_T0 | Hep6U | DBD_T0.S.Hep6U_THEP22 | 29.89 |
| mir148 | DBD_T0 | Hep6U | DBD_T0.S.Hep6U_THEP22 | 32.94 |
| mir39 | DBD_T0 | Hep6U | DBD_T0.S.Hep6U_THEP22 | 24.58 |
| UniSP4 | DBD_T0 | Hep6U | DBD_T0.S.Hep6U_THEP22 | 30.77 |
| mir103 | DBD_T0 | Hep6U | DBD_T0.S.Hep6U_THEP22 | 31.53 |
| mir148 | DBD_T0 | Hep6U | DBD_T0.S.Hep6U_THEP22 |  |
| mir191 | DBD_T0 | Hep6U | DBD_T0.S.Hep6U_THEP22 | 31.73 |
| mir122 | DBD_T0 | Hep6U | DBD_T0.S.Hep6U_THEP22 | 32.57 |
| mir103 | DBD_T0 | Hep6U | DBD_T0.S.Hep6U_THEP22 | 31.55 |
| mir191 | DBD_T0 | Hep12U | DBD_T0.S.Hep12U_THEP22 | 30.89 |
| UniSP4 | DBD_T0 | Hep12U | DBD_T0.S.Hep12U_THEP22 | 29.15 |
| mir103 | DBD_T0 | Hep12U | DBD_T0.S.Hep12U_THEP22 | 30.28 |
| mir39 | DBD_T0 | Hep12U | DBD_T0.S.Hep12U_THEP22 | 24.89 |
| mir39 | DBD_T0 | Hep12U | DBD_T0.S.Hep12U_THEP22 | 23.98 |
| mir148 | DBD_T0 | Hep12U | DBD_T0.S.Hep12U_THEP22 | 30.96 |
| mir103 | DBD_T0 | Hep12U | DBD_T0.S.Hep12U_THEP22 | 30.63 |
| mir39 | DBD_T0 | Hep12U | DBD_T0.S.Hep12U_THEP22 | 24.62 |
| mir191 | DBD_T0 | Hep12U | DBD_T0.S.Hep12U_THEP22 | 30.72 |
| mir122 | DBD_T0 | Hep12U | DBD_T0.S.Hep12U_THEP22 | 31.83 |
| mir103 | DBD_T0 | Hep12U | DBD_T0.S.Hep12U_THEP22 | 30.68 |
| mir148 | DBD_T0 | Hep12U | DBD_T0.S.Hep12U_THEP22 | 30.39 |
| mir122 | DBD_T0 | Hep12U | DBD_T0.S.Hep12U_THEP22 | 31.82 |
| mir122 | DBD_T0 | Hep12U | DBD_T0.S.Hep12U_THEP22 | 31.39 |
| UniSP4 | DBD_T0 | Hep12U | DBD_T0.S.Hep12U_THEP22 | 28.67 |
| mir191 | DBD_T0 | Hep12U | DBD_T0.S.Hep12U_THEP22 | 32.11 |
| UniSP4 | DBD_T0 | Hep12U | DBD_T0.S.Hep12U_THEP22 | 28.97 |
| mir148 | DBD_T0 | Hep12U | DBD_T0.S.Hep12U_THEP22 | 29.94 |
| mir122 | DBD_T0 | NoHep | DBD_T0.S.NoHep_THEP26 | 31.94 |
| mir122 | DBD_T0 | NoHep | DBD_T0.S.NoHep_THEP26 | 30.72 |
| mir103 | DBD_T0 | NoHep | DBD_T0.S.NoHep_THEP26 | 27.57 |
| mir148 | DBD_T0 | NoHep | DBD_T0.S.NoHep_THEP26 | 28.89 |
| mir39 | DBD_T0 | NoHep | DBD_T0.S.NoHep_THEP26 | 24.77 |
| UniSP4 | DBD_T0 | NoHep | DBD_T0.S.NoHep_THEP26 | 27.7 |
| UniSP4 | DBD_T0 | NoHep | DBD_T0.S.NoHep_THEP26 | 27.49 |
| mir148 | DBD_T0 | NoHep | DBD_T0.S.NoHep_THEP26 | 28.72 |
| mir191 | DBD_T0 | NoHep | DBD_T0.S.NoHep_THEP26 | 30.46 |
| mir39 | DBD_T0 | NoHep | DBD_T0.S.NoHep_THEP26 | 24.67 |
| mir103 | DBD_T0 | NoHep | DBD_T0.S.NoHep_THEP26 | 27.62 |
| mir103 | DBD_T0 | NoHep | DBD_T0.S.NoHep_THEP26 | 27.84 |
| mir191 | DBD_T0 | NoHep | DBD_T0.S.NoHep_THEP26 | 29.97 |
| UniSP4 | DBD_T0 | NoHep | DBD_T0.S.NoHep_THEP26 | 27.67 |
| mir148 | DBD_T0 | NoHep | DBD_T0.S.NoHep_THEP26 | 28.92 |
| mir191 | DBD_T0 | NoHep | DBD_T0.S.NoHep_THEP26 | 30.66 |
| mir39 | DBD_T0 | NoHep | DBD_T0.S.NoHep_THEP26 | 24.56 |
| mir122 | DBD_T0 | NoHep | DBD_T0.S.NoHep_THEP26 | 31.83 |
| mir39 | DBD_T0 | Hep6U | DBD_T0.S.Hep6U_THEP26 | 24.72 |
| mir191 | DBD_T0 | Hep6U | DBD_T0.S.Hep6U_THEP26 | 33.53 |
| mir148 | DBD_T0 | Hep6U | DBD_T0.S.Hep6U_THEP26 |  |
| mir122 | DBD_T0 | Hep6U | DBD_T0.S.Hep6U_THEP26 |  |
| mir103 | DBD_T0 | Hep6U | DBD_T0.S.Hep6U_THEP26 |  |
| mir191 | DBD_T0 | Hep6U | DBD_T0.S.Hep6U_THEP26 |  |
| mir122 | DBD_T0 | Hep6U | DBD_T0.S.Hep6U_THEP26 | 42.59 |
| mir191 | DBD_T0 | Hep6U | DBD_T0.S.Hep6U_THEP26 | 40.26 |
| UniSP4 | DBD_T0 | Hep6U | DBD_T0.S.Hep6U_THEP26 | 33.42 |
| mir39 | DBD_T0 | Hep6U | DBD_T0.S.Hep6U_THEP26 | 24.83 |
| UniSP4 | DBD_T0 | Hep6U | DBD_T0.S.Hep6U_THEP26 | 34.55 |
| mir103 | DBD_T0 | Hep6U | DBD_T0.S.Hep6U_THEP26 |  |
| mir39 | DBD_T0 | Hep6U | DBD_T0.S.Hep6U_THEP26 | 24.63 |
| UniSP4 | DBD_T0 | Hep6U | DBD_T0.S.Hep6U_THEP26 | 34.56 |
| mir103 | DBD_T0 | Hep6U | DBD_T0.S.Hep6U_THEP26 |  |
| mir148 | DBD_T0 | Hep6U | DBD_T0.S.Hep6U_THEP26 |  |
| mir122 | DBD_T0 | Hep6U | DBD_T0.S.Hep6U_THEP26 | 33.53 |
| mir148 | DBD_T0 | Hep6U | DBD_T0.S.Hep6U_THEP26 |  |
| mir122 | DBD_T0 | Hep12U | DBD_T0.S.Hep12U_THEP26 |  |
| mir103 | DBD_T0 | Hep12U | DBD_T0.S.Hep12U_THEP26 | 32.93 |
| mir191 | DBD_T0 | Hep12U | DBD_T0.S.Hep12U_THEP26 | 35.2 |
| mir39 | DBD_T0 | Hep12U | DBD_T0.S.Hep12U_THEP26 | 24.61 |
| mir103 | DBD_T0 | Hep12U | DBD_T0.S.Hep12U_THEP26 |  |
| mir191 | DBD_T0 | Hep12U | DBD_T0.S.Hep12U_THEP26 |  |
| mir103 | DBD_T0 | Hep12U | DBD_T0.S.Hep12U_THEP26 |  |
| mir148 | DBD_T0 | Hep12U | DBD_T0.S.Hep12U_THEP26 |  |
| UniSP4 | DBD_T0 | Hep12U | DBD_T0.S.Hep12U_THEP26 | 33.85 |
| mir148 | DBD_T0 | Hep12U | DBD_T0.S.Hep12U_THEP26 |  |
| UniSP4 | DBD_T0 | Hep12U | DBD_T0.S.Hep12U_THEP26 | 34.68 |
| mir39 | DBD_T0 | Hep12U | DBD_T0.S.Hep12U_THEP26 | 24.64 |
| mir122 | DBD_T0 | Hep12U | DBD_T0.S.Hep12U_THEP26 |  |
| mir148 | DBD_T0 | Hep12U | DBD_T0.S.Hep12U_THEP26 |  |
| UniSP4 | DBD_T0 | Hep12U | DBD_T0.S.Hep12U_THEP26 |  |
| mir122 | DBD_T0 | Hep12U | DBD_T0.S.Hep12U_THEP26 |  |
| mir191 | DBD_T0 | Hep12U | DBD_T0.S.Hep12U_THEP26 |  |
| mir39 | DBD_T0 | Hep12U | DBD_T0.S.Hep12U_THEP26 | 24.6 |
| UniSP4 | DBD_T0 | NoHep | DBD_T0.S.NoHep_THEP36 | 27.97 |
| mir39 | DBD_T0 | NoHep | DBD_T0.S.NoHep_THEP36 | 24.53 |
| mir39 | DBD_T0 | NoHep | DBD_T0.S.NoHep_THEP36 | 24.71 |
| mir103 | DBD_T0 | NoHep | DBD_T0.S.NoHep_THEP36 | 29.35 |
| mir103 | DBD_T0 | NoHep | DBD_T0.S.NoHep_THEP36 | 28.82 |
| mir148 | DBD_T0 | NoHep | DBD_T0.S.NoHep_THEP36 | 27.22 |
| UniSP4 | DBD_T0 | NoHep | DBD_T0.S.NoHep_THEP36 | 27.99 |
| mir191 | DBD_T0 | NoHep | DBD_T0.S.NoHep_THEP36 | 30.59 |
| UniSP4 | DBD_T0 | NoHep | DBD_T0.S.NoHep_THEP36 | 27.9 |
| mir39 | DBD_T0 | NoHep | DBD_T0.S.NoHep_THEP36 | 24.71 |
| mir191 | DBD_T0 | NoHep | DBD_T0.S.NoHep_THEP36 | 30.38 |
| mir148 | DBD_T0 | NoHep | DBD_T0.S.NoHep_THEP36 | 27.37 |
| mir191 | DBD_T0 | NoHep | DBD_T0.S.NoHep_THEP36 | 30.37 |
| mir122 | DBD_T0 | NoHep | DBD_T0.S.NoHep_THEP36 | 26.76 |
| mir122 | DBD_T0 | NoHep | DBD_T0.S.NoHep_THEP36 | 26.81 |
| mir103 | DBD_T0 | NoHep | DBD_T0.S.NoHep_THEP36 | 29,00 |
| mir122 | DBD_T0 | NoHep | DBD_T0.S.NoHep_THEP36 | 26.49 |
| mir148 | DBD_T0 | NoHep | DBD_T0.S.NoHep_THEP36 | 27.33 |
| UniSP4 | DBD_T0 | Hep6U | DBD_T0.S.Hep6U_THEP36 | 34.9 |
| mir39 | DBD_T0 | Hep6U | DBD_T0.S.Hep6U_THEP36 | 24.74 |
| mir191 | DBD_T0 | Hep6U | DBD_T0.S.Hep6U_THEP36 |  |
| mir39 | DBD_T0 | Hep6U | DBD_T0.S.Hep6U_THEP36 | 24.72 |
| mir122 | DBD_T0 | Hep6U | DBD_T0.S.Hep6U_THEP36 | 31.62 |
| mir191 | DBD_T0 | Hep6U | DBD_T0.S.Hep6U_THEP36 | 38.19 |
| mir122 | DBD_T0 | Hep6U | DBD_T0.S.Hep6U_THEP36 | 32.66 |
| mir103 | DBD_T0 | Hep6U | DBD_T0.S.Hep6U_THEP36 |  |
| mir122 | DBD_T0 | Hep6U | DBD_T0.S.Hep6U_THEP36 | 31.87 |
| mir39 | DBD_T0 | Hep6U | DBD_T0.S.Hep6U_THEP36 | 24.52 |
| mir148 | DBD_T0 | Hep6U | DBD_T0.S.Hep6U_THEP36 |  |
| mir103 | DBD_T0 | Hep6U | DBD_T0.S.Hep6U_THEP36 |  |
| UniSP4 | DBD_T0 | Hep6U | DBD_T0.S.Hep6U_THEP36 | 33.86 |
| mir191 | DBD_T0 | Hep6U | DBD_T0.S.Hep6U_THEP36 |  |
| mir103 | DBD_T0 | Hep6U | DBD_T0.S.Hep6U_THEP36 |  |
| UniSP4 | DBD_T0 | Hep6U | DBD_T0.S.Hep6U_THEP36 | 42.57 |
| mir148 | DBD_T0 | Hep6U | DBD_T0.S.Hep6U_THEP36 |  |
| mir148 | DBD_T0 | Hep6U | DBD_T0.S.Hep6U_THEP36 | 34.27 |
| UniSP4 | DBD_T0 | Hep12U | DBD_T0.S.Hep12_THEP36 | 33.42 |
| mir39 | DBD_T0 | Hep12U | DBD_T0.S.Hep12_THEP36 | 24.76 |
| mir122 | DBD_T0 | Hep12U | DBD_T0.S.Hep12_THEP36 | 29.89 |
| mir148 | DBD_T0 | Hep12U | DBD_T0.S.Hep12_THEP36 |  |
| mir122 | DBD_T0 | Hep12U | DBD_T0.S.Hep12_THEP36 | 30.44 |
| mir148 | DBD_T0 | Hep12U | DBD_T0.S.Hep12_THEP36 | 32.4 |
| UniSP4 | DBD_T0 | Hep12U | DBD_T0.S.Hep12_THEP36 | 34.21 |
| mir39 | DBD_T0 | Hep12U | DBD_T0.S.Hep12_THEP36 | 24.68 |
| mir191 | DBD_T0 | Hep12U | DBD_T0.S.Hep12_THEP36 | 34.96 |
| mir103 | DBD_T0 | Hep12U | DBD_T0.S.Hep12_THEP36 |  |
| mir148 | DBD_T0 | Hep12U | DBD_T0.S.Hep12_THEP36 | 32.95 |
| mir122 | DBD_T0 | Hep12U | DBD_T0.S.Hep12_THEP36 | 30.46 |
| mir39 | DBD_T0 | Hep12U | DBD_T0.S.Hep12_THEP36 | 24.84 |
| UniSP4 | DBD_T0 | Hep12U | DBD_T0.S.Hep12_THEP36 | 34.3 |
| mir103 | DBD_T0 | Hep12U | DBD_T0.S.Hep12_THEP36 | 32.38 |
| mir191 | DBD_T0 | Hep12U | DBD_T0.S.Hep12_THEP36 |  |
| mir103 | DBD_T0 | Hep12U | DBD_T0.S.Hep12_THEP36 |  |
| mir191 | DBD_T0 | Hep12U | DBD_T0.S.Hep12_THEP36 | 34.8 |

**D.**

| **miRNAs** | **Donor Group** | **Treatment** | **Sample Name** | **Ct** |
| --- | --- | --- | --- | --- |
| miR122 | DCD_T0 | NoHep | DCD_T0.S.NoHep_THEP8 | 27.51 |
| miR122 | DCD_T0 | NoHep | DCD_T0.S.NoHep_THEP8 | 27.51 |
| miR122 | DCD_T0 | NoHep | DCD_T0.S.NoHep_THEP8 | 27.61 |
| miR148 | DCD_T0 | NoHep | DCD_T0.S.NoHep_THEP8 | 30.28 |
| miR148 | DCD_T0 | NoHep | DCD_T0.S.NoHep_THEP8 | 30.49 |
| miR148 | DCD_T0 | NoHep | DCD_T0.S.NoHep_THEP8 | 30.21 |
| miR103 | DCD_T0 | NoHep | DCD_T0.S.NoHep_THEP8 | 32.55 |
| miR103 | DCD_T0 | NoHep | DCD_T0.S.NoHep_THEP8 | 32.25 |
| miR103 | DCD_T0 | NoHep | DCD_T0.S.NoHep_THEP8 | 32.63 |
| miR191 | DCD_T0 | NoHep | DCD_T0.S.NoHep_THEP8 | 32.1 |
| miR191 | DCD_T0 | NoHep | DCD_T0.S.NoHep_THEP8 | 31.77 |
| miR191 | DCD_T0 | NoHep | DCD_T0.S.NoHep_THEP8 | 31.76 |
| UNISP4 | DCD_T0 | NoHep | DCD_T0.S.NoHep_THEP8 | 28.7 |
| UNISP4 | DCD_T0 | NoHep | DCD_T0.S.NoHep_THEP8 | 29.02 |
| UNISP4 | DCD_T0 | NoHep | DCD_T0.S.NoHep_THEP8 | 28.81 |
| miR39 | DCD_T0 | NoHep | DCD_T0.S.NoHep_THEP8 | 27.93 |
| miR39 | DCD_T0 | NoHep | DCD_T0.S.NoHep_THEP8 | 27.79 |
| miR39 | DCD_T0 | NoHep | DCD_T0.S.NoHep_THEP8 | 27.95 |
| miR122 | DCD_T0 | NoHep | DCD_T0.S.NoHep_THEP11 | 33.64 |
| miR122 | DCD_T0 | NoHep | DCD_T0.S.NoHep_THEP11 | 33.83 |
| miR122 | DCD_T0 | NoHep | DCD_T0.S.NoHep_THEP11 | 34.54 |
| miR148 | DCD_T0 | NoHep | DCD_T0.S.NoHep_THEP11 | 32.02 |
| miR148 | DCD_T0 | NoHep | DCD_T0.S.NoHep_THEP11 | 32.09 |
| miR148 | DCD_T0 | NoHep | DCD_T0.S.NoHep_THEP11 | 31.68 |
| miR103 | DCD_T0 | NoHep | DCD_T0.S.NoHep_THEP11 | 33.07 |
| miR103 | DCD_T0 | NoHep | DCD_T0.S.NoHep_THEP11 | 32.25 |
| miR103 | DCD_T0 | NoHep | DCD_T0.S.NoHep_THEP11 | 32.53 |
| miR191 | DCD_T0 | NoHep | DCD_T0.S.NoHep_THEP11 | 28.03 |
| miR191 | DCD_T0 | NoHep | DCD_T0.S.NoHep_THEP11 | 28.03 |
| miR191 | DCD_T0 | NoHep | DCD_T0.S.NoHep_THEP11 | 28.12 |
| UNISP4 | DCD_T0 | NoHep | DCD_T0.S.NoHep_THEP11 | 31.62 |
| UNISP4 | DCD_T0 | NoHep | DCD_T0.S.NoHep_THEP11 | 31.96 |
| UNISP4 | DCD_T0 | NoHep | DCD_T0.S.NoHep_THEP11 | 32.17 |
| miR39 | DCD_T0 | NoHep | DCD_T0.S.NoHep_THEP11 | 28.86 |
| miR39 | DCD_T0 | NoHep | DCD_T0.S.NoHep_THEP11 | 28.93 |
| miR39 | DCD_T0 | NoHep | DCD_T0.S.NoHep_THEP11 | 28.86 |
| miR122 | DCD_T0 | NoHep | DCD_T0.S.NoHep_THEP33 | 31.94 |
| miR122 | DCD_T0 | NoHep | DCD_T0.S.NoHep_THEP33 | 31.5 |
| miR122 | DCD_T0 | NoHep | DCD_T0.S.NoHep_THEP33 | 31.76 |
| miR148 | DCD_T0 | NoHep | DCD_T0.S.NoHep_THEP33 | 32.08 |
| miR148 | DCD_T0 | NoHep | DCD_T0.S.NoHep_THEP33 | 32.11 |
| miR148 | DCD_T0 | NoHep | DCD_T0.S.NoHep_THEP33 | 31.69 |
| miR103 | DCD_T0 | NoHep | DCD_T0.S.NoHep_THEP33 | 32.23 |
| miR103 | DCD_T0 | NoHep | DCD_T0.S.NoHep_THEP33 | 32.17 |
| miR103 | DCD_T0 | NoHep | DCD_T0.S.NoHep_THEP33 | 32.23 |
| miR191 | DCD_T0 | NoHep | DCD_T0.S.NoHep_THEP33 | 32.14 |
| miR191 | DCD_T0 | NoHep | DCD_T0.S.NoHep_THEP33 | 31.67 |
| miR191 | DCD_T0 | NoHep | DCD_T0.S.NoHep_THEP33 | 32.22 |
| UNISP4 | DCD_T0 | NoHep | DCD_T0.S.NoHep_THEP33 | 28.97 |
| UNISP4 | DCD_T0 | NoHep | DCD_T0.S.NoHep_THEP33 | 28.94 |
| UNISP4 | DCD_T0 | NoHep | DCD_T0.S.NoHep_THEP33 | 29.16 |
| miR39 | DCD_T0 | NoHep | DCD_T0.S.NoHep_THEP33 | 27.82 |
| miR39 | DCD_T0 | NoHep | DCD_T0.S.NoHep_THEP33 | 27.87 |
| miR39 | DCD_T0 | NoHep | DCD_T0.S.NoHep_THEP33 | 27.77 |
| miR122 | DCD_T0 | NoHep | DCD_T0.S.NoHep_THEP34 | 31.83 |
| miR122 | DCD_T0 | NoHep | DCD_T0.S.NoHep_THEP34 | 31.92 |
| miR122 | DCD_T0 | NoHep | DCD_T0.S.NoHep_THEP34 | 31.72 |
| miR148 | DCD_T0 | NoHep | DCD_T0.S.NoHep_THEP34 | 35.48 |
| miR148 | DCD_T0 | NoHep | DCD_T0.S.NoHep_THEP34 | 34.75 |
| miR148 | DCD_T0 | NoHep | DCD_T0.S.NoHep_THEP34 | 35.56 |
| miR103 | DCD_T0 | NoHep | DCD_T0.S.NoHep_THEP34 | 35.04 |
| miR103 | DCD_T0 | NoHep | DCD_T0.S.NoHep_THEP34 | 35.15 |
| miR103 | DCD_T0 | NoHep | DCD_T0.S.NoHep_THEP34 | 34.72 |
| miR191 | DCD_T0 | NoHep | DCD_T0.S.NoHep_THEP34 | 32.22 |
| miR191 | DCD_T0 | NoHep | DCD_T0.S.NoHep_THEP34 | 32.86 |
| miR191 | DCD_T0 | NoHep | DCD_T0.S.NoHep_THEP34 | 32.11 |
| UNISP4 | DCD_T0 | NoHep | DCD_T0.S.NoHep_THEP34 | 30.11 |
| UNISP4 | DCD_T0 | NoHep | DCD_T0.S.NoHep_THEP34 | 29.8 |
| UNISP4 | DCD_T0 | NoHep | DCD_T0.S.NoHep_THEP34 | 30.23 |
| miR39 | DCD_T0 | NoHep | DCD_T0.S.NoHep_THEP34 | 27.76 |
| miR39 | DCD_T0 | NoHep | DCD_T0.S.NoHep_THEP34 | 27.92 |
| miR39 | DCD_T0 | NoHep | DCD_T0.S.NoHep_THEP34 | 28.11 |
| miR122 | DCD_T1 | NoHep | DCD_T1.S.NoHep_THEP8 | 29.94 |
| miR122 | DCD_T1 | NoHep | DCD_T1.S.NoHep_THEP8 | 30.17 |
| miR122 | DCD_T1 | NoHep | DCD_T1.S.NoHep_THEP8 | 30.13 |
| miR148 | DCD_T1 | NoHep | DCD_T1.S.NoHep_THEP8 | 30.29 |
| miR148 | DCD_T1 | NoHep | DCD_T1.S.NoHep_THEP8 | 30.26 |
| miR148 | DCD_T1 | NoHep | DCD_T1.S.NoHep_THEP8 | 30.06 |
| miR103 | DCD_T1 | NoHep | DCD_T1.S.NoHep_THEP8 | 30.7 |
| miR103 | DCD_T1 | NoHep | DCD_T1.S.NoHep_THEP8 | 30.64 |
| miR103 | DCD_T1 | NoHep | DCD_T1.S.NoHep_THEP8 | 30.67 |
| miR191 | DCD_T1 | NoHep | DCD_T1.S.NoHep_THEP8 | 29.01 |
| miR191 | DCD_T1 | NoHep | DCD_T1.S.NoHep_THEP8 | 29.11 |
| miR191 | DCD_T1 | NoHep | DCD_T1.S.NoHep_THEP8 | 29.02 |
| UNISP4 | DCD_T1 | NoHep | DCD_T1.S.NoHep_THEP8 | 27.9 |
| UNISP4 | DCD_T1 | NoHep | DCD_T1.S.NoHep_THEP8 | 27.88 |
| UNISP4 | DCD_T1 | NoHep | DCD_T1.S.NoHep_THEP8 | 28.05 |
| miR39 | DCD_T1 | NoHep | DCD_T1.S.NoHep_THEP8 | 28.01 |
| miR39 | DCD_T1 | NoHep | DCD_T1.S.NoHep_THEP8 | 28.2 |
| miR39 | DCD_T1 | NoHep | DCD_T1.S.NoHep_THEP8 | 28.05 |
| miR122 | DCD_T1 | NoHep | DCD_T1.S.NoHep_THEP11 | 33.63 |
| miR122 | DCD_T1 | NoHep | DCD_T1.S.NoHep_THEP11 | 34.62 |
| miR122 | DCD_T1 | NoHep | DCD_T1.S.NoHep_THEP11 | 33.29 |
| miR148 | DCD_T1 | NoHep | DCD_T1.S.NoHep_THEP11 | 30.44 |
| miR148 | DCD_T1 | NoHep | DCD_T1.S.NoHep_THEP11 | 30.43 |
| miR148 | DCD_T1 | NoHep | DCD_T1.S.NoHep_THEP11 | 30.5 |
| miR103 | DCD_T1 | NoHep | DCD_T1.S.NoHep_THEP11 | 29.17 |
| miR103 | DCD_T1 | NoHep | DCD_T1.S.NoHep_THEP11 | 29.11 |
| miR103 | DCD_T1 | NoHep | DCD_T1.S.NoHep_THEP11 | 29.21 |
| miR191 | DCD_T1 | NoHep | DCD_T1.S.NoHep_THEP11 | 27.28 |
| miR191 | DCD_T1 | NoHep | DCD_T1.S.NoHep_THEP11 | 27.43 |
| miR191 | DCD_T1 | NoHep | DCD_T1.S.NoHep_THEP11 | 27.49 |
| UNISP4 | DCD_T1 | NoHep | DCD_T1.S.NoHep_THEP11 | 28.63 |
| UNISP4 | DCD_T1 | NoHep | DCD_T1.S.NoHep_THEP11 | 28.69 |
| UNISP4 | DCD_T1 | NoHep | DCD_T1.S.NoHep_THEP11 | 28.62 |
| miR39 | DCD_T1 | NoHep | DCD_T1.S.NoHep_THEP11 | 28.05 |
| miR39 | DCD_T1 | NoHep | DCD_T1.S.NoHep_THEP11 | 28.13 |
| miR39 | DCD_T1 | NoHep | DCD_T1.S.NoHep_THEP11 | 28.09 |
| miR122 | DCD_T1 | NoHep | DCD_T1.S.NoHep_THEP33 | 31.57 |
| miR122 | DCD_T1 | NoHep | DCD_T1.S.NoHep_THEP33 | 31.3 |
| miR122 | DCD_T1 | NoHep | DCD_T1.S.NoHep_THEP33 | 31.18 |
| miR148 | DCD_T1 | NoHep | DCD_T1.S.NoHep_THEP33 | 30.84 |
| miR148 | DCD_T1 | NoHep | DCD_T1.S.NoHep_THEP33 | 30.96 |
| miR148 | DCD_T1 | NoHep | DCD_T1.S.NoHep_THEP33 | 30.7 |
| miR103 | DCD_T1 | NoHep | DCD_T1.S.NoHep_THEP33 | 32.92 |
| miR103 | DCD_T1 | NoHep | DCD_T1.S.NoHep_THEP33 | 33.04 |
| miR103 | DCD_T1 | NoHep | DCD_T1.S.NoHep_THEP33 | 33.13 |
| miR191 | DCD_T1 | NoHep | DCD_T1.S.NoHep_THEP33 | 29.97 |
| miR191 | DCD_T1 | NoHep | DCD_T1.S.NoHep_THEP33 | 30.27 |
| miR191 | DCD_T1 | NoHep | DCD_T1.S.NoHep_THEP33 | 30.22 |
| UNISP4 | DCD_T1 | NoHep | DCD_T1.S.NoHep_THEP33 | 27.81 |
| UNISP4 | DCD_T1 | NoHep | DCD_T1.S.NoHep_THEP33 | 27.79 |
| UNISP4 | DCD_T1 | NoHep | DCD_T1.S.NoHep_THEP33 | 27.9 |
| miR39 | DCD_T1 | NoHep | DCD_T1.S.NoHep_THEP33 | 27.91 |
| miR39 | DCD_T1 | NoHep | DCD_T1.S.NoHep_THEP33 | 28.15 |
| miR39 | DCD_T1 | NoHep | DCD_T1.S.NoHep_THEP33 | 28.07 |
| miR122 | DCD_T1 | NoHep | DCD_T1.S.NoHep_THEP34 | 30.25 |
| miR122 | DCD_T1 | NoHep | DCD_T1.S.NoHep_THEP34 | 30.07 |
| miR122 | DCD_T1 | NoHep | DCD_T1.S.NoHep_THEP34 | 30.19 |
| miR148 | DCD_T1 | NoHep | DCD_T1.S.NoHep_THEP34 | 31.14 |
| miR148 | DCD_T1 | NoHep | DCD_T1.S.NoHep_THEP34 | 31.47 |
| miR148 | DCD_T1 | NoHep | DCD_T1.S.NoHep_THEP34 | 31.3 |
| miR103 | DCD_T1 | NoHep | DCD_T1.S.NoHep_THEP34 | 32.95 |
| miR103 | DCD_T1 | NoHep | DCD_T1.S.NoHep_THEP34 | 33.34 |
| miR103 | DCD_T1 | NoHep | DCD_T1.S.NoHep_THEP34 | 33.92 |
| miR191 | DCD_T1 | NoHep | DCD_T1.S.NoHep_THEP34 | 29.13 |
| miR191 | DCD_T1 | NoHep | DCD_T1.S.NoHep_THEP34 | 29.22 |
| miR191 | DCD_T1 | NoHep | DCD_T1.S.NoHep_THEP34 | 29.32 |
| UNISP4 | DCD_T1 | NoHep | DCD_T1.S.NoHep_THEP34 | 29.06 |
| UNISP4 | DCD_T1 | NoHep | DCD_T1.S.NoHep_THEP34 | 29.04 |
| UNISP4 | DCD_T1 | NoHep | DCD_T1.S.NoHep_THEP34 | 29.07 |
| miR39 | DCD_T1 | NoHep | DCD_T1.S.NoHep_THEP34 | 28.27 |
| miR39 | DCD_T1 | NoHep | DCD_T1.S.NoHep_THEP34 | 28.22 |
| miR39 | DCD_T1 | NoHep | DCD_T1.S.NoHep_THEP34 | 28.16 |
| miR122 | DBD_T0 | NoHep | DBD_T0.S.NoHep_THEP15 | 32.68 |
| miR122 | DBD_T0 | NoHep | DBD_T0.S.NoHep_THEP15 | 32.61 |
| miR122 | DBD_T0 | NoHep | DBD_T0.S.NoHep_THEP15 | 32.18 |
| miR148 | DBD_T0 | NoHep | DBD_T0.S.NoHep_THEP15 | 32.43 |
| miR148 | DBD_T0 | NoHep | DBD_T0.S.NoHep_THEP15 | 32.33 |
| miR148 | DBD_T0 | NoHep | DBD_T0.S.NoHep_THEP15 | 32.59 |
| miR103 | DBD_T0 | NoHep | DBD_T0.S.NoHep_THEP15 | 32.05 |
| miR103 | DBD_T0 | NoHep | DBD_T0.S.NoHep_THEP15 | 32.07 |
| miR103 | DBD_T0 | NoHep | DBD_T0.S.NoHep_THEP15 | 32.06 |
| miR191 | DBD_T0 | NoHep | DBD_T0.S.NoHep_THEP15 | 31.63 |
| miR191 | DBD_T0 | NoHep | DBD_T0.S.NoHep_THEP15 | 31.64 |
| miR191 | DBD_T0 | NoHep | DBD_T0.S.NoHep_THEP15 | 31.65 |
| UNISP4 | DBD_T0 | NoHep | DBD_T0.S.NoHep_THEP15 | 27.82 |
| UNISP4 | DBD_T0 | NoHep | DBD_T0.S.NoHep_THEP15 | 27.97 |
| UNISP4 | DBD_T0 | NoHep | DBD_T0.S.NoHep_THEP15 | 27.76 |
| miR39 | DBD_T0 | NoHep | DBD_T0.S.NoHep_THEP15 | 27.6 |
| miR39 | DBD_T0 | NoHep | DBD_T0.S.NoHep_THEP15 | 27.75 |
| miR39 | DBD_T0 | NoHep | DBD_T0.S.NoHep_THEP15 | 27.78 |
| miR122 | DBD_T0 | NoHep | DBD_T0.S.NoHep_THEP22 | 30.51 |
| miR122 | DBD_T0 | NoHep | DBD_T0.S.NoHep_THEP22 | 30.61 |
| miR122 | DBD_T0 | NoHep | DBD_T0.S.NoHep_THEP22 | 30.14 |
| miR148 | DBD_T0 | NoHep | DBD_T0.S.NoHep_THEP22 | 30.69 |
| miR148 | DBD_T0 | NoHep | DBD_T0.S.NoHep_THEP22 | 30.65 |
| miR148 | DBD_T0 | NoHep | DBD_T0.S.NoHep_THEP22 | 30.53 |
| miR103 | DBD_T0 | NoHep | DBD_T0.S.NoHep_THEP22 | 30.78 |
| miR103 | DBD_T0 | NoHep | DBD_T0.S.NoHep_THEP22 | 30.6 |
| miR103 | DBD_T0 | NoHep | DBD_T0.S.NoHep_THEP22 | 30.84 |
| miR191 | DBD_T0 | NoHep | DBD_T0.S.NoHep_THEP22 | 30.58 |
| miR191 | DBD_T0 | NoHep | DBD_T0.S.NoHep_THEP22 | 30.52 |
| miR191 | DBD_T0 | NoHep | DBD_T0.S.NoHep_THEP22 | 30.47 |
| UNISP4 | DBD_T0 | NoHep | DBD_T0.S.NoHep_THEP22 | 26.77 |
| UNISP4 | DBD_T0 | NoHep | DBD_T0.S.NoHep_THEP22 | 26.77 |
| UNISP4 | DBD_T0 | NoHep | DBD_T0.S.NoHep_THEP22 | 26.66 |
| miR39 | DBD_T0 | NoHep | DBD_T0.S.NoHep_THEP22 | 27.68 |
| miR39 | DBD_T0 | NoHep | DBD_T0.S.NoHep_THEP22 | 27.85 |
| miR39 | DBD_T0 | NoHep | DBD_T0.S.NoHep_THEP22 | 27.82 |
| miR122 | DBD_T0 | NoHep | DBD_T0.S.NoHep_THEP26 | 35.77 |
| miR122 | DBD_T0 | NoHep | DBD_T0.S.NoHep_THEP26 | 35.17 |
| miR122 | DBD_T0 | NoHep | DBD_T0.S.NoHep_THEP26 | 38.43 |
| miR148 | DBD_T0 | NoHep | DBD_T0.S.NoHep_THEP26 | 34.48 |
| miR148 | DBD_T0 | NoHep | DBD_T0.S.NoHep_THEP26 | 35.66 |
| miR148 | DBD_T0 | NoHep | DBD_T0.S.NoHep_THEP26 | 34.7 |
| miR103 | DBD_T0 | NoHep | DBD_T0.S.NoHep_THEP26 | 33.77 |
| miR103 | DBD_T0 | NoHep | DBD_T0.S.NoHep_THEP26 | 33.31 |
| miR103 | DBD_T0 | NoHep | DBD_T0.S.NoHep_THEP26 | 33.96 |
| miR191 | DBD_T0 | NoHep | DBD_T0.S.NoHep_THEP26 | 32.55 |
| miR191 | DBD_T0 | NoHep | DBD_T0.S.NoHep_THEP26 | 32.72 |
| miR191 | DBD_T0 | NoHep | DBD_T0.S.NoHep_THEP26 | 32.69 |
| UNISP4 | DBD_T0 | NoHep | DBD_T0.S.NoHep_THEP26 | 30.34 |
| UNISP4 | DBD_T0 | NoHep | DBD_T0.S.NoHep_THEP26 | 30.51 |
| UNISP4 | DBD_T0 | NoHep | DBD_T0.S.NoHep_THEP26 | 30.3 |
| miR39 | DBD_T0 | NoHep | DBD_T0.S.NoHep_THEP26 | 27.99 |
| miR39 | DBD_T0 | NoHep | DBD_T0.S.NoHep_THEP26 | 27.97 |
| miR39 | DBD_T0 | NoHep | DBD_T0.S.NoHep_THEP26 | 28.04 |
| miR122 | DBD_T0 | NoHep | DBD_T0.S.NoHep_THEP36 | 29.31 |
| miR122 | DBD_T0 | NoHep | DBD_T0.S.NoHep_THEP36 | 29.54 |
| miR122 | DBD_T0 | NoHep | DBD_T0.S.NoHep_THEP36 | 29.3 |
| miR148 | DBD_T0 | NoHep | DBD_T0.S.NoHep_THEP36 | 32.57 |
| miR148 | DBD_T0 | NoHep | DBD_T0.S.NoHep_THEP36 | 32.94 |
| miR148 | DBD_T0 | NoHep | DBD_T0.S.NoHep_THEP36 | 32.82 |
| miR103 | DBD_T0 | NoHep | DBD_T0.S.NoHep_THEP36 | 34.42 |
| miR103 | DBD_T0 | NoHep | DBD_T0.S.NoHep_THEP36 | 34.13 |
| miR103 | DBD_T0 | NoHep | DBD_T0.S.NoHep_THEP36 | 33.66 |
| miR191 | DBD_T0 | NoHep | DBD_T0.S.NoHep_THEP36 | 32.87 |
| miR191 | DBD_T0 | NoHep | DBD_T0.S.NoHep_THEP36 | 32.48 |
| miR191 | DBD_T0 | NoHep | DBD_T0.S.NoHep_THEP36 | 32.78 |
| UNISP4 | DBD_T0 | NoHep | DBD_T0.S.NoHep_THEP36 | 30.51 |
| UNISP4 | DBD_T0 | NoHep | DBD_T0.S.NoHep_THEP36 | 30.33 |
| UNISP4 | DBD_T0 | NoHep | DBD_T0.S.NoHep_THEP36 | 30.7 |
| miR39 | DBD_T0 | NoHep | DBD_T0.S.NoHep_THEP36 | 27.88 |
| miR39 | DBD_T0 | NoHep | DBD_T0.S.NoHep_THEP36 | 27.93 |
| miR39 | DBD_T0 | NoHep | DBD_T0.S.NoHep_THEP36 | 27.85 |
