# Supplementary material for: Heparinase I treatment to overcome RNA quantification interference in heparinized liver donor samples: One size fits all?
Source: PLoS One. 2025 May 12;20(5):e0322899. doi: 10.1371/journal.pone.0322899 (PMC12068581; doi:10.1371/journal.pone.0322899)
Supplement: S4 Table — (DOCX) [file pone.0322899.s004.docx]

**S4 Table. Clinical characteristics and raw data Ct values of patients undergoing cardiac surgery with cardiopulmonary bypass.**

1. Demographic and analytic details. (B) Raw data Ct triplicates in non-heparinized (Control_Non_Heparinized) and heparinized (Control_Heparinized) paired serum samples without heparinase (NoHep), with 6 IU (Hep6U) and 12 IU (Hep12U).

**A.**

| **Age (y)** | **Sex** | **BMI** | **AST (IU/L)** | **ALT(IU/L)** | **Bilirubin mg/dl** |
| --- | --- | --- | --- | --- | --- |
| 75 | Male | 25.31 | 27 | 27 | 0.64 |
| 54 | Male | 30.04 | 18 | 17 | **-** |
| 72 | Male | 31.56 | 19 | 10 | 0.62 |
| 52 | Male | 24.06 | 25 | 29 | 0.62 |

**B.**

| **miRNAs** | **Control Group** | **Treatment** | **Sample Name** | **Ct** |
| --- | --- | --- | --- | --- |
| mir103 | Control _Non_Heparinized | NoHep | CNonHep.S.NoHep_THEP41 | 27.91 |
| mir191 | Control _Non_Heparinized | NoHep | CNonHep.S.NoHep_THEP41 | 30.43 |
| mir103 | Control _Non_Heparinized | NoHep | CNonHep.S.NoHep_THEP41 | 28.35 |
| mir191 | Control _Non_Heparinized | NoHep | CNonHep.S.NoHep_THEP41 | 30.2 |
| mir122 | Control _Non_Heparinized | NoHep | CNonHep.S.NoHep_THEP41 | 28.97 |
| mir148 | Control _Non_Heparinized | NoHep | CNonHep.S.NoHep_THEP41 | 28.79 |
| UniSP4 | Control _Non_Heparinized | NoHep | CNonHep.S.NoHep_THEP41 | 27.55 |
| mir39 | Control _Non_Heparinized | NoHep | CNonHep.S.NoHep_THEP41 | 24.73 |
| mir191 | Control _Non_Heparinized | NoHep | CNonHep.S.NoHep_THEP41 | 30.44 |
| mir103 | Control _Non_Heparinized | NoHep | CNonHep.S.NoHep_THEP41 | 27.9 |
| mir148 | Control _Non_Heparinized | NoHep | CNonHep.S.NoHep_THEP41 | 28.41 |
| mir122 | Control _Non_Heparinized | NoHep | CNonHep.S.NoHep_THEP41 | 29.64 |
| mir39 | Control _Non_Heparinized | NoHep | CNonHep.S.NoHep_THEP41 | 24.99 |
| UniSP4 | Control _Non_Heparinized | NoHep | CNonHep.S.NoHep_THEP41 | 27.19 |
| mir39 | Control _Non_Heparinized | NoHep | CNonHep.S.NoHep_THEP41 | 24.87 |
| UniSP4 | Control _Non_Heparinized | NoHep | CNonHep.S.NoHep_THEP41 | 27.45 |
| mir148 | Control _Non_Heparinized | NoHep | CNonHep.S.NoHep_THEP41 | 28.38 |
| mir122 | Control _Non_Heparinized | NoHep | CNonHep.S.NoHep_THEP41 | 29.72 |
| mir39 | Control _Non_Heparinized | Hep6U | CNonHep.S.Hep6U_THEP41 | 24.79 |
| mir39 | Control _Non_Heparinized | Hep6U | CNonHep.S.Hep6U_THEP41 | 24.81 |
| mir122 | Control _Non_Heparinized | Hep6U | CNonHep.S.Hep6U_THEP41 | 33.44 |
| mir191 | Control _Non_Heparinized | Hep6U | CNonHep.S.Hep6U_THEP41 | 33.26 |
| mir103 | Control _Non_Heparinized | Hep6U | CNonHep.S.Hep6U_THEP41 | 32.83 |
| mir122 | Control _Non_Heparinized | Hep6U | CNonHep.S.Hep6U_THEP41 | 31.4 |
| mir191 | Control _Non_Heparinized | Hep6U | CNonHep.S.Hep6U_THEP41 | 33.17 |
| mir39 | Control _Non_Heparinized | Hep6U | CNonHep.S.Hep6U_THEP41 | 24.72 |
| mir148 | Control _Non_Heparinized | Hep6U | CNonHep.S.Hep6U_THEP41 | 32.77 |
| UniSP4 | Control _Non_Heparinized | Hep6U | CNonHep.S.Hep6U_THEP41 | 31.57 |
| mir191 | Control _Non_Heparinized | Hep6U | CNonHep.S.Hep6U_THEP41 | 33.72 |
| UniSP4 | Control _Non_Heparinized | Hep6U | CNonHep.S.Hep6U_THEP41 | 32.25 |
| mir103 | Control _Non_Heparinized | Hep6U | CNonHep.S.Hep6U_THEP41 | 31.68 |
| UniSP4 | Control _Non_Heparinized | Hep6U | CNonHep.S.Hep6U_THEP41 | 31.26 |
| mir148 | Control _Non_Heparinized | Hep6U | CNonHep.S.Hep6U_THEP41 |  |
| mir103 | Control _Non_Heparinized | Hep6U | CNonHep.S.Hep6U_THEP41 | 31.71 |
| mir148 | Control _Non_Heparinized | Hep6U | CNonHep.S.Hep6U_THEP41 | 35.45 |
| mir122 | Control _Non_Heparinized | Hep6U | CNonHep.S.Hep6U_THEP41 | 31.97 |
| mir39 | Control _Non_Heparinized | Hep12U | CNonHep.S.Hep12U_THEP41 | 24.77 |
| UniSP4 | Control _Non_Heparinized | Hep12U | CNonHep.S.Hep12U_THEP41 | 32.71 |
| mir191 | Control _Non_Heparinized | Hep12U | CNonHep.S.Hep12U_THEP41 | 34.88 |
| UniSP4 | Control _Non_Heparinized | Hep12U | CNonHep.S.Hep12U_THEP41 | 33.19 |
| UniSP4 | Control _Non_Heparinized | Hep12U | CNonHep.S.Hep12U_THEP41 | 32.23 |
| mir103 | Control _Non_Heparinized | Hep12U | CNonHep.S.Hep12U_THEP41 |  |
| mir39 | Control _Non_Heparinized | Hep12U | CNonHep.S.Hep12U_THEP41 | 24.74 |
| mir103 | Control _Non_Heparinized | Hep12U | CNonHep.S.Hep12U_THEP41 | 33.38 |
| mir148 | Control _Non_Heparinized | Hep12U | CNonHep.S.Hep12U_THEP41 |  |
| mir122 | Control _Non_Heparinized | Hep12U | CNonHep.S.Hep12U_THEP41 | 32.45 |
| mir103 | Control _Non_Heparinized | Hep12U | CNonHep.S.Hep12U_THEP41 | 33.24 |
| mir122 | Control _Non_Heparinized | Hep12U | CNonHep.S.Hep12U_THEP41 | 33.61 |
| mir39 | Control _Non_Heparinized | Hep12U | CNonHep.S.Hep12U_THEP41 | 24.84 |
| mir191 | Control _Non_Heparinized | Hep12U | CNonHep.S.Hep12U_THEP41 | 34.94 |
| mir191 | Control _Non_Heparinized | Hep12U | CNonHep.S.Hep12U_THEP41 | 41.99 |
| mir148 | Control _Non_Heparinized | Hep12U | CNonHep.S.Hep12U_THEP41 |  |
| mir148 | Control _Non_Heparinized | Hep12U | CNonHep.S.Hep12U_THEP41 |  |
| mir122 | Control _Non_Heparinized | Hep12U | CNonHep.S.Hep12U_THEP41 | 31.81 |
| mir103 | Control _Heparinized | NoHep | CHep.S.NoHep_THEP41 | 28.69 |
| UniSP4 | Control _Heparinized | NoHep | CHep.S.NoHep_THEP41 | 26.65 |
| UniSP4 | Control _Heparinized | NoHep | CHep.S.NoHep_THEP41 | 26.64 |
| mir122 | Control _Heparinized | NoHep | CHep.S.NoHep_THEP41 | 28.51 |
| mir148 | Control _Heparinized | NoHep | CHep.S.NoHep_THEP41 | 27.18 |
| mir39 | Control _Heparinized | NoHep | CHep.S.NoHep_THEP41 | 25.56 |
| mir39 | Control _Heparinized | NoHep | CHep.S.NoHep_THEP41 | 25.65 |
| mir122 | Control _Heparinized | NoHep | CHep.S.NoHep_THEP41 | 28.27 |
| mir148 | Control _Heparinized | NoHep | CHep.S.NoHep_THEP41 | 27.49 |
| UniSP4 | Control _Heparinized | NoHep | CHep.S.NoHep_THEP41 | 26.71 |
| mir191 | Control _Heparinized | NoHep | CHep.S.NoHep_THEP41 | 30.22 |
| mir191 | Control _Heparinized | NoHep | CHep.S.NoHep_THEP41 | 30.19 |
| mir39 | Control _Heparinized | NoHep | CHep.S.NoHep_THEP41 | 25.7 |
| mir148 | Control _Heparinized | NoHep | CHep.S.NoHep_THEP41 | 26.79 |
| mir103 | Control _Heparinized | NoHep | CHep.S.NoHep_THEP41 | 28.87 |
| mir103 | Control _Heparinized | NoHep | CHep.S.NoHep_THEP41 | 29.32 |
| mir191 | Control _Heparinized | NoHep | CHep.S.NoHep_THEP41 | 30.48 |
| mir122 | Control _Heparinized | NoHep | CHep.S.NoHep_THEP41 | 28.58 |
| mir148 | Control _Heparinized | Hep6U | CHep.S.Hep6U_THEP41 | 27.65 |
| mir39 | Control _Heparinized | Hep6U | CHep.S.Hep6U_THEP41 | 24.77 |
| mir191 | Control _Heparinized | Hep6U | CHep.S.Hep6U_THEP41 | 30.53 |
| UniSP4 | Control _Heparinized | Hep6U | CHep.S.Hep6U_THEP41 | 26.66 |
| mir148 | Control _Heparinized | Hep6U | CHep.S.Hep6U_THEP41 | 27.79 |
| UniSP4 | Control _Heparinized | Hep6U | CHep.S.Hep6U_THEP41 | 26.9 |
| mir103 | Control _Heparinized | Hep6U | CHep.S.Hep6U_THEP41 | 28.43 |
| mir191 | Control _Heparinized | Hep6U | CHep.S.Hep6U_THEP41 | 30.55 |
| mir39 | Control _Heparinized | Hep6U | CHep.S.Hep6U_THEP41 | 24.67 |
| mir122 | Control _Heparinized | Hep6U | CHep.S.Hep6U_THEP41 | 28.84 |
| mir103 | Control _Heparinized | Hep6U | CHep.S.Hep6U_THEP41 | 28.36 |
| mir39 | Control _Heparinized | Hep6U | CHep.S.Hep6U_THEP41 | 24.61 |
| mir122 | Control _Heparinized | Hep6U | CHep.S.Hep6U_THEP41 | 29.68 |
| mir122 | Control _Heparinized | Hep6U | CHep.S.Hep6U_THEP41 | 29.45 |
| UniSP4 | Control _Heparinized | Hep6U | CHep.S.Hep6U_THEP41 | 26.78 |
| mir103 | Control _Heparinized | Hep6U | CHep.S.Hep6U_THEP41 | 28.37 |
| mir191 | Control _Heparinized | Hep6U | CHep.S.Hep6U_THEP41 | 30.6 |
| mir148 | Control _Heparinized | Hep6U | CHep.S.Hep6U_THEP41 | 27.56 |
| mir122 | Control _Heparinized | Hep12U | CHep.S.Hep12U_THEP41 | 30.32 |
| mir122 | Control _Heparinized | Hep12U | CHep.S.Hep12U_THEP41 | 30.81 |
| mir103 | Control _Heparinized | Hep12U | CHep.S.Hep12U_THEP41 | 30.62 |
| UniSP4 | Control _Heparinized | Hep12U | CHep.S.Hep12U_THEP41 | 29.36 |
| mir191 | Control _Heparinized | Hep12U | CHep.S.Hep12U_THEP41 | 31.64 |
| UniSP4 | Control _Heparinized | Hep12U | CHep.S.Hep12U_THEP41 | 29.13 |
| mir122 | Control _Heparinized | Hep12U | CHep.S.Hep12U_THEP41 | 30.33 |
| mir39 | Control _Heparinized | Hep12U | CHep.S.Hep12U_THEP41 | 24.88 |
| mir191 | Control _Heparinized | Hep12U | CHep.S.Hep12U_THEP41 | 32.36 |
| mir39 | Control _Heparinized | Hep12U | CHep.S.Hep12U_THEP41 | 24.81 |
| mir148 | Control _Heparinized | Hep12U | CHep.S.Hep12U_THEP41 | 31.72 |
| mir39 | Control _Heparinized | Hep12U | CHep.S.Hep12U_THEP41 | 24.69 |
| mir103 | Control _Heparinized | Hep12U | CHep.S.Hep12U_THEP41 | 31.71 |
| mir148 | Control _Heparinized | Hep12U | CHep.S.Hep12U_THEP41 | 30.3 |
| UniSP4 | Control _Heparinized | Hep12U | CHep.S.Hep12U_THEP41 | 28.88 |
| mir103 | Control _Heparinized | Hep12U | CHep.S.Hep12U_THEP41 | 30.76 |
| mir191 | Control _Heparinized | Hep12U | CHep.S.Hep12U_THEP41 | 31.95 |
| mir148 | Control _Heparinized | Hep12U | CHep.S.Hep12U_THEP41 | 30.35 |
| mir191 | Control _Non_Heparinized | NoHep | CNonHep.S.NoHep_THEP42 | 27.64 |
| mir103 | Control _Non_Heparinized | NoHep | CNonHep.S.NoHep_THEP42 | 25.83 |
| mir39 | Control _Non_Heparinized | NoHep | CNonHep.S.NoHep_THEP42 | 24.73 |
| mir103 | Control _Non_Heparinized | NoHep | CNonHep.S.NoHep_THEP42 | 25.92 |
| mir191 | Control _Non_Heparinized | NoHep | CNonHep.S.NoHep_THEP42 | 27.74 |
| UniSP4 | Control _Non_Heparinized | NoHep | CNonHep.S.NoHep_THEP42 | 27.17 |
| mir39 | Control _Non_Heparinized | NoHep | CNonHep.S.NoHep_THEP42 | 24.93 |
| mir39 | Control _Non_Heparinized | NoHep | CNonHep.S.NoHep_THEP42 | 24.88 |
| mir148 | Control _Non_Heparinized | NoHep | CNonHep.S.NoHep_THEP42 | 27.41 |
| mir148 | Control _Non_Heparinized | NoHep | CNonHep.S.NoHep_THEP42 | 27.31 |
| mir148 | Control _Non_Heparinized | NoHep | CNonHep.S.NoHep_THEP42 | 27.37 |
| UniSP4 | Control _Non_Heparinized | NoHep | CNonHep.S.NoHep_THEP42 | 27.4 |
| mir122 | Control _Non_Heparinized | NoHep | CNonHep.S.NoHep_THEP42 | 30.6 |
| mir103 | Control _Non_Heparinized | NoHep | CNonHep.S.NoHep_THEP42 | 25.78 |
| mir122 | Control _Non_Heparinized | NoHep | CNonHep.S.NoHep_THEP42 | 30.35 |
| UniSP4 | Control _Non_Heparinized | NoHep | CNonHep.S.NoHep_THEP42 | 27.36 |
| mir191 | Control _Non_Heparinized | NoHep | CNonHep.S.NoHep_THEP42 | 27.57 |
| mir122 | Control _Non_Heparinized | NoHep | CNonHep.S.NoHep_THEP42 | 30.54 |
| UniSP4 | Control _Non_Heparinized | Hep6U | CNonHep.S.Hep6U_THEP42 | 29.94 |
| mir103 | Control _Non_Heparinized | Hep6U | CNonHep.S.Hep6U_THEP42 | 28.58 |
| mir39 | Control _Non_Heparinized | Hep6U | CNonHep.S.Hep6U_THEP42 | 24.6 |
| mir148 | Control _Non_Heparinized | Hep6U | CNonHep.S.Hep6U_THEP42 | 33.68 |
| mir148 | Control _Non_Heparinized | Hep6U | CNonHep.S.Hep6U_THEP42 | 32.6 |
| UniSP4 | Control _Non_Heparinized | Hep6U | CNonHep.S.Hep6U_THEP42 | 29.93 |
| mir122 | Control _Non_Heparinized | Hep6U | CNonHep.S.Hep6U_THEP42 | 31.91 |
| mir148 | Control _Non_Heparinized | Hep6U | CNonHep.S.Hep6U_THEP42 | 32.71 |
| mir122 | Control _Non_Heparinized | Hep6U | CNonHep.S.Hep6U_THEP42 | 31.9 |
| mir103 | Control _Non_Heparinized | Hep6U | CNonHep.S.Hep6U_THEP42 | 28.75 |
| mir122 | Control _Non_Heparinized | Hep6U | CNonHep.S.Hep6U_THEP42 | 33.71 |
| mir39 | Control _Non_Heparinized | Hep6U | CNonHep.S.Hep6U_THEP42 | 24.52 |
| mir39 | Control _Non_Heparinized | Hep6U | CNonHep.S.Hep6U_THEP42 | 24.46 |
| mir191 | Control _Non_Heparinized | Hep6U | CNonHep.S.Hep6U_THEP42 | 29.82 |
| mir191 | Control _Non_Heparinized | Hep6U | CNonHep.S.Hep6U_THEP42 | 29.36 |
| UniSP4 | Control _Non_Heparinized | Hep6U | CNonHep.S.Hep6U_THEP42 | 29.91 |
| mir191 | Control _Non_Heparinized | Hep6U | CNonHep.S.Hep6U_THEP42 | 30.24 |
| mir103 | Control _Non_Heparinized | Hep6U | CNonHep.S.Hep6U_THEP42 | 28.82 |
| mir39 | Control _Non_Heparinized | Hep12U | CNonHep.S.Hep12U_THEP42 | 24.55 |
| mir39 | Control _Non_Heparinized | Hep12U | CNonHep.S.Hep12U_THEP42 | 24.85 |
| mir39 | Control _Non_Heparinized | Hep12U | CNonHep.S.Hep12U_THEP42 | 24.78 |
| UniSP4 | Control _Non_Heparinized | Hep12U | CNonHep.S.Hep12U_THEP42 | 29.75 |
| mir148 | Control _Non_Heparinized | Hep12U | CNonHep.S.Hep12U_THEP42 | 31.74 |
| mir103 | Control _Non_Heparinized | Hep12U | CNonHep.S.Hep12U_THEP42 | 28.77 |
| mir191 | Control _Non_Heparinized | Hep12U | CNonHep.S.Hep12U_THEP42 | 29.78 |
| mir122 | Control _Non_Heparinized | Hep12U | CNonHep.S.Hep12U_THEP42 | 32.51 |
| UniSP4 | Control _Non_Heparinized | Hep12U | CNonHep.S.Hep12U_THEP42 | 30.43 |
| mir103 | Control _Non_Heparinized | Hep12U | CNonHep.S.Hep12U_THEP42 | 29.29 |
| mir191 | Control _Non_Heparinized | Hep12U | CNonHep.S.Hep12U_THEP42 | 29.7 |
| mir122 | Control _Non_Heparinized | Hep12U | CNonHep.S.Hep12U_THEP42 | 33.86 |
| mir122 | Control _Non_Heparinized | Hep12U | CNonHep.S.Hep12U_THEP42 | 31.85 |
| UniSP4 | Control _Non_Heparinized | Hep12U | CNonHep.S.Hep12U_THEP42 | 29.83 |
| mir103 | Control _Non_Heparinized | Hep12U | CNonHep.S.Hep12U_THEP42 | 29.6 |
| mir148 | Control _Non_Heparinized | Hep12U | CNonHep.S.Hep12U_THEP42 | 31.91 |
| mir191 | Control _Non_Heparinized | Hep12U | CNonHep.S.Hep12U_THEP42 | 30.22 |
| mir148 | Control _Non_Heparinized | Hep12U | CNonHep.S.Hep12U_THEP42 | 32.58 |
| mir148 | Control _Heparinized | NoHep | CHep.S.NoHep_THEP42 | 26.64 |
| mir103 | Control _Heparinized | NoHep | CHep.S.NoHep_THEP42 | 28.28 |
| UniSP4 | Control _Heparinized | NoHep | CHep.S.NoHep_THEP42 | 27.83 |
| mir122 | Control _Heparinized | NoHep | CHep.S.NoHep_THEP42 | 30.56 |
| mir39 | Control _Heparinized | NoHep | CHep.S.NoHep_THEP42 | 24.9 |
| UniSP4 | Control _Heparinized | NoHep | CHep.S.NoHep_THEP42 | 27.83 |
| mir148 | Control _Heparinized | NoHep | CHep.S.NoHep_THEP42 | 26.26 |
| mir122 | Control _Heparinized | NoHep | CHep.S.NoHep_THEP42 | 29.77 |
| mir39 | Control _Heparinized | NoHep | CHep.S.NoHep_THEP42 | 25.38 |
| mir122 | Control _Heparinized | NoHep | CHep.S.NoHep_THEP42 | 29.8 |
| mir39 | Control _Heparinized | NoHep | CHep.S.NoHep_THEP42 | 25.44 |
| mir191 | Control _Heparinized | NoHep | CHep.S.NoHep_THEP42 | 28.8 |
| mir103 | Control _Heparinized | NoHep | CHep.S.NoHep_THEP42 | 28.42 |
| mir191 | Control _Heparinized | NoHep | CHep.S.NoHep_THEP42 | 28.95 |
| mir148 | Control _Heparinized | NoHep | CHep.S.NoHep_THEP42 | 26.22 |
| mir103 | Control _Heparinized | NoHep | CHep.S.NoHep_THEP42 | 27.77 |
| mir191 | Control _Heparinized | NoHep | CHep.S.NoHep_THEP42 | 29.2 |
| UniSP4 | Control _Heparinized | NoHep | CHep.S.NoHep_THEP42 | 28.09 |
| mir39 | Control _Heparinized | Hep6U | CHep.S.Hep6U_THEP42 | 24.69 |
| UniSP4 | Control _Heparinized | Hep6U | CHep.S.Hep6U_THEP42 | 32.22 |
| mir39 | Control _Heparinized | Hep6U | CHep.S.Hep6U_THEP42 | 24.5 |
| mir191 | Control _Heparinized | Hep6U | CHep.S.Hep6U_THEP42 | 32.1 |
| mir122 | Control _Heparinized | Hep6U | CHep.S.Hep6U_THEP42 | 32.28 |
| mir148 | Control _Heparinized | Hep6U | CHep.S.Hep6U_THEP42 |  |
| mir191 | Control _Heparinized | Hep6U | CHep.S.Hep6U_THEP42 | 31.71 |
| mir122 | Control _Heparinized | Hep6U | CHep.S.Hep6U_THEP42 | 33.82 |
| mir148 | Control _Heparinized | Hep6U | CHep.S.Hep6U_THEP42 | 35.24 |
| mir122 | Control _Heparinized | Hep6U | CHep.S.Hep6U_THEP42 | 31.73 |
| mir103 | Control _Heparinized | Hep6U | CHep.S.Hep6U_THEP42 | 33.25 |
| UniSP4 | Control _Heparinized | Hep6U | CHep.S.Hep6U_THEP42 | 32.36 |
| mir103 | Control _Heparinized | Hep6U | CHep.S.Hep6U_THEP42 | 32.43 |
| mir191 | Control _Heparinized | Hep6U | CHep.S.Hep6U_THEP42 | 31.85 |
| UniSP4 | Control _Heparinized | Hep6U | CHep.S.Hep6U_THEP42 | 31.89 |
| mir39 | Control _Heparinized | Hep6U | CHep.S.Hep6U_THEP42 | 24.61 |
| mir103 | Control _Heparinized | Hep6U | CHep.S.Hep6U_THEP42 |  |
| mir148 | Control _Heparinized | Hep6U | CHep.S.Hep6U_THEP42 |  |
| UniSP4 | Control _Heparinized | Hep12U | CHep.S.Hep12U_THEP42 | 33.82 |
| mir148 | Control _Heparinized | Hep12U | CHep.S.Hep12U_THEP42 | 31.67 |
| mir103 | Control _Heparinized | Hep12U | CHep.S.Hep12U_THEP42 | 31.2 |
| mir39 | Control _Heparinized | Hep12U | CHep.S.Hep12U_THEP42 | 24.71 |
| mir122 | Control _Heparinized | Hep12U | CHep.S.Hep12U_THEP42 | 33.82 |
| mir103 | Control _Heparinized | Hep12U | CHep.S.Hep12U_THEP42 | 32.95 |
| mir191 | Control _Heparinized | Hep12U | CHep.S.Hep12U_THEP42 | 31.79 |
| mir191 | Control _Heparinized | Hep12U | CHep.S.Hep12U_THEP42 | 31.87 |
| mir148 | Control _Heparinized | Hep12U | CHep.S.Hep12U_THEP42 | 33.64 |
| mir103 | Control _Heparinized | Hep12U | CHep.S.Hep12U_THEP42 | 31.29 |
| mir39 | Control _Heparinized | Hep12U | CHep.S.Hep12U_THEP42 | 24.48 |
| mir122 | Control _Heparinized | Hep12U | CHep.S.Hep12U_THEP42 | 34.59 |
| mir122 | Control _Heparinized | Hep12U | CHep.S.Hep12U_THEP42 | 38.32 |
| mir148 | Control _Heparinized | Hep12U | CHep.S.Hep12U_THEP42 | 31.94 |
| mir39 | Control _Heparinized | Hep12U | CHep.S.Hep12U_THEP42 | 24.65 |
| mir191 | Control _Heparinized | Hep12U | CHep.S.Hep12U_THEP42 | 31.23 |
| UniSP4 | Control _Heparinized | Hep12U | CHep.S.Hep12U_THEP42 | 32.27 |
| UniSP4 | Control _Heparinized | Hep12U | CHep.S.Hep12U_THEP42 | 31.65 |
| mir39 | Control _Non_Heparinized | NoHep | CNonHep.S.NoHep_THEP43 | 24.5 |
| mir122 | Control _Non_Heparinized | NoHep | CNonHep.S.NoHep_THEP43 | 29.28 |
| mir103 | Control _Non_Heparinized | NoHep | CNonHep.S.NoHep_THEP43 | 28.46 |
| mir122 | Control _Non_Heparinized | NoHep | CNonHep.S.NoHep_THEP43 | 29.6 |
| UniSP4 | Control _Non_Heparinized | NoHep | CNonHep.S.NoHep_THEP43 | 27.23 |
| UniSP4 | Control _Non_Heparinized | NoHep | CNonHep.S.NoHep_THEP43 | 27.4 |
| mir39 | Control _Non_Heparinized | NoHep | CNonHep.S.NoHep_THEP43 | 24.44 |
| mir191 | Control _Non_Heparinized | NoHep | CNonHep.S.NoHep_THEP43 | 30.97 |
| UniSP4 | Control _Non_Heparinized | NoHep | CNonHep.S.NoHep_THEP43 | 27.57 |
| mir103 | Control _Non_Heparinized | NoHep | CNonHep.S.NoHep_THEP43 | 28.76 |
| mir148 | Control _Non_Heparinized | NoHep | CNonHep.S.NoHep_THEP43 | 29.99 |
| mir191 | Control _Non_Heparinized | NoHep | CNonHep.S.NoHep_THEP43 | 30.63 |
| mir39 | Control _Non_Heparinized | NoHep | CNonHep.S.NoHep_THEP43 | 24.62 |
| mir148 | Control _Non_Heparinized | NoHep | CNonHep.S.NoHep_THEP43 | 29.66 |
| mir148 | Control _Non_Heparinized | NoHep | CNonHep.S.NoHep_THEP43 | 29.69 |
| mir191 | Control _Non_Heparinized | NoHep | CNonHep.S.NoHep_THEP43 | 31.18 |
| mir122 | Control _Non_Heparinized | NoHep | CNonHep.S.NoHep_THEP43 | 29.67 |
| mir103 | Control _Non_Heparinized | NoHep | CNonHep.S.NoHep_THEP43 | 28.38 |
| mir191 | Control _Non_Heparinized | Hep6U | CNonHep.S.Hep6U_THEP43 | 34.97 |
| UniSP4 | Control _Non_Heparinized | Hep6U | CNonHep.S.Hep6U_THEP43 | 30.5 |
| mir103 | Control _Non_Heparinized | Hep6U | CNonHep.S.Hep6U_THEP43 | 31.22 |
| mir39 | Control _Non_Heparinized | Hep6U | CNonHep.S.Hep6U_THEP43 | 24.59 |
| mir191 | Control _Non_Heparinized | Hep6U | CNonHep.S.Hep6U_THEP43 | 34.92 |
| mir122 | Control _Non_Heparinized | Hep6U | CNonHep.S.Hep6U_THEP43 | 32.69 |
| mir148 | Control _Non_Heparinized | Hep6U | CNonHep.S.Hep6U_THEP43 | 32.29 |
| mir39 | Control _Non_Heparinized | Hep6U | CNonHep.S.Hep6U_THEP43 | 24.47 |
| mir122 | Control _Non_Heparinized | Hep6U | CNonHep.S.Hep6U_THEP43 | 30.84 |
| mir39 | Control _Non_Heparinized | Hep6U | CNonHep.S.Hep6U_THEP43 | 24.77 |
| mir122 | Control _Non_Heparinized | Hep6U | CNonHep.S.Hep6U_THEP43 | 38.71 |
| mir103 | Control _Non_Heparinized | Hep6U | CNonHep.S.Hep6U_THEP43 | 32.19 |
| mir148 | Control _Non_Heparinized | Hep6U | CNonHep.S.Hep6U_THEP43 | 36.4 |
| UniSP4 | Control _Non_Heparinized | Hep6U | CNonHep.S.Hep6U_THEP43 | 29.9 |
| UniSP4 | Control _Non_Heparinized | Hep6U | CNonHep.S.Hep6U_THEP43 | 30.8 |
| mir103 | Control _Non_Heparinized | Hep6U | CNonHep.S.Hep6U_THEP43 |  |
| mir191 | Control _Non_Heparinized | Hep6U | CNonHep.S.Hep6U_THEP43 | 32.9 |
| mir148 | Control _Non_Heparinized | Hep6U | CNonHep.S.Hep6U_THEP43 | 34.59 |
| mir103 | Control _Non_Heparinized | Hep12U | CNonHep.S.Hep12U_THEP43 | 32.94 |
| mir122 | Control _Non_Heparinized | Hep12U | CNonHep.S.Hep12U_THEP43 | 32.62 |
| UniSP4 | Control _Non_Heparinized | Hep12U | CNonHep.S.Hep12U_THEP43 | 31.6 |
| mir39 | Control _Non_Heparinized | Hep12U | CNonHep.S.Hep12U_THEP43 | 24.49 |
| mir39 | Control _Non_Heparinized | Hep12U | CNonHep.S.Hep12U_THEP43 | 24.68 |
| mir122 | Control _Non_Heparinized | Hep12U | CNonHep.S.Hep12U_THEP43 | 31.86 |
| mir103 | Control _Non_Heparinized | Hep12U | CNonHep.S.Hep12U_THEP43 | 32.29 |
| mir148 | Control _Non_Heparinized | Hep12U | CNonHep.S.Hep12U_THEP43 | 33.47 |
| mir191 | Control _Non_Heparinized | Hep12U | CNonHep.S.Hep12U_THEP43 | 41.46 |
| mir191 | Control _Non_Heparinized | Hep12U | CNonHep.S.Hep12U_THEP43 | 33.84 |
| UniSP4 | Control _Non_Heparinized | Hep12U | CNonHep.S.Hep12U_THEP43 | 32.6 |
| mir148 | Control _Non_Heparinized | Hep12U | CNonHep.S.Hep12U_THEP43 |  |
| mir191 | Control _Non_Heparinized | Hep12U | CNonHep.S.Hep12U_THEP43 | 34.74 |
| mir122 | Control _Non_Heparinized | Hep12U | CNonHep.S.Hep12U_THEP43 | 32.57 |
| UniSP4 | Control _Non_Heparinized | Hep12U | CNonHep.S.Hep12U_THEP43 | 31.68 |
| mir148 | Control _Non_Heparinized | Hep12U | CNonHep.S.Hep12U_THEP43 |  |
| mir103 | Control _Non_Heparinized | Hep12U | CNonHep.S.Hep12U_THEP43 | 31.93 |
| mir39 | Control _Non_Heparinized | Hep12U | CNonHep.S.Hep12U_THEP43 | 24.59 |
| mir103 | Control _Heparinized | NoHep | CHep.S.NoHep_THEP43 | 28.4 |
| mir122 | Control _Heparinized | NoHep | CHep.S.NoHep_THEP43 | 26.2 |
| UniSP4 | Control _Heparinized | NoHep | CHep.S.NoHep_THEP43 | 27.41 |
| mir148 | Control _Heparinized | NoHep | CHep.S.NoHep_THEP43 | 26.71 |
| mir191 | Control _Heparinized | NoHep | CHep.S.NoHep_THEP43 | 29.46 |
| mir191 | Control _Heparinized | NoHep | CHep.S.NoHep_THEP43 | 29.42 |
| mir39 | Control _Heparinized | NoHep | CHep.S.NoHep_THEP43 | 25.43 |
| mir122 | Control _Heparinized | NoHep | CHep.S.NoHep_THEP43 | 26.38 |
| mir191 | Control _Heparinized | NoHep | CHep.S.NoHep_THEP43 | 29.52 |
| mir39 | Control _Heparinized | NoHep | CHep.S.NoHep_THEP43 | 25.32 |
| UniSP4 | Control _Heparinized | NoHep | CHep.S.NoHep_THEP43 | 27.92 |
| mir103 | Control _Heparinized | NoHep | CHep.S.NoHep_THEP43 | 28 |
| mir103 | Control _Heparinized | NoHep | CHep.S.NoHep_THEP43 | 28.34 |
| mir39 | Control _Heparinized | NoHep | CHep.S.NoHep_THEP43 | 25.33 |
| mir122 | Control _Heparinized | NoHep | CHep.S.NoHep_THEP43 | 26.42 |
| mir148 | Control _Heparinized | NoHep | CHep.S.NoHep_THEP43 | 25.87 |
| mir148 | Control _Heparinized | NoHep | CHep.S.NoHep_THEP43 | 26.43 |
| UniSP4 | Control _Heparinized | NoHep | CHep.S.NoHep_THEP43 | 27.94 |
| mir39 | Control _Heparinized | Hep6U | CHep.S.Hep6U_THEP43 | 24.81 |
| mir122 | Control _Heparinized | Hep6U | CHep.S.Hep6U_THEP43 | 32.57 |
| mir191 | Control _Heparinized | Hep6U | CHep.S.Hep6U_THEP43 |  |
| mir122 | Control _Heparinized | Hep6U | CHep.S.Hep6U_THEP43 | 33.36 |
| mir103 | Control _Heparinized | Hep6U | CHep.S.Hep6U_THEP43 | 32.93 |
| mir191 | Control _Heparinized | Hep6U | CHep.S.Hep6U_THEP43 |  |
| UniSP4 | Control _Heparinized | Hep6U | CHep.S.Hep6U_THEP43 |  |
| mir103 | Control _Heparinized | Hep6U | CHep.S.Hep6U_THEP43 |  |
| UniSP4 | Control _Heparinized | Hep6U | CHep.S.Hep6U_THEP43 | 33.27 |
| mir39 | Control _Heparinized | Hep6U | CHep.S.Hep6U_THEP43 | 24.74 |
| mir39 | Control _Heparinized | Hep6U | CHep.S.Hep6U_THEP43 | 24.9 |
| mir103 | Control _Heparinized | Hep6U | CHep.S.Hep6U_THEP43 | 39.94 |
| mir148 | Control _Heparinized | Hep6U | CHep.S.Hep6U_THEP43 |  |
| mir148 | Control _Heparinized | Hep6U | CHep.S.Hep6U_THEP43 |  |
| UniSP4 | Control _Heparinized | Hep6U | CHep.S.Hep6U_THEP43 | 34.79 |
| mir148 | Control _Heparinized | Hep6U | CHep.S.Hep6U_THEP43 |  |
| mir122 | Control _Heparinized | Hep6U | CHep.S.Hep6U_THEP43 | 32.64 |
| mir191 | Control _Heparinized | Hep6U | CHep.S.Hep6U_THEP43 |  |
| mir103 | Control _Heparinized | Hep12U | CHep.S.Hep12U_THEP43 |  |
| mir191 | Control _Heparinized | Hep12U | CHep.S.Hep12U_THEP43 |  |
| UniSP4 | Control _Heparinized | Hep12U | CHep.S.Hep12U_THEP43 | 33.94 |
| mir191 | Control _Heparinized | Hep12U | CHep.S.Hep12U_THEP43 | 34.79 |
| mir39 | Control _Heparinized | Hep12U | CHep.S.Hep12U_THEP43 | 24.69 |
| mir148 | Control _Heparinized | Hep12U | CHep.S.Hep12U_THEP43 | 33.37 |
| UniSP4 | Control _Heparinized | Hep12U | CHep.S.Hep12U_THEP43 |  |
| mir103 | Control _Heparinized | Hep12U | CHep.S.Hep12U_THEP43 |  |
| mir122 | Control _Heparinized | Hep12U | CHep.S.Hep12U_THEP43 | 30.94 |
| mir122 | Control _Heparinized | Hep12U | CHep.S.Hep12U_THEP43 | 31.82 |
| UniSP4 | Control _Heparinized | Hep12U | CHep.S.Hep12U_THEP43 | 33.67 |
| mir39 | Control _Heparinized | Hep12U | CHep.S.Hep12U_THEP43 | 24.62 |
| mir191 | Control _Heparinized | Hep12U | CHep.S.Hep12U_THEP43 |  |
| mir122 | Control _Heparinized | Hep12U | CHep.S.Hep12U_THEP43 | 33.53 |
| mir148 | Control _Heparinized | Hep12U | CHep.S.Hep12U_THEP43 | 33.72 |
| mir39 | Control _Heparinized | Hep12U | CHep.S.Hep12U_THEP43 | 24.68 |
| mir103 | Control _Heparinized | Hep12U | CHep.S.Hep12U_THEP43 |  |
| mir148 | Control _Heparinized | Hep12U | CHep.S.Hep12U_THEP43 |  |
| UniSP4 | Control _Non_Heparinized | NoHep | CNonHep.S.NoHep_THEP44 | 28.82 |
| mir122 | Control _Non_Heparinized | NoHep | CNonHep.S.NoHep_THEP44 | 29.52 |
| mir191 | Control _Non_Heparinized | NoHep | CNonHep.S.NoHep_THEP44 | 29.26 |
| mir191 | Control _Non_Heparinized | NoHep | CNonHep.S.NoHep_THEP44 | 29.35 |
| mir103 | Control _Non_Heparinized | NoHep | CNonHep.S.NoHep_THEP44 | 27.73 |
| mir103 | Control _Non_Heparinized | NoHep | CNonHep.S.NoHep_THEP44 | 27.92 |
| mir39 | Control _Non_Heparinized | NoHep | CNonHep.S.NoHep_THEP44 | 24.45 |
| UniSP4 | Control _Non_Heparinized | NoHep | CNonHep.S.NoHep_THEP44 | 28.97 |
| mir103 | Control _Non_Heparinized | NoHep | CNonHep.S.NoHep_THEP44 | 28 |
| mir148 | Control _Non_Heparinized | NoHep | CNonHep.S.NoHep_THEP44 | 29.86 |
| mir39 | Control _Non_Heparinized | NoHep | CNonHep.S.NoHep_THEP44 | 24.54 |
| UniSP4 | Control _Non_Heparinized | NoHep | CNonHep.S.NoHep_THEP44 | 28.73 |
| mir122 | Control _Non_Heparinized | NoHep | CNonHep.S.NoHep_THEP44 | 28.94 |
| mir39 | Control _Non_Heparinized | NoHep | CNonHep.S.NoHep_THEP44 | 24.42 |
| mir148 | Control _Non_Heparinized | NoHep | CNonHep.S.NoHep_THEP44 | 29.76 |
| mir122 | Control _Non_Heparinized | NoHep | CNonHep.S.NoHep_THEP44 | 28.65 |
| mir148 | Control _Non_Heparinized | NoHep | CNonHep.S.NoHep_THEP44 | 29.66 |
| mir191 | Control _Non_Heparinized | NoHep | CNonHep.S.NoHep_THEP44 | 29.24 |
| mir191 | Control _Non_Heparinized | Hep6U | CNonHep.S.Hep6U_THEP44 |  |
| mir103 | Control _Non_Heparinized | Hep6U | CNonHep.S.Hep6U_THEP44 |  |
| UniSP4 | Control _Non_Heparinized | Hep6U | CNonHep.S.Hep6U_THEP44 |  |
| mir148 | Control _Non_Heparinized | Hep6U | CNonHep.S.Hep6U_THEP44 | 33.76 |
| UniSP4 | Control _Non_Heparinized | Hep6U | CNonHep.S.Hep6U_THEP44 |  |
| mir103 | Control _Non_Heparinized | Hep6U | CNonHep.S.Hep6U_THEP44 |  |
| mir122 | Control _Non_Heparinized | Hep6U | CNonHep.S.Hep6U_THEP44 | 34.58 |
| UniSP4 | Control _Non_Heparinized | Hep6U | CNonHep.S.Hep6U_THEP44 |  |
| mir122 | Control _Non_Heparinized | Hep6U | CNonHep.S.Hep6U_THEP44 |  |
| mir122 | Control _Non_Heparinized | Hep6U | CNonHep.S.Hep6U_THEP44 |  |
| mir191 | Control _Non_Heparinized | Hep6U | CNonHep.S.Hep6U_THEP44 | 34.86 |
| mir148 | Control _Non_Heparinized | Hep6U | CNonHep.S.Hep6U_THEP44 |  |
| mir39 | Control _Non_Heparinized | Hep6U | CNonHep.S.Hep6U_THEP44 | 24.63 |
| mir148 | Control _Non_Heparinized | Hep6U | CNonHep.S.Hep6U_THEP44 | 34.31 |
| mir39 | Control _Non_Heparinized | Hep6U | CNonHep.S.Hep6U_THEP44 | 24.75 |
| mir39 | Control _Non_Heparinized | Hep6U | CNonHep.S.Hep6U_THEP44 | 24.67 |
| mir191 | Control _Non_Heparinized | Hep6U | CNonHep.S.Hep6U_THEP44 | 34.72 |
| mir103 | Control _Non_Heparinized | Hep6U | CNonHep.S.Hep6U_THEP44 |  |
| mir191 | Control _Non_Heparinized | Hep12U | CNonHep.S.Hep12U_THEP44 | 34.8 |
| UniSP4 | Control _Non_Heparinized | Hep12U | CNonHep.S.Hep12U_THEP44 | 38.66 |
| mir122 | Control _Non_Heparinized | Hep12U | CNonHep.S.Hep12U_THEP44 | 33.78 |
| mir39 | Control _Non_Heparinized | Hep12U | CNonHep.S.Hep12U_THEP44 | 24.59 |
| mir191 | Control _Non_Heparinized | Hep12U | CNonHep.S.Hep12U_THEP44 | 33.54 |
| mir103 | Control _Non_Heparinized | Hep12U | CNonHep.S.Hep12U_THEP44 |  |
| mir103 | Control _Non_Heparinized | Hep12U | CNonHep.S.Hep12U_THEP44 |  |
| mir122 | Control _Non_Heparinized | Hep12U | CNonHep.S.Hep12U_THEP44 | 32.52 |
| mir103 | Control _Non_Heparinized | Hep12U | CNonHep.S.Hep12U_THEP44 | 33.32 |
| mir39 | Control _Non_Heparinized | Hep12U | CNonHep.S.Hep12U_THEP44 | 24.63 |
| mir148 | Control _Non_Heparinized | Hep12U | CNonHep.S.Hep12U_THEP44 | 32.6 |
| mir148 | Control _Non_Heparinized | Hep12U | CNonHep.S.Hep12U_THEP44 |  |
| UniSP4 | Control _Non_Heparinized | Hep12U | CNonHep.S.Hep12U_THEP44 | 33.83 |
| UniSP4 | Control _Non_Heparinized | Hep12U | CNonHep.S.Hep12U_THEP44 | 33.74 |
| mir39 | Control _Non_Heparinized | Hep12U | CNonHep.S.Hep12U_THEP44 | 24.6 |
| mir191 | Control _Non_Heparinized | Hep12U | CNonHep.S.Hep12U_THEP44 | 33.21 |
| mir122 | Control _Non_Heparinized | Hep12U | CNonHep.S.Hep12U_THEP44 | 31.87 |
| mir148 | Control _Non_Heparinized | Hep12U | CNonHep.S.Hep12U_THEP44 |  |
| mir103 | Control _Heparinized | NoHep | CHep.S.NoHep_THEP44 | 26.99 |
| mir103 | Control _Heparinized | NoHep | CHep.S.NoHep_THEP44 | 26.85 |
| mir191 | Control _Heparinized | NoHep | CHep.S.NoHep_THEP44 | 27.63 |
| mir148 | Control _Heparinized | NoHep | CHep.S.NoHep_THEP44 | 28.6 |
| mir148 | Control _Heparinized | NoHep | CHep.S.NoHep_THEP44 | 26.6 |
| mir148 | Control _Heparinized | NoHep | CHep.S.NoHep_THEP44 | 27.92 |
| mir191 | Control _Heparinized | NoHep | CHep.S.NoHep_THEP44 | 27.34 |
| mir122 | Control _Heparinized | NoHep | CHep.S.NoHep_THEP44 | 27.4 |
| mir39 | Control _Heparinized | NoHep | CHep.S.NoHep_THEP44 | 25.39 |
| mir39 | Control _Heparinized | NoHep | CHep.S.NoHep_THEP44 | 25.41 |
| UniSP4 | Control _Heparinized | NoHep | CHep.S.NoHep_THEP44 | 28.17 |
| mir191 | Control _Heparinized | NoHep | CHep.S.NoHep_THEP44 | 27.36 |
| mir122 | Control _Heparinized | NoHep | CHep.S.NoHep_THEP44 | 27.42 |
| UniSP4 | Control _Heparinized | NoHep | CHep.S.NoHep_THEP44 | 28.3 |
| mir122 | Control _Heparinized | NoHep | CHep.S.NoHep_THEP44 | 27.48 |
| UniSP4 | Control _Heparinized | NoHep | CHep.S.NoHep_THEP44 | 28.32 |
| mir39 | Control _Heparinized | NoHep | CHep.S.NoHep_THEP44 | 24.99 |
| mir103 | Control _Heparinized | NoHep | CHep.S.NoHep_THEP44 | 26.72 |
| mir122 | Control _Heparinized | Hep6U | CHep.S.Hep6U_THEP44 | 33.77 |
| mir39 | Control _Heparinized | Hep6U | CHep.S.Hep6U_THEP44 | 24.49 |
| mir103 | Control _Heparinized | Hep6U | CHep.S.Hep6U_THEP44 |  |
| mir122 | Control _Heparinized | Hep6U | CHep.S.Hep6U_THEP44 |  |
| UniSP4 | Control _Heparinized | Hep6U | CHep.S.Hep6U_THEP44 | 37.93 |
| mir103 | Control _Heparinized | Hep6U | CHep.S.Hep6U_THEP44 | 32.66 |
| mir191 | Control _Heparinized | Hep6U | CHep.S.Hep6U_THEP44 | 31.71 |
| UniSP4 | Control _Heparinized | Hep6U | CHep.S.Hep6U_THEP44 |  |
| mir191 | Control _Heparinized | Hep6U | CHep.S.Hep6U_THEP44 | 31.96 |
| mir122 | Control _Heparinized | Hep6U | CHep.S.Hep6U_THEP44 |  |
| mir103 | Control _Heparinized | Hep6U | CHep.S.Hep6U_THEP44 | 31.91 |
| UniSP4 | Control _Heparinized | Hep6U | CHep.S.Hep6U_THEP44 | 34.95 |
| mir191 | Control _Heparinized | Hep6U | CHep.S.Hep6U_THEP44 | 33.34 |
| mir148 | Control _Heparinized | Hep6U | CHep.S.Hep6U_THEP44 | 33.19 |
| mir148 | Control _Heparinized | Hep6U | CHep.S.Hep6U_THEP44 |  |
| mir39 | Control _Heparinized | Hep6U | CHep.S.Hep6U_THEP44 | 24.68 |
| mir148 | Control _Heparinized | Hep6U | CHep.S.Hep6U_THEP44 |  |
| mir39 | Control _Heparinized | Hep6U | CHep.S.Hep6U_THEP44 | 24.69 |
| UniSP4 | Control _Heparinized | Hep12U | CHep.S.Hep12U_THEP44 | 33.91 |
| mir103 | Control _Heparinized | Hep12U | CHep.S.Hep12U_THEP44 |  |
| UniSP4 | Control _Heparinized | Hep12U | CHep.S.Hep12U_THEP44 | 41.56 |
| mir122 | Control _Heparinized | Hep12U | CHep.S.Hep12U_THEP44 |  |
| mir103 | Control _Heparinized | Hep12U | CHep.S.Hep12U_THEP44 | 31.57 |
| UniSP4 | Control _Heparinized | Hep12U | CHep.S.Hep12U_THEP44 |  |
| mir148 | Control _Heparinized | Hep12U | CHep.S.Hep12U_THEP44 |  |
| mir122 | Control _Heparinized | Hep12U | CHep.S.Hep12U_THEP44 |  |
| mir39 | Control _Heparinized | Hep12U | CHep.S.Hep12U_THEP44 | 24.46 |
| mir148 | Control _Heparinized | Hep12U | CHep.S.Hep12U_THEP44 |  |
| mir122 | Control _Heparinized | Hep12U | CHep.S.Hep12U_THEP44 |  |
| mir191 | Control _Heparinized | Hep12U | CHep.S.Hep12U_THEP44 | 32.71 |
| mir39 | Control _Heparinized | Hep12U | CHep.S.Hep12U_THEP44 | 24.65 |
| mir191 | Control _Heparinized | Hep12U | CHep.S.Hep12U_THEP44 | 32.37 |
| mir103 | Control _Heparinized | Hep12U | CHep.S.Hep12U_THEP44 |  |
| mir39 | Control _Heparinized | Hep12U | CHep.S.Hep12U_THEP44 | 24.48 |
| mir191 | Control _Heparinized | Hep12U | CHep.S.Hep12U_THEP44 | 32.38 |
| mir148 | Control _Heparinized | Hep12U | CHep.S.Hep12U_THEP44 |  |
