# Supplementary material for: Heparinase I treatment to overcome RNA quantification interference in heparinized liver donor samples: One size fits all?
Source: PLoS One. 2025 May 12;20(5):e0322899. doi: 10.1371/journal.pone.0322899 (PMC12068581; doi:10.1371/journal.pone.0322899)

**S1 Fig. Donor miRNA relative expression with or without heparinase I treatment.**

(A) Liver biopsies, (B) Perfusion fluid and (C) Serum from eight liver donors. In liver tissue, the relative expression of miR-122 and miR-148a without heparinase I was significantly higher compared to those treated with 6 IU and 12 IU. Note that serum miRNA quantification experienced a significant loss of data with 0%, 65.6% and 71.9% of non-available (NA) values after treatment with 0 IU, 6 IU and 12 IU respectively.


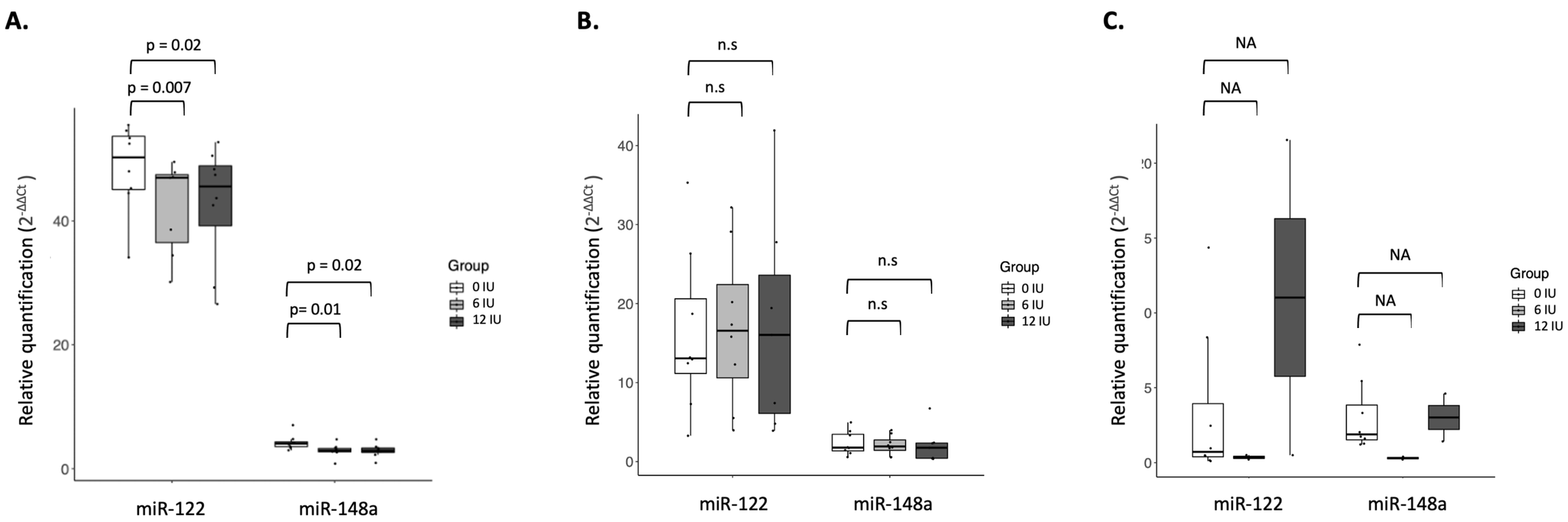

Supplement: S1 Fig — (DOCX) [file pone.0322899.s005.docx]
