# Supplementary material for: Heparinase I treatment to overcome RNA quantification interference in heparinized liver donor samples: One size fits all?
Source: PLoS One. 2025 May 12;20(5):e0322899. doi: 10.1371/journal.pone.0322899 (PMC12068581; doi:10.1371/journal.pone.0322899)
Supplement: Attachment — Submitted filename: Comments.docx [file pone.0322899.s006.docx]

This study is interesting. But there are some problems, which must be solved before it is considered for publication.

1. There are many formatting errors or irregularities in the manuscript, such as the lack of spaces between some numbers and units; irregularities such as RNAse should be RNase (line 395); Many references are incomplete, e.g. missing page numbers (ref 7, 21,22…).

2. The image resolution is subpar, with unclear annotations detracting from the overall clarity and coherence of the visual representation. (Fig.2, 3,4,5,6)

3. Tables should be formatted consistently and in a more scientific three-line table format
